# Cyclo(His-Pro): A further step in the management of steatohepatitis

**DOI:** 10.1016/j.jhepr.2023.100815

**Published:** 2023-06-10

**Authors:** Alessia De Masi, Xiaoxu Li, Dohyun Lee, Jongsu Jeon, Qi Wang, Seoyeong Baek, Onyu Park, Adrienne Mottis, Keno Strotjohann, Alexis Rapin, Hoe-Yune Jung, Johan Auwerx

**Affiliations:** 1 Laboratory of Integrative Systems Physiology, Institute of Bioengineering, École Polytechnique Fédérale de Lausanne, Lausanne, Switzerland; 2 R&D Center, NovMetaPharma Co., Ltd., Pohang, South Korea; 3 School of Interdisciplinary Bioscience and Bioengineering, Pohang University of Science and Technology (POSTECH), Pohang, South Korea

**Keywords:** Cyclo histidine-proline (Cyclo(His-Pro)), Non-alcoholic fatty liver disease, Non-alcoholic steatohepatitis, Liver, Transcriptomics, Prevention, Inflammation, Liver fibrosis, Therapeutics, Drug development

## Abstract

**Background & Aims:**

Non-alcoholic fatty liver disease (NAFLD) and steatohepatitis (NASH) have become the world's most common liver diseases, placing a growing strain on healthcare systems worldwide. Nonetheless, no effective pharmacological treatment has been approved. The naturally occurring compound cyclo histidine-proline (His-Pro) (CHP) is an interesting candidate for NAFLD management, given its safety profile and anti-inflammatory effects.

**Methods:**

Two different mouse models of liver disease were used to evaluate protective effects of CHP on disease progression towards fibrosis: a model of dietary NAFLD/NASH, achieved by thermoneutral housing (TN) in combination with feeding a western diet (WD), and liver fibrosis caused by repeated injections with carbon tetrachloride (CCl_4_).

**Results:**

Treatment with CHP limited overall lipid accumulation, lowered systemic inflammation, and prevented hyperglycaemia. Histopathology and liver transcriptomics highlighted reduced steatosis and demonstrated remarkable protection from the development of inflammation and fibrosis, features which herald the progression of NAFLD. We identified the extracellular signal-regulated kinase (ERK) pathway as an early mediator of the cellular response to CHP.

**Conclusions:**

CHP was active in both the preventive and therapeutic setting, reducing liver steatosis, fibrosis, and inflammation and improving several markers of liver disease.

**Impact and implications:**

Considering the incidence and the lack of approved treatments, it is urgent to identify new strategies that prevent and manage NAFLD. CHP was effective in attenuating NAFLD progression in two animal models of the disease. Overall, our work points to CHP as a novel and effective strategy for the management of NAFLD, fuelling optimism for potential clinical studies.

## Highlights

• CHP prevents NAFLD progression toward NASH.
• CHP reduces liver steatosis, fibrosis, inflammation and disease markers.
• Liver transcriptomics shows the molecular signature of CHP.
• CHP is effective against liver disease both in preventive and therapeutic settings.
• CHP inhibits ERK signalling and enhances mitochondrial function in the liver.

## Graphical abstract

**Figure:**
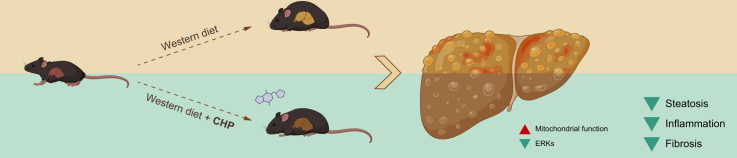

## Introduction

Non-alcoholic fatty liver disease (NAFLD) is the most common liver disease worldwide, currently affecting 25% of the adult population.[1], [2], [3] NAFLD development is closely intertwined with obesity, type 2 diabetes, impaired lipid metabolism, and insulin resistance, conditions whose incidence have also increased over the past decades.^4^^,^^5^ NAFLD refers to a broad spectrum of liver histological alterations, typified by hepatocellular steatosis, that ranges from isolated steatosis to non-alcoholic steatohepatitis (NASH).^6^ Statistically, 30% of patients with NAFLD will develop NASH, characterised by additional hepatocellular ballooning and inflammation.^7^^,^^8^ If left untreated, NAFLD can eventually progress to liver fibrosis, cirrhosis, and even hepatocellular carcinoma.^9^^,^^10^ Currently, there are no FDA-approved treatments, and the best therapeutic option available is based on lifestyle changes, which are often hindered by lack of compliance.^11^ It is therefore urgent to find safe and effective strategies to manage hepatic lipid accumulation, chronic inflammation, and fibrosis in patients with NAFLD.

Cyclo histidine-proline (His-Pro) (CHP) is an endogenous cyclic dipeptide derived from the thyrotropin-releasing hormone (TRH).^12^ Pyroglutamyl-peptidase degrades TRH and forms His-Pro dipeptides, which undergo spontaneous cyclisation at 37 °C.^13^^,^^14^ This cyclisation confers higher stability and protection against peptidases. This is required for its active transport in the intestine^15^ and through the blood–brain barrier,^16^^,^^17^ allowing CHP to be distributed systemically.^18^ CHP is present in several food sources^19^ and can be safely administered *per os.*^20^^,^^21^ CHP started to gain scientific interest as several studies outlined its cytoprotective role in neuronal models.[22], [23], [24], [25] CHP has also been evaluated as a drug candidate in the context of diabetes, as it helps to control blood glucose levels and insulin response.[26], [27], [28], [29] At the molecular level, CHP is known to trigger anti-oxidant^30^ and anti-inflammatory responses;^31^ however, the biological role of CHP, as well as its mechanism of action, have remained elusive.

In this study, we evaluated whether CHP administration could prevent inflammation and fibrosis in liver disease. Using a murine model of NAFLD, we demonstrated that CHP counteracts weight gain and body fat accumulation. Administration of CHP improved the overall liver phenotype, including reduced liver steatosis, fibrosis, and inflammation. The efficacy of the treatment was confirmed in a second liver injury model, which was induced by repeated low-dose carbon tetrachloride (CCl_4_) injections. In this model, widely used to study liver fibrosis, we provide a first indication that CHP not only has the potential to prevent liver disease, but it can also be used as a therapeutic treatment. Thus, our *in vivo* studies provide a rationale for both the preventive and the therapeutic effects of CHP in the context of NAFLD/NASH. Transcriptomic and molecular studies confirmed the robust anti-inflammatory effects of CHP, leading us to identify the extracellular signal-regulated kinase (ERK) cascade as a relevant signalling pathway involved in the response to CHP treatment and its impact on mitochondrial function.

## Materials and methods

### Animal studies

#### Diet-induced NAFLD

C57BL/6J male mice (Charles River) were transferred to thermoneutral housing (TN) cabinets (30–32 °C) at 6 weeks of age. At Week 7, the animals received the chow diet (CD) (Research Diet D16042904B) or the western diet (WD) (Research Diet D12079B). Treatment with CHP was started at Week 7, 20 mg/kg doses were administered 3 times a week *per os* (gavage) throughout the study. Water was used as a control treatment. Body weight was monitored weekly. Phenotyping started at Week 18 with a non-invasive monitoring of fat and lean mass using the Echo Medical Systems magnetic resonance imaging (MRI) device as previously described.^32^ Food intake, caloric consumption, and respiratory quotient were measured using Promethion cages (Sable Systems). At Week 20, mice were subjected to an overnight fast. Tail vein glucose levels were measured using the CONTOUR®NEXT glucometer (Ascensia Diabetes Care). Insulin levels were measured with the Ultra-Sensitive Mouse Insulin ELISA Kit (Crystal Chem). Homeostatic model assessment (HOMA)^33^ was used to calculate the insulin-resistance (IR) index as follows: HOMA-IR = [fasting blood glucose (mmol/L) × serum insulin (mIU/L)]/22.5. At Week 24, after a 3-h fast, mice were euthanised to collect tissues, of which the weight was measured. For biochemical analysis, tissues were collected and flash-frozen, and stored at −80 °C. In a follow-up study, mice were housed at TN, fed with either CD or WD and treated with CHP for 8 weeks. Faeces were collected at Week 7 of the study for a 24-h period and caloric content was quantified with a calorimeter bomb (IKA C5003, IKA-Werke GmbH & Co. KG, Staufen, Germany). To study the ERK response, 6-week-old C57BL/6J male mice were treated with 20 mg/kg of CHP *per os*. Water was used as a control treatment. Mice were euthanised to collect the liver at 4 or 24 h after treatment.

#### CCl_4_-induced liver fibrosis

Eight-week-old male C57BL/6 mice (Koatech Co. Ltd.) were housed at 23 °C with free to access to water and chow diet *ad libitum*. After a week of adaptation, the mice were divided into 3 groups: 0.2 ml/kg of CCl_4_ in olive oil (CCl_4_:olive oil = 1:19 vol:vol) was injected intraperitoneally (*i.p*.) on Days 1, 3, 6, 8, 10, and 13 for the 2-week study and on Days 1, 3, 6, 8, 10, 13, 15, 17, and 20 for the 3-week study; the control group received *i.p.* injection of an equal volume of olive oil. Distilled water (vehicle) or 35 mg/kg of CHP were administered daily by oral gavage, after the first (preventive study) or third (therapeutic study) CCl_4_ injection. Mice were euthanised 12 h after the last CCl_4_ injection, to collect plasma and tissues.

### RNA-seq

In the NAFLD study, RNA was extracted in Trizol (TriPure, Roche) from frozen liver, kidney, or gastrocnemius tissue, using the Direct-zol-96 RNA Kit (Zymo Research). Bulk RNA-seq of extracted RNA from each mouse was performed by the Beijing Genomics Institute with the BGISEQ-500 platform. In the CCl_4_ study, RNA was extracted with NucleoZOL reagent (Macherey-Nagel) and paired-end sequencing was performed by Macrogen Incorporated with Illumina NovaSeq. Clean reads were obtained by removing adapter sequences or low-quality sequences (RIN <8) based on SOAPnuke software.^34^ RNA-seq data and related analyses were performed using the R version 3.5.2.^35^ All samples passed the quality check by FastQC.[36], [37] Sequences were aligned against the mouse genome, using STAR (version 2.7.3a).^38^ Two comparisons (WD *vs.* CD, WD + CHP *vs.* WD for the NAFLD study; CCl_4_ *vs.* control [CTRL], CCl_4_ + CHP *vs.* CCl_4_ for the CCl_4_-induced model) were analysed by differential expression analysis (DEA) using the limma R package (version 3.38.3).^39^^,^^40^ Further detailed information for the RNA-seq analysis can be found in the Supplementary methods.

### Cell experiments

AML-12 cell line was obtained from ATCC and grown at 37 °C in a humidified atmosphere of 5% CO_2_ and 95% air in DMEM/F12 supplemented with FBS 10% (Gibco), 0.005 mg/ml insulin, 0.005 mg/ml transferrin, 5 ng/ml selenium, and 40 ng/ml dexamethasone. Cells were treated with CHP 100 nM for 4 or 24 h, then samples were recovered for protein extraction.

### Statistics

GraphPad Prism v9.5.1 was used for all statistical analyses. Differences between groups were assessed using the unpaired *t*-test, one-way or two-way analysis of variance (ANOVA), followed by the Dunnett multiple comparison test, as specified in the figure legends. All *p* values < 0.05 were considered significant.

### Study approval

The WD/TN study and the follow-up experiments were conducted following Swiss ethical guidelines and were authorised by the animal experimentation committee of the Canton de Vaud (VD3313). The CCl_4_-induced liver disease study was approved by the Ethics Review Committee of the Pohang Advanced Bio Convergence Center, Pohang, Republic of Korea (ABCC2022102).

## Results

### CHP counteracted diet-induced metabolic syndrome

**Fig. 1:**
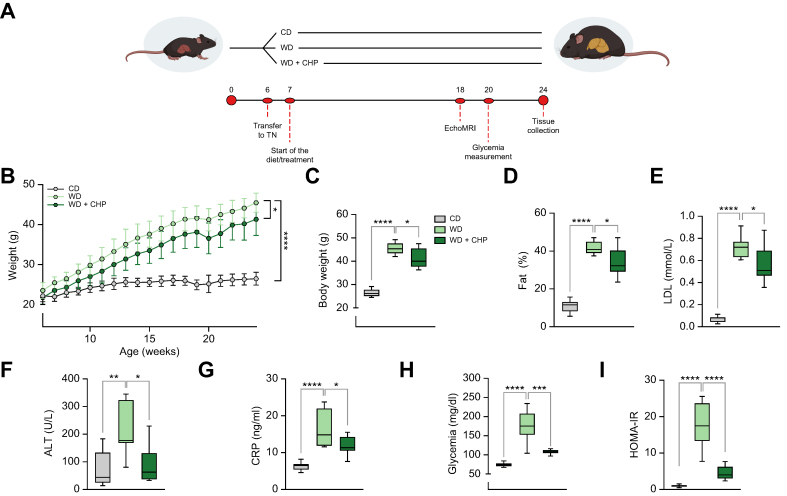
CHP prevents weight gain and fat accumulation and improves plasma biomarkers in the NAFLD mouse model. (A) Our NAFLD model was established through a combination of WD and thermoneutrality housing. Mice were housed in a thermoneutrality cabinet from 6 weeks of age. A specific diet (CD/WD) was introduced at Week 7. Treatment with CHP (20 mg/kg) was started at Week 7 and was given by gavage 3 times per week, for the whole duration of the study. Evaluation of fat mass by EchoMRI™ was performed at Week 18. Fasting glycaemia was measured at Week 20. Mice were euthanised at Week 24, after a 3-h fast. (B) Body weight increase over time. Weight was recorded once a week. (C) Body weight at Week 24. (D) Body fat percentage measured at Week 18 by MRI. (E–G) LDL-cholesterol (E), ALT (F), and CRP (G) levels in plasma. Blood was collected at the end of the experiment. (H) Glycaemia as measured after an overnight (14 h) fast. (I) HOMA-IR index for each group, calculated following the homeostatic model assessment (n = 6–8). Results represent the mean ± standard deviation (B) and the whiskers in boxplots represent the minimum to maximum range (C–I). Two-way ANOVA (B) and one-way ANOVA (C–I), followed by Dunnett’s multiple comparison test *vs.* WD group, were used for statistical analysis. ∗*p* <0.05; ∗∗*p <*0.01; ∗∗∗*p <*0.001; ∗∗∗∗*p <*0.0001. ALT, alanine transaminase; ANOVA, analysis of variance; CD, chow diet; CHP, cyclo(His-Pro); CRP, C-reactive peptide; HOMA-IR, homeostatic model assessment-insulin resistance; LDL, low-density lipoprotein; MRI, magnetic resonance imaging; NAFLD, non-alcoholic fatty liver disease; TN, thermoneutral housing; WD, western diet.

NAFLD was induced in 24-week-old male C57BL/6J mice by housing animals at TN (30°C–32 °C) and feeding with a WD, enriched in fat (21%) and sucrose (35%) (Fig. 1A). This combination of challenges enhances liver fat accumulation and reproduces most of the key pathological features of NAFLD.^41^^,^^42^ Mice fed with a matched control CD were used as an additional control group. CHP administration (20 mg/kg) slightly limited the weight gain caused by WD feeding (Fig. 1B and C) despite equal food intake and caloric consumption in the 2 groups (Fig. S1A, B). Notably, this was attributed to CHP effects on nutrient absorption (Fig. S1C). The reduced weight gain was reflected in a lower body fat mass, with an average reduction of 8% in fat mass after CHP administration, as quantified by MRI using the EchoMRI™ device (Fig. 1D). CHP attenuated the increase in plasma low-density lipoprotein (LDL)-cholesterol levels induced by the WD (Fig. 1E). Plasma alanine transaminase (ALT) was evaluated as a marker of liver dysfunction. Mice receiving WD had higher ALT levels, a phenomenon that was not observed in CHP-treated animals (Fig. 1F). WD increased C-reactive peptide (CRP) levels, suggesting a higher general inflammatory state elicited by the diet, inflammation that was attenuated by exposure to CHP (Fig. 1G). Insulin resistance plays a major role in NAFLD progression.^43^^,^^44^ Compared with CD feeding, WD-fed mice displayed hyperglycaemia, an effect that was remarkably blunted in CHP-treated animals that maintained glycaemia levels closer to physiological ranges (Fig. 1H). The homeostatic model assessment (HOMA)-insulin resistance (IR) index was calculated from fasting glucose and insulin levels (Fig. 1I), supporting the remarkable prevention of insulin resistance by CHP administration. In this diet-induced NAFLD model, CHP was able to counteract the harmful effects of WD, preventing the onset of metabolic syndrome.

### CHP prevented NAFLD progression, reducing hepatic steatosis, fibrosis, and inflammation

**Fig. 2:**
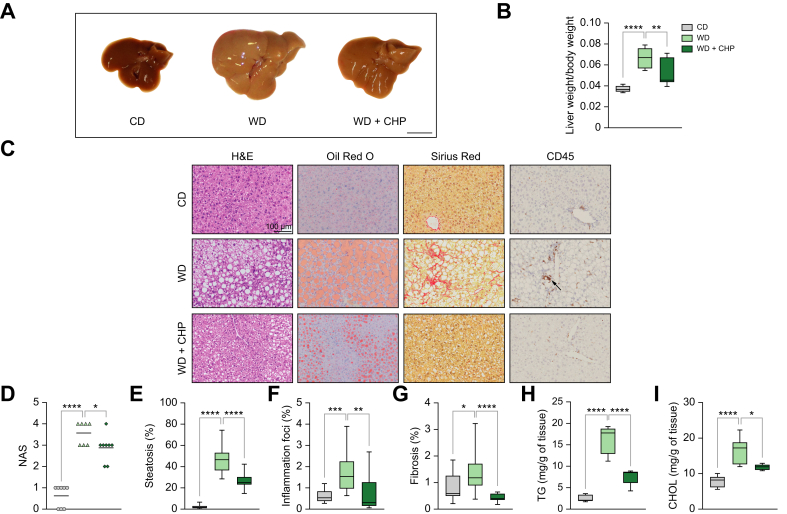
Liver steatosis, fibrosis and inflammation are prevented by CHP treatment. (A) Comparison of gross liver morphology in representative mice fed with CD/WD or WD supplemented with CHP. Scale bar = 1 cm. (B) Liver weight normalised on body weight, recorded at the end of the experiment (n = 7–8). (C) Representative images of liver sections stained with H&E, ORO, SR, and immunostained with CD45. (D) NAS scoring system, evaluating steatosis, lobular inflammation, and ballooning (n = 7). (E–G) Quantification of ORO (E), CD45^+^ (F), and SR (G) staining in histological images (n = 16). (H, I) Liver TG (H) and CHOL (I) content normalised by tissue weight (n = 5–6). Individual points are plotted and bar represents mean (D); whiskers in boxplots represent the minimum to maximum range (B, E–I). One-way ANOVA, followed by Dunnett’s multiple comparison test *vs.* WD group was used for statistical analysis (B, D–I). ∗*p <*0.05; ∗∗*p <*0.01; ∗∗∗*p <*0.001; ∗∗∗∗*p <*0.0001. ANOVA, analysis of variance; CD/WD, chow diet/western diet; CHOL, cholesterol; CHP, Cyclo(His-Pro); H&E, haematoxylin and eosin; NAFLD, non-alcoholic fatty liver disease; NAS, NAFLD activity score; ORO, Oil Red O; SR, Sirius Red; TG, triglycerides.

The size and colour of the livers in the WD group indicated extensive fat accumulation (Fig. 2A). In contrast, mice treated with CHP had smaller and darker livers, reflecting reduced steatosis (Fig. 2A, B). In blinded histopathological evaluation of tissue sections, CHP reduced the NAFLD activity score (NAS) (Fig. 2C, D). Haematoxylin and eosin (H&E), and Oil Red O (ORO) staining revealed the extensive macro- and micro-vesicular steatosis caused by WD feeding (Fig. 2E). Inflammation and fibrosis, important factors in the progression of NAFLD-NASH, were induced by long-term challenge with WD and TN, as indicated respectively, by increased CD45^+^ immune cell infiltration (Fig. 2F), and spread of fibrotic strands (Fig. 2G, Fig. S2). CHP administration attenuated hepatic steatosis while reducing fibrosis and inflammation (Fig. 2E–G). Protection from steatosis by CHP administration was further confirmed by assessing liver triglycerides (TG) and cholesterol (CHOL) levels (Fig. 2H, I). Taken together, our findings show that CHP hampered NAFLD progression in our model of WD/TN-challenged mice.

### Liver transcriptome analysis revealed the deleterious effects of WD on gene expression and their reversal by CHP

**Fig. 3:**
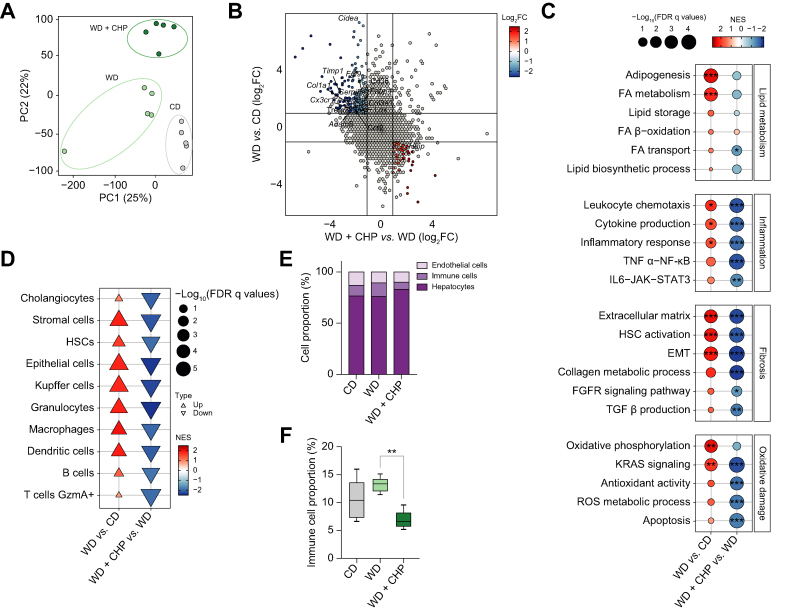
Transcriptomic signature of CHP in liver. (A) PCA plot of the RNA-seq profiles of the 3 groups. (B) Scatter plot shows the effects of WD compared to CD and the therapeutic effects of CHP on gene expression. The significant genes that show a reversal of the effect of WD compared with CHP treatment are highlighted in blue (upregulated upon WD) and red (upregulated upon treatment). Representative genes are annotated. (C) GSEA of disease (WD) and treatment (CHP) effects on gene expression. Gene sets are grouped into 4 categories: lipid metabolism, inflammation, fibrosis, and oxidative damage. ∗*Q <*0.05; ∗∗*Q <*0.01; ∗∗∗*Q <*0.001. (D) Cell type enrichment analysis of disease (WD) and treatment (CHP) effects on gene expression. (E) Single cell deconvolution results obtained from integrating the FACS scRNA data available under the GSE109774 accession and our bulk RNA-seq dataset, showing the relative depletion of immune cells upon CHP treatment. (F) Single-cell deconvolution estimated grouped cell proportions of immune cells in different conditions. Whiskers in boxplots represent the minimum to maximum range. One-way ANOVA, followed by Dunnett’s multiple comparison test *vs.* WD group was used for statistical analysis. ∗∗*p <*0.01. ANOVA, analysis of variance; CD, chow diet; CHP; cyclo(His-Pro); EMT, epithelial–mesenchymal transition; FA, fatty acids; FC, fold change; FDR, false discovery rate; FGFR, fibroblast growth factor receptor; GSEA, gene set enrichment analysis; HSC, hepatic stellate cells; NES, normalized enrichment score; NF-κB, nuclear factor kappa B; PCA, principal component analysis; ROS, reactive oxygen species; TGF-β, transforming growth factor beta; TNF-α, tumour necrosis factor alpha; WD, western diet.

Five livers from each treatment condition were randomly selected for RNA sequencing. In the principal component analysis (PCA), these samples were clustered according to diet and treatment (Fig. 3A). Gene expression was analysed to delineate the molecular signature of WD and CHP treatment, highlighting significantly up- and downregulated genes, particularly genes showing an opposite response upon exposure to WD and CHP treatment (Fig. S3A, B). The increased expression of transcripts involved in collagen synthesis (*e.g. Col1a1*, *Col3a1*, *Tipm1*) and lipid metabolism (*e.g. Fabp4*, *Cidea*) was reversed upon CHP treatment (Fig. 3B). CHP had a major influence on the transcriptome with 338 and 699 transcripts that were respectively significantly up- and downregulated compared to the WD condition (Fig. S3C). Gene set-enrichment analysis (GSEA) confirmed that lipid metabolism-related gene sets were upregulated upon the WD/TN challenge, as well as gene sets involved in inflammation (*e.g.* cytokine production and the nuclear factor kappa B [NF-κB] axis) and fibrosis (*e.g.* the extracellular matrix, hepatic stellate cells [HSC] activation, and collagen production) (Fig. 3C, Fig. S3D). According to histological observations, CHP downregulated the expression of many key genes involved in inflammation and fibrosis (Fig. S3D, E). Oxidative stress, apoptosis, and stress responses followed the same trend (Fig. 3C, Fig. S3D). A cell type enrichment analysis for 29 cell types showed that pro-fibrotic cells (including HSCs, stromal cells, and cholangiocytes) and immune cells (macrophages, B cells, and T cells) were enriched by WD and reduced by treatment with CHP (Fig. 3D). A single-cell deconvolution analysis, which quantified the proportion of different cell populations in the liver bulk RNA-seq data, highlighted the reduced infiltration of immune cells in liver tissue after CHP treatment (Fig. 3E, F). CHP did not appear to attenuate the macroscopic effect induced by the WD on the weight of extrahepatic organs (Fig. S4A). To verify the effects of CHP on extrahepatic organs, the kidneys and gastrocnemius muscles were randomly selected for RNA sequencing, as previously described for liver tissue samples. Approximately 20–55% of patients with NAFLD develop chronic kidney disease.^45^ An indication for kidney injury—indicated here by the urine albumin-to-creatinine ratio (ACR)—was observed in mice receiving WD and improved following CHP treatment (Fig. S4B). Furthermore, studies have also shown that NAFLD/NASH can also impact on muscle tissue.^46^^,^^47^ The effect of CHP in the transcriptome of kidney and muscle aligned with its gene profile in the liver, with both inflammation and fibrosis decreasing upon treatment (Fig. S4C), pointing in the direction of a conserved mechanism of action across tissues.

### CHP alleviates CCl_4_-induced liver damage in mice

**Fig. 4:**
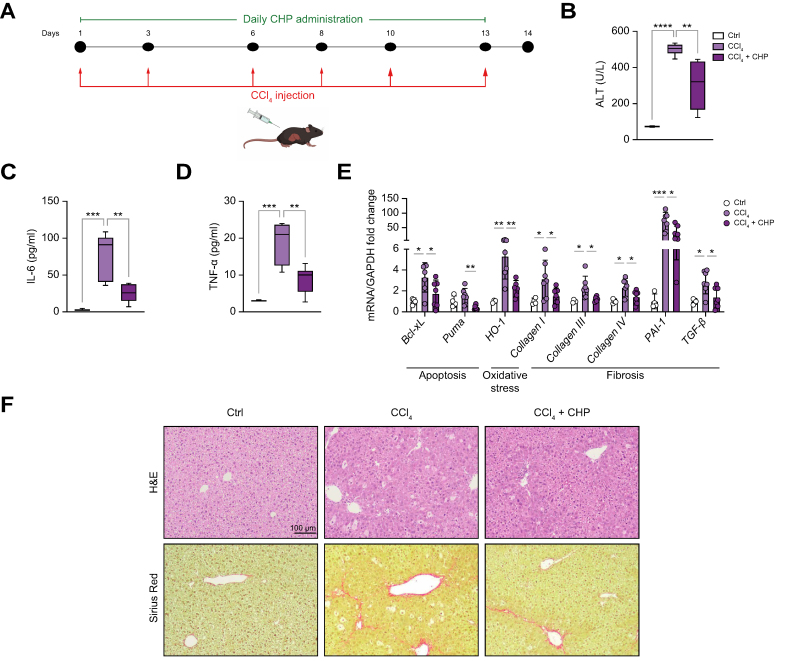
CHP prevents liver injury caused by 2 weeks of CCl_4_ injections. (A) Animal study flow. Mice received 6 injections of CCl_4_ over 13 days and were treated with CHP daily. Liver and plasma samples were collected at Day 14. (B–D) ALT (B), IL-6 (C), and TNF-α (D) plasma levels. Whiskers in boxplots represent the minimum to maximum range. (E) Liver expression of genes involved in apoptosis, oxidative stress, and inflammation. Error bars in bar plots represent the standard deviations. (F) Representative images of liver sections stained with H&E or SR. (n = 4 for CTRL, n = 6–7 for treatments). One-way ANOVA, followed by Dunnett’s multiple comparison test *vs.* CCl_4_ group was used for statistical analysis (B–E). ∗*p <*0.05; ∗∗*p <*0.01; ∗∗∗*p <*0.001; ∗∗∗∗*p <*0.0001. ALT, alanine transaminase; ANOVA, analysis of variance; Bcl-xL, B-cell lymphoma-extra large; CCl_4_, carbon tetrachloride; CHP, cyclo(His-Pro); CTRL, control; GAPDH, glyceraldehyde 3-phosphate dehydrogenase; H&E, haematoxylin and eosin; HO-1, heme oxygenase-1; IL-6, interleukin 6; PAI-1, plasminogen activator inhibitor 1; SR, Sirius Red; TGF-β, transforming growth factor beta; TNF-α, tumour necrosis factor alpha.

As a second model of early liver fibrosis, C57BL/6 mice received repeated low-dose (0.2 ml/kg) CCl_4_ injections over 2 weeks, together with daily CHP treatment (35 mg/kg by oral gavage) (Fig. 4A). CCl_4_ injection led to a significant increase in plasma ALT levels that was decreased by CHP treatment, suggesting prevention of liver damage (Fig. 4B). The observed reduction in plasma interleukin 6 (IL-6) and tumour necrosis factor alpha (TNF-α) levels confirmed the anti-inflammatory activity of CHP treatment (Fig. 4C, D). Analysis of liver gene expression showed how CHP downregulated apoptosis, oxidative stress, and fibrosis pathways (Fig. 4E). Hepatic H&E histological sections revealed the extensive cellular hypertrophy caused by CCl_4_ (Fig. 4F). Sirius Red (SR) staining uncovered early signs of fibrosis induced by chronic CCl_4_ injections (Fig. 4F). Treatment with CHP ameliorated the phenotype by reducing the presence of hypertrophic cells.

The protective effect of CHP against advanced CCl_4_-induced liver fibrosis was also evaluated in mice that underwent a longer protocol of CCl_4_ injections (Fig. S5A). Liver enzymes (ALT and aspartate transaminase [AST] levels) suggested reduced liver damage after CHP administration (Fig. S5B, C). This was further confirmed by histological analysis, which indicated that cellular hypertrophy and fibrosis, induced by prolonged CCl_4_ administration, were dampened by CHP (Fig. S5D).

**Fig. 5:**
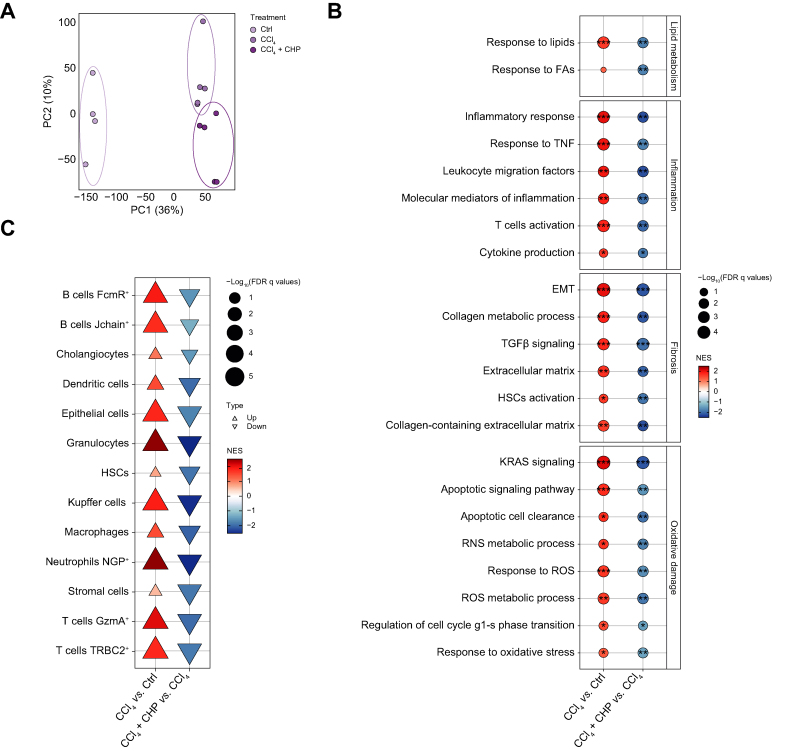
CHP prevents inflammation and fibrosis caused by CCl_4_ in liver. (A) PCA plot of the RNA-seq profiles. (B) GSEA of disease model (CCl_4_) and treatment (CHP) effects on gene expression. Gene sets are grouped in 4 categories: lipid metabolism, inflammation, fibrosis, and oxidative damage. ∗*Q <*0.05; ∗∗*Q <*0.01; ∗∗∗*Q <*0.001. (C) Cell type enrichment analysis of disease model (CCl_4_) and treatment (CHP) effects on gene expression. CCl_4_, carbon tetrachloride; CHP, cyclo(His-Pro); CTRL, control; FDR, false discovery rate; EMT, epithelial–mesenchymal transition; FA, fatty acids; GSEA, gene set enrichment analysis; HSC, hepatic stellate cells; NES, normalized enrichment score; NGP, neutrophilic granule protein; PCA, principal component analysis; RNS, reactive nitrogen species; ROS, reactive oxygen species; TGF-β, transforming growth factor beta; TNF, tumour necrosis factor.

Liver samples from the 2-week protocol (Fig. 4A) were further analysed by RNA sequencing to characterise the transcriptional effect of CHP. In the PCA, the samples were separated according to the experimental group (Fig. 5A). GSEA revealed that CCl_4_ strongly upregulated pathways involved in lipid metabolism and inflammation (Fig. 5B). According to the histological findings, profibrotic processes (*e.g.* HSC activation, epithelial–mesenchymal transition (EMT), extracellular matrix deposition) were upregulated by CCl_4_. Transcriptional evidence of CCl_4_ toxicity included the upregulation of apoptosis and reactive oxygen species (ROS)-mediated stress response genes (Fig. 5B). CHP potently attenuated pathways associated with inflammation and fibrosis, as well as apoptosis and oxidative damage, in this model of liver injury. Cell-type enrichment analysis confirmed a decrease in immune cells (*e.g.* B and T cells, macrophages, granulocytes, Kuppfer cells) and pro-fibrotic cells (*e.g.* HSCs, cholangiocytes) upon CHP treatment (Fig. 5C).

### A therapeutic intervention attenuated liver fibrosis and inflammation

**Fig. 6:**
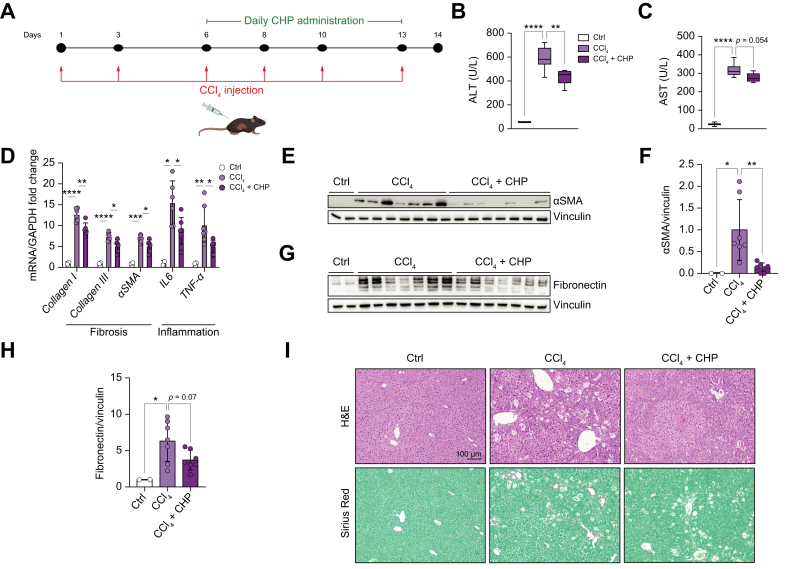
CHP recovers liver fibrosis and inflammation in a therapeutic protocol. (A) Animal study flow. Mice received 6 injections of CCl_4_ over 13 days and were treated with CHP daily starting after the third CCl_4_ injection. Liver and plasma were collected at Day 14. (B, C) ALT (B), and AST (C) plasma levels. Whiskers in boxplots represent the minimum to maximum range. (D) Liver expression of genes involved in fibrosis and inflammation. Error bars in bar plots represent the standard deviations. (E) Western blotting of αSMA expression; vinculin was used as loading control. (F) Quantification of αSMA signals from the western blotting in (E), normalised on vinculin. Error bars in bar plots represent the standard deviations. (G) Western blotting of fibronectin expression; vinculin was used as the loading control. (H) Quantification of fibronectin signal from the western blotting in (G), normalised on vinculin. Error bars in bar plots represent the standard deviations. (I) Representative images of liver sections stained with H&E or Sirius Red (0.1% Direct Red and 0.1% Fast Green FCF) (n = 2 for CTRL, n = 6–7 for other treatments). One-way ANOVA, followed by Dunnett’s multiple comparison test *vs.* CCl_4_ group was used for statistical analysis (B-D, F, H). ∗*p <*0.05; ∗∗*p <*0.01; ∗∗∗*p, <*0.001; ∗∗∗∗*p <*0.0001. ALT, alanine transaminase; AST, aspartate transaminase; CCl_4_, carbon tetrachloride; CHP, cyclo(His-Pro); CTRL, control; GAPDH, glyceraldehyde 3-phosphate dehydrogenase; IL-6, interleukin 6; αSMA, alpha smooth muscle actin; TNF-α, tumour necrosis factor alpha.

Our study demonstrated that preventive treatment with CHP can effectively reduce liver fibrosis and inflammation. To investigate whether CHP not only exerted a preventive effect, but could also have an impact upon therapeutic administration, a follow-up experiment was performed using a different model of CCl_4_-induced liver disease. C57BL/6 mice received a total of 6 CCl_4_ injections over a 2-week period, and daily treatment with CHP was started after the third injection (Fig. 6A). Plasma levels of ALT and AST were reduced by CHP treatment (Fig. 6B, C) and liver transcript levels showed a general reduction in fibrosis and inflammation (Fig. 6D). Alpha smooth muscle actin (αSMA) and fibronectin protein levels also confirmed the reduced deposition of extracellular matrix components (Fig. 6E-H). Liver histology supported the ability of CHP to reduce cellular hypertrophy and fibrosis (Fig. 6I, Table S1). Taken together, these results provide strong evidence that CHP can effectively counteract liver inflammation and fibrosis even in a therapeutic setting.

### CHP improved mitochondrial function in liver cells

**Fig. 7:**
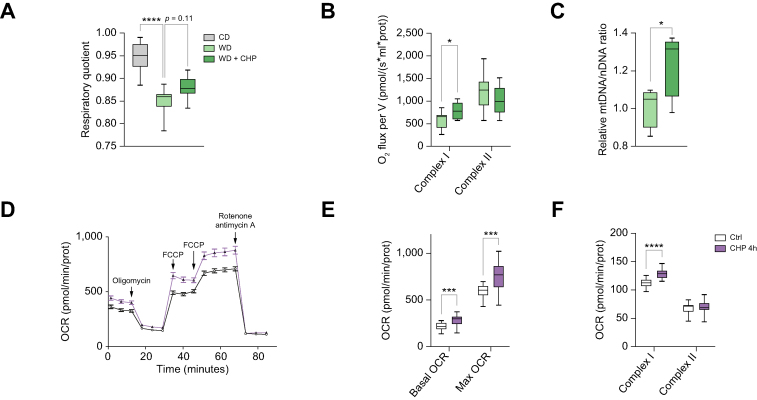
CHP improves mitochondrial function in hepatocytes. (A) Respiratory quotient during the night, as measured at age 18 weeks (n = 7–8). (B) Complex I and II activity, normalised on protein content, in mitochondria isolated from fresh liver tissue (n = 7–8). (C) Relative mtDNA levels in mice receiving a WD, calculated as 16S mitochondrial gene expression normalised against hexokinase gene (n = 5). (D) Oxygen consumption of AML12 cells treated with 50 nM CHP for 4 h, when measuring respiration in basal, maximal, or leak state. OCR is normalised by protein content (n = 22–24). (E) Basal and maximal respiration as extracted from panel D. (F) Complex I and II activity in AML12 cells treated with 50 nM CHP (n = 23). One-way ANOVA, followed by Dunnett’s multiple comparison test *vs.* WD group (A) or unpaired *t*-test (B, C, E, F) were used for statistical analysis. ∗*p <*0.05; ∗∗∗*p <*0.001; ∗∗∗∗*p <*0.0001. AML12, alpha mouse liver 12; CHP, cyclo(His-Pro); FCCP, carbonyl cyanide-p-trifluoromethoxyphenylhydrazone; mtDNA, mitochondrial DNA; OCR, oxygen consumption rate; WD, western diet.

Correct mitochondrial function in hepatocytes is fundamental to maintain energy homeostasis and hepatic metabolic functions. The respiratory quotient of mice, as measured in metabolic cages, gives an indication of the ratio of carbohydrate to fat oxidation and has been previously correlated with cirrhosis and NAFLD progression.^48^^,^^49^ The respiratory quotient showed a marked decrease upon WD feeding, which trended towards recovery following exposure to CHP (Fig. 7A). CHP positively acted on mitochondria, by increasing complex I activity in isolated mitochondria from liver tissue (Fig. 7B) and by increasing mitochondrial content in the livers of mice challenged with TN/WD (Fig. 7C). To study the effects of CHP on mitochondrial function, alpha mouse liver 12 cells (AML-12) were treated with CHP for 4 h and then cellular respiration was analysed (Fig. 7D). Both basal and maximal respiration were increased by CHP (Fig. 7E), and the increase in basal respiration was specifically due to an increase in complex I activity (Fig. 7F), as observed in liver mitochondria (Fig. 7B). Overall, these data suggest that CHP enhanced mitochondrial number and activity in the liver of our NAFLD model, a finding supported by *in vitro* data on mouse hepatocytes.

### Inhibition of ERK signalling was an early effect of CHP treatment

**Fig. 8:**
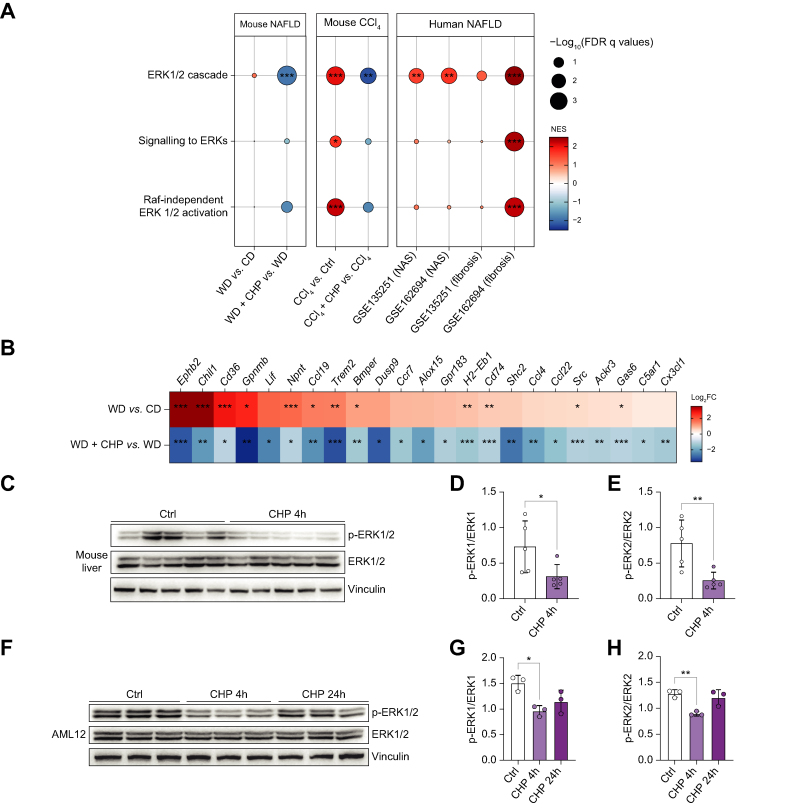
CHP triggers an early downregulation of ERK signalling. (A) GSEA of the effects of disease (WD/CCl_4_) and treatment (CHP) on gene expression in the mouse liver in this study (left panels), as well as that of the effects of disease on humans (NAS ≥4 *vs*. NAS <4 and fibrosis ≥3 *vs.* fibrosis <3) for ERK-related gene sets. (B) Heatmap shows the effects of WD and CHP on the expression of genes involved in ERK1/2 signalling. ∗*p <*0.05; ∗∗*p <*0.01; ∗∗∗*p <*0.001 (adjusted *p* values). (C) Western blotting of phosphorylated and total ERK1/2 in the liver of mice treated with 20 mg/kg CHP for 4 h. Vinculin was used as the loading control. (D, E) Quantification of phosphorylated ERK1 (D) and ERK2 (E) signals from the western blotting in (C), normalised by total ERK1/2. Unpaired *t*-test was used for statistical analysis. (F) Western blotting of phosphorylated and total ERK1/2 in the AML12 cell line, treated with 100 nM CHP for 4 or 24 h. Vinculin was used as the loading control. (G, H) Quantification of phosphorylated ERK1 (G) and ERK2 (H) signals from the western blotting in (F), normalised by total ERK1/2. One-way ANOVA, followed by Dunnett’s multiple comparison test *vs.* CTRL group was used for statistical analysis. Error bars in bar plots represent the standard deviation (D, E, G, H). ∗*p <*0.05; ∗∗*p <*0.01. AML12, alpha mouse liver 12; ANOVA, analysis of variance; CCl_4_, carbon tetrachloride; CHP, cyclo(His-Pro); CTRL, control; ERK, extracellular signal-regulated kinase; FC, fold change; FDR, false discovery rate; GSEA, gene set-enrichment analysis; NAFLD, non-alcoholic fatty liver disease; NAS, NAFLD activity score; NES, normalized enrichment score; WD, western diet.

Among the enriched pathways that emerged from the GSEA, the ERK signalling cascade was significantly downregulated by CHP, both in the WD/TN NAFLD and in the CCl_4_-induced liver disease models (Fig. 8A left panels; S6A). Of note, this pathway was also over-represented in 2 RNAseq data sets collected from patients with NASH, when samples from patients with advanced NASH—with high NAS and fibrosis—are compared to earlier stages (Fig. 8A, right panel). Several upstream and downstream genes of the ERK pathway were differentially regulated in RNAseq data from the NAFLD study (Fig. 8B). Downregulation of the ERK signalling cascade was not limited to the liver tissue (Fig. S4C).

Protein expression of the phosphorylated (active) form of ERK1/2 (p-ERK1/2) in the livers at the end of the chronic feeding period of WD did not show any differences after CHP treatment (Fig. S6B). Therefore, a short-term experiment was performed in healthy male C57BL/6J mice, to monitor ERK1/2 phosphorylation at different time points after CHP treatment. Liver levels of p-ERK1/2 were already reduced 4 h after CHP treatment (Fig. 8C–E). Baseline p-ERK1/2 levels were restored after 24 h (Fig. S6C). To confirm this observation *in vitro*, AML-12 cells were treated with CHP for 4 or 24 h and protein levels were analysed by western blotting (Fig. 8F-H). The p-ERK1/2 levels were robustly reduced 4 h after CHP treatment, and at 24 h returned closer to baseline. Overall, these data indicated that CHP inhibits ERK1/2 in the short term, by reducing its phosphorylation and downregulating its downstream pathways.

## Discussion

In this study, we assessed the effects of CHP on the pathogenesis of NAFLD in a mouse model in which animals were challenged with WD feeding and TN housing.^41^^,^^42^ Under WD, mice constantly gained weight over time, and several plasma parameters associated with increasing fat accumulation, including levels of LDL-cholesterol, glycaemia, and liver enzymes. Mice fed with WD developed enlarged livers, marked by broad steatosis, inflammation, and fibrosis, thus reproducing the main features of the NAFLD/NASH transition. CHP administration restored glucose homeostasis and prevented hepatic fat accumulation as seen by histological evaluation. Steatosis, inflammation, and fibrosis were all partially prevented by treatment, and the pathological evaluation indicated that CHP attenuated the progression of NASH.

As highlighted by our transcriptome analysis, CHP inhibited genes related to lipid metabolism, explaining the overall reduced fat accumulation observed in our murine models. Furthermore, CHP robustly downregulated pro-inflammatory pathways and reduced immune cell infiltration in the liver, supporting the histological findings. Reducing inflammation in NAFLD is crucial in breaking the vicious circle towards liver fibrosis^50^^,^^51^ and eventually toward hepatocellular carcinoma.^52^

CHP also proved to be an effective preventive strategy in a second mouse model of liver disease, induced by repeated low-dose CCl_4_ injections. The hepatotoxicity of CCl_4_ was manifested in histology by extensive cellular hypertrophy and fibrosis. At the gene expression level, the effects of CHP were similar to those observed in the WD/TN model and also confirmed the attenuation of apoptosis and oxidative stress pathways. These 2 animal models of liver disease—induced by WD/TN or CCl_4_—have different mechanisms of action leading to inflammation and fibrosis, nonetheless CHP proved to exert preventive effects in both settings (Fig. 1, Fig. 4A, Fig. S5A). To provide support for a therapeutic effect of CHP, a follow-up study tested the effects of CHP using a CCl_4_ mouse model in which treatments were started at a later time point (Fig. 6A). Also in this setting, when CHP was administered as a treatment after symptoms of CCl_4_-induced liver damage were evident, it was able to attenuate and blunt disease progression, providing a strong indication that CHP could be a candidate drug to manage NAFLD/NASH in the clinical setting.

The liver is a highly metabolic organ involved in the metabolism of all macronutrients. Several studies have underscored the link between mitochondrial function and NAFLD, and mitochondria have become an interesting therapeutic target.[53], [54], [55], [56] Indeed, also in our studies, we observed that mitochondrial function was impaired by WD/TN challenge. Mice treated with CHP showed an improved mitochondrial phenotype *in vivo*, stimulating us to further study the effects of CHP on mitochondrial respiration *in vitro*. In mouse hepatocytes, CHP also increased cellular respiration, particularly the activity of complex I of the mitochondrial oxidative phosphorylation system. CHP effects on mitochondria were previously unknown, and thus, warrant further characterisation, not only in hepatocytes but also in other liver cell types. We speculate that the discrete changes in mitochondrial metabolism that we observed might not have a visible impact on overall energy expenditure, but may still play a role in maintaining hepatocyte function and glucose and lipid metabolism, contributing to the final beneficial phenotypic changes after CHP treatment.

Two human datasets collected from patients with NASH showed that ERK signalling is activated in patients with increasing severity of NAS and fibrosis stage. The ERK pathway has been associated with the development of NASH, as its activation plays a role in driving inflammation and fibrosis.^57^^,^^58^ Inhibition of ERK1 has been associated with decreased proliferation of hepatic stellate cells, attenuated fibrosis, and improved lipid homeostasis.^59^^,^^60^ ERK2 activity affects hepatocyte proliferation, playing also a role in inflammation and fibrosis upon liver injury.^61^^,^^62^ Consistent with these studies, ERK signalling pathways were found to be upregulated in our liver transcriptome data, and CHP treatment downregulated ERK downstream genes. Our study showed that the direct effect of CHP on p-ERK1/2 levels in the liver was transient and could be detected after only 4 h, returning to baseline levels 24 h after CHP treatment. However, the effects on the downstream genes persisted longer, as shown in the transcriptome analysis. The same transient reduction of p-ERK1/2 levels with a nadir at 4 h was also observed *in vitro* in cultured AML12 hepatocytes. The inhibition of ERK activation after CHP was consistent in both *in vivo* and *in vitro* models, demonstrating for the first time a direct action of CHP on the ERK1/2 signalling pathway.

CHP was well tolerated in our long-term 17-week study in mice subjected to WD, as well as in a 39-week formal toxicology study performed in Beagle dogs (unpublished data). Furthermore, initial clinical studies have reported no safety concerns with CHP (NCT00878605; NCT02784275; NCT03560271). Its impeccable safety profile, together with the remarkable anti-inflammatory and antifibrotic effects of CHP in our study, point to CHP as a potential new strategy to prevent and manage NAFLD/NASH. Furthermore, our data implicate the downregulation of the ERK pathway as an early event after CHP treatment, although further work will be required to determine whether a direct or indirect inhibition by CHP is responsible for ERK regulation.

Overall, CHP appears to be promising candidate drug for the management of the transition from NAFLD to NASH. Furthermore, our data strongly suggest exploring the impact of CHP on additional diseases characterised by inflammation and fibrosis, such as chronic kidney disease or idiopathic pulmonary fibrosis.

## Financial support

The work in the JA laboratory was supported by grants from the 10.13039/501100001703École Polytechnique Fédérale de Lausanne (EPFL), the 10.13039/501100000781European Research Council (ERC-AdG-787702), the Swiss National Science Foundation (SNSF 31003A_179435), the Global Research Laboratory (GRL) 10.13039/501100003725National Research Foundation of Korea (NRF 2017K1A1A2013124), and NovMetaPharma. ADM was funded by a European Innovative Training Networks H2020-MSCA-ITN-2018 (Healthage - 812830); QW was supported by the 10.13039/100004410European Molecular Biology Organization (EMBO) postdoctoral fellowship (ALTF 111-2021); XL was supported by the 10.13039/501100004543China Scholarship Council (201906050019).

## Authors’ contributions

The study was conceived by ADM, AM, HYJ, and JA. ADM, SB, and QW performed the *in vivo* experiments and the analysis of the experimental results. RNA sequencing analysis was carried out by XL and KS. In vitro experiments were performed by ADM, JJ, DL, OP, and QW. AM supported the organisation of the animal study. ADM and JA wrote the manuscript with contributions from all co-authors. JA and HYJ supervised the study and secured funding.

## Data availability statement

The data supporting the findings are available upon request to the corresponding author (JA). RNA-seq data are available under the GEO numbers GSE200750, GSE216366 and GSE230745. Methods, materials, and resources are included in the Materials and methods or Supplementary Methods.

## Conflicts of interest

This work was funded in part by a grant from NovMetaPharma. DL, JJ, SB, and OP are employees of NovMetaPharma. HYJ is a board member of NovMetaPharma. JA is a founder and/or consultant to MitoBridge/Astellas, Metro Biotech, Amazentis, Vandria, Orso Bio, and NovMetaPharma. All other authors declare that they have no competing interests.

Please refer to the accompanying ICMJE disclosure forms for further details.
