# Supplementary material for: Cyclo(His-Pro): A further step in the management of steatohepatitis
Source: JHEP Rep. 2023 Jun 10;5(9):100815. doi: 10.1016/j.jhepr.2023.100815 (PMC10432811; doi:10.1016/j.jhepr.2023.100815)
Supplement: Multimedia component 1 [file mmc1.pdf]

Cyclo (His-Pro): a further step in the management of steatohepatitis

Alessia De Masi[1], Xiaoxu Li[1], Dohyun Lee[2], Jongsu Jeon[2], Qi Wang[1], Seoyeong Baek[2], Onyu Park[2], Adrienne Mottis[1], Keno Strotjohann[1], Alexis Rapin[1], Hoe-Yune Jung[2,3,*], Johan Auwerx[1,*].

[1] Laboratory of Integrative Systems Physiology, Institute of Bioengineering, École Polytechnique Fédérale de Lausanne, Lausanne 1015, Switzerland

[2] R&D Center, NovMetaPharma Co., Ltd., Pohang, 37668, South Korea

[3] School of Interdisciplinary Bioscience and Bioengineering, Pohang University of Science and Technology (POSTECH), Pohang, 37673, South Korea

* Corresponding authors:

Johan Auwerx - Address: Laboratory of Integrative and Systems Physiology, Ecole Polytechnique Fédérale de Lausanne, CH-1015 Lausanne, Switzerland. Tel.: +41 216939522. E-mail address: admin.auwerx@epfl.ch

Hoe-Yune Jung – Address: R&D Center, NovMetaPharma Co., Ltd., Pohang, 37668, South Korea. Tel.: +82 (0)54 223 2893. E-mail address: elijah98@novmeta.com

Table of contents

Supplementary methods 3
Supplementary figures 7
Supplementary tables 12
Supplementary references 14

Supplementary methods

Plasma biochemistry. For plasmatic biochemistry, blood samples were collected by intracardiac puncture under anesthesia, plasma was separated and stored at -80°C. Plasma parameters were measured using Dimension®Xpand Plus (Siemens Healthcare Diagnostics AG). Liver enzymes were assayed to assess a possible liver damage state. LDL-cholesterol levels were measured. The biochemical tests were performed according to the manufacturer kit for each parameter: ALT, AST, LDL (Siemens Healthcare). CRP concentration was measured using Mouse CRP ELISA Kit (Crystal Chem). Plasma IL-6 and TNFα were measured with ELISA Kits (Invitrogen).

Urine biochemistry. Urine was collected the day before euthanasia and stored at -80°C. Creatinine and micro-albumin were measured using AU480 Clinical Chemistry System (Beckman Coulter) following manufacturer's protocols.

Liver characterization. Macroscopic pictures of livers were taken after PBS (Gibco) perfusion. Liver TG levels were measured with TRIGL kit (c111, Cobas, Roche). CHOL levels were measured with CHOL2 kit (c111, Cobas, Roche). To measure the activity of mitochondrial complexes in mouse liver, mitochondria were isolated from fresh whole liver tissue as previously described [1]. Pellets of mitochondrial were quantified for proteins, equalized and resuspended in MiR05 (Oroboros Instruments). Oxygen consumption rate (OCR) was assessed by high-resolution respirometry (Oxygraph 2k, Oroboros Instruments) according to the manufacturer's protocol. Compounds were added directly into the 2 ml chamber: pyruvate (5 mM), malate (2 mM), glutamate (10 mM), ADP+Mg^{2+} (1.25 mM) for complex I; succinate (10 mM) for complex II; inhibitors for complex I (rotenone 0.5 μM) and complex II (antimycin A, 2.5 μM); all compounds are from Sigma. Mitochondrial content was measured by the relative mtDNA/nDNA ratio as previously described [2].

Histology. For histological analysis, liver samples were taken from the same lobe of each animal. 4 µm paraffin sections were processed with standard H&E staining to assess the general morphology, and Sirius red F3B (SR) or Direct Red + Fast Green FCF as counterstaining (Sigma) to highlight collagen fibers. Detection of CD45 positive immune cells was performed with ChromoMap DAB kit (Roche Diagnostics). 8 µm cryosections sections were processed with standard ORO protocol to detect lipids. Images were taken with an Olympus Slide Scanner VS120 L100 at 40x magnification. Digital slides were analyzed using QuPath software [3]. Stained liver tissue was quantified taking 4 random 8x fields on each slide, using 4 slides per experimental group; signal was quantified using ImageJ-Fiji software [4]. The histopathological assessment was performed in a blinded fashion by a board-certified veterinary pathologist (DECVP).

RNA-seq. The differentially expressed genes (DEGs) are defined by a Benjamini–Hochberg adjusted *P* value lower than 0.05 and an absolute $\log_2$[fold change] value higher than 1. GSEA was performed from fold-change sorted genes using clusterProfiler R package (version 3.10.1) [5], using gene sets retrieved with the msigdbr R package (version 7.2.1) [6]–[8]. 11 additional custom gene sets related to HSC were obtained from GO terms (2000490, 2000491) and literature [9]–[13] (Table S2). The gene sets with absolute normalized enrichment score (NES) higher than 1 and false discovery rate (qValue) lower than 0.05 are identified as significantly enriched gene sets.

The gene markers for 29 cell types in liver were retrieved from the supplementary material of two single cell RNA sequencing (scRNA-seq) studies and calculated using ClusterProfiler [5], [14], [15].

Cell type deconvolution analysis was performed with the using MuSiC R package (version 0.2.0), with the liver scRNA-seq dataset [16] as a reference. Cell types were summarized

into three categories: endothelial cells (endothelial cell of hepatic sinusoid), hepatocytes and immune cells (B cell, Kupffer cell, Natural Killer cell).

The effect of increasing severity of NAS and fibrosis stages was measured in two human NAFLD datasets (GSE135251 [17] and GSE162694 [18]). We compared NAS 4-8 versus NAS 0-3 and Fibrosis 3-4 versus Fibrosis 0-3 using the limma R package (version 3.38.3) with sex as a covariate, then performed GSEA using the clusterProfiler package.

RNA extraction, cDNA synthesis, and Real-time PCR. Total RNA was extracted from the liver using NucleoZOL reagent (Macherey-Nagel). 1µg of total RNA was used for cDNA synthesis using iScript cDNA synthesis kit (Bio-rad). Real-time qPCR (RT-qPCR) was performed using IQ SYBR® Green Supermix (Bio-Rad). The gene expression level was normalized to *Gapdh* gene. Specific primer pairs are listed in table S3.

Cellular respiration. Cellular respiration was assessed in AML12 cells treated with 50 nM CHP for 4 hours. Oxygen consumption rate was measured with the Seahorse XF96 instrument (Agilent), according to the manufacturer's protocol. Compounds were injected in the wells during measurement to assess basal and maximal respiration: oligomycin (0.1 µM), carbonyl cyanide-p-trifluoromethoxyphenylhydrazone (FCCP, 1 µM), rotenone (1 µM), antimycin A (1 µM). All compounds are from Sigma. To measure the specific activity of mitochondrial complexes, cells were permeabilized with 7.5 µg/ml digitonin and standard protocol from manufacturer (Agilent) was followed.

Western blot. Proteins were extracted in RIPA buffer, and Laemmi buffer was added for loading. Samples were loaded on 8% acrylamide sodium dodecyl sulfate-polyacrylamide gel (SDS-PAGE), then proteins were transferred onto polyvinylidene fluoride (PVDF) membranes (Immobilon-P PVDF Membrane, Millipore). Membranes were blocked with 5% skim milk-TBST, and incubated with primary antibodies overnight. Secondary antibody detection reactions were developed by enhanced chemiluminescence (SuperSignal West

Pico PLUS Chemiluminescent Substrate, Thermo Scientific) and imaged using the Fusion FX imaging system (Vilber). Quantification was performed using ImageJ software.

Antibodies. For histology, CD45 antibody (rat α-CD45, Thermo Fisher) was used. For western blotting, the following primary antibodies were used: phospho-ERK1/2 (rabbit α-phospho-p44/42 MAPK, Cell signaling), ERK1/2 (rabbit α-p44/42 MAPK, Cell signaling), Vinculin (rabbit recombinant α-vinculin, Abcam), αSMA (rabbit α-αSMA, Cell signaling), fibronectin (rabbit α-fibronectin, abcam).

Figures. BioRender was used to draw the animal studies outline (Fig. 1A, 4A, 6A, S5A) and the graphical abstract. Time-course, boxplots and barplots were created with GraphPad Prism 9.5.1. ImageJ software was used to prepare the western blot images. Adobe Illustrator 26.0.1 was used to assemble figure panels.

Supplementary figures

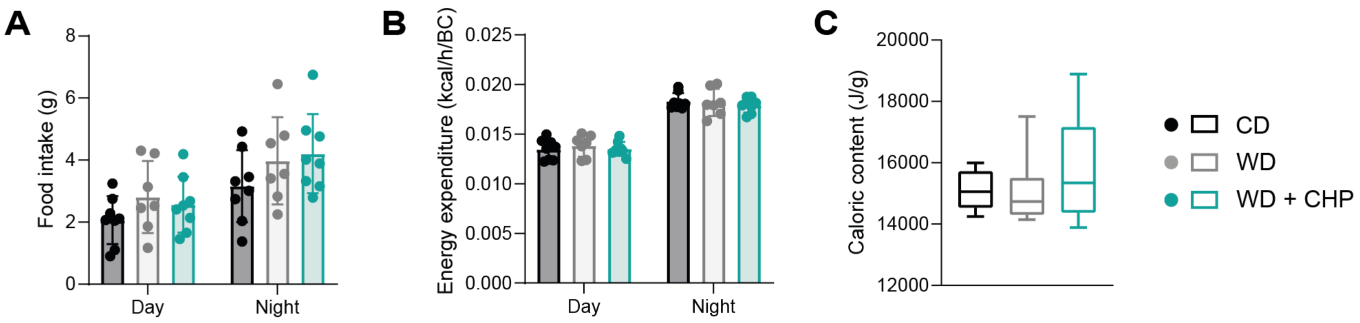

Fig. S1. CHP effect on metabolism. (A) Total food intake during day and night time. (B) Average hourly energy expenditure normalized on body composition (BC), considering both lean and fat mass. n=7-8. Results represent the mean ± standard deviation. (C) Fecal calorie content measured on feces collected over a 24-hour period, after 8 weeks of CD or WD and treatment with CHP. Whiskers in boxplots represent the min to max range. n=9-10.

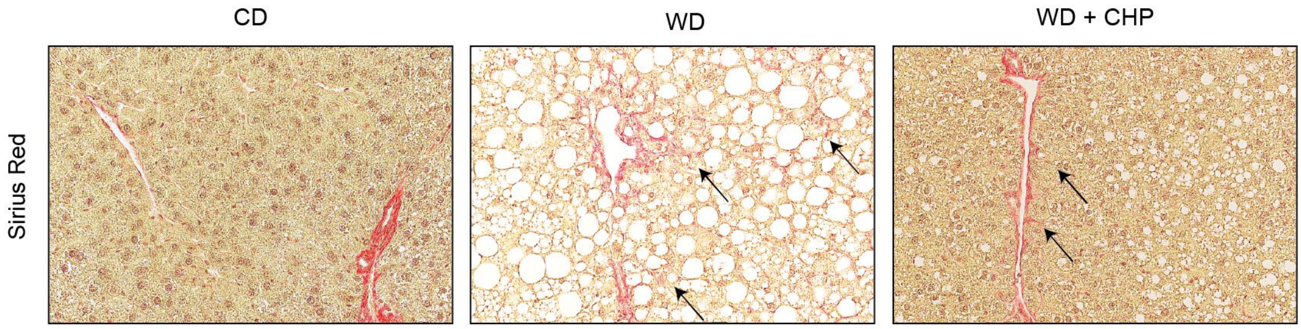

Fig. S2. CHP reduced the extension of fibrosis in mice fed with WD. Representative images of liver sections stained Sirius Red. Arrows indicate the extension of the collagen strands from the vessel to the periportal and midzonal area.

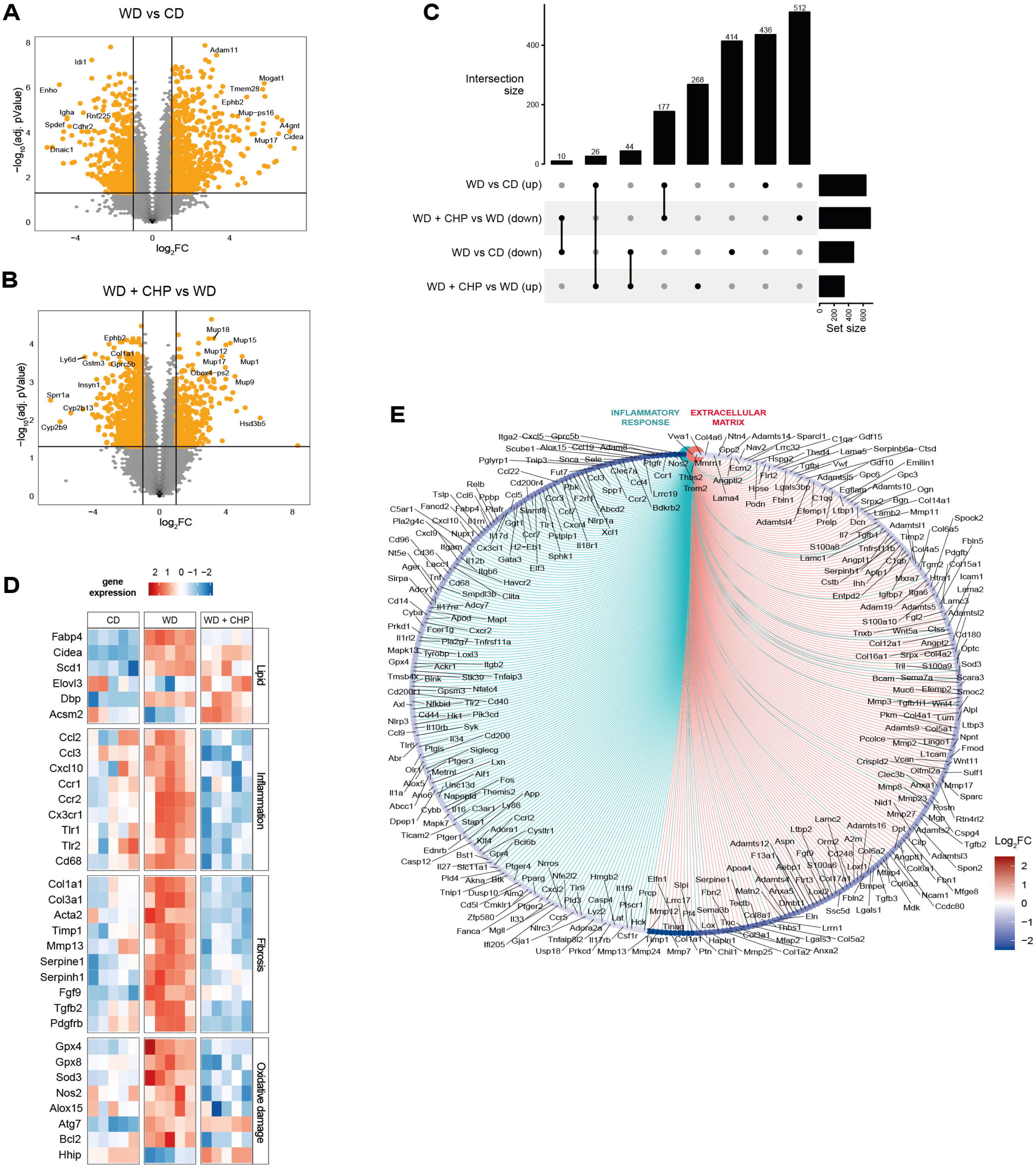

Fig. S3. Transcriptomic signatures of WD/TN and CHP. (A) Volcano plot showing the effect of WD on gene expression compared to the baseline condition (CD). (B) Volcano plot showing the effect of WD + CHP on gene expression compared to WD. The differentially expressed genes (|log2FC| >1 and adjusted *P*-value < 0.05) are highlighted in orange (A, B). (C) Upset Plot showing the exclusive intersections for the significantly differential expressed genes between comparisons. (D) Heatmap showing the effects of WD and CHP for given genes, grouped following the same four categories as in Fig.3C. (E) Gene-concept network

(cnet) plot showing the core enriched genes in inflammation and extracellular matrix gene sets for the effect of CHP.

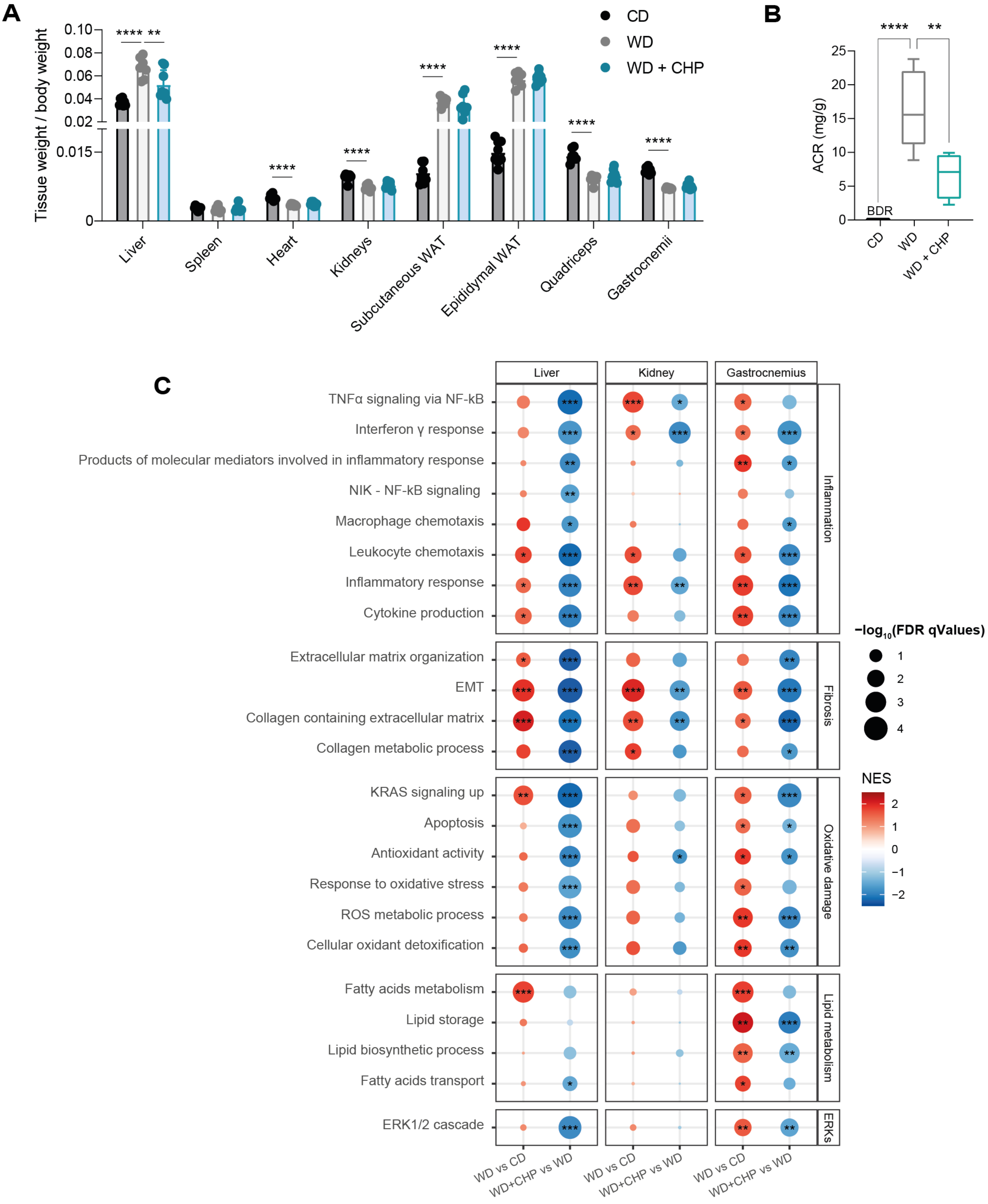

Fig. S4. Effects of WD feeding and CHP treatment on extrahepatic organs. (A) Comparison of organ size between the three experimental groups. n=7-8. (B) Albumin to creatinine ratio (ACR) in urine, expressed as mg of albumin to g of creatinine. BDR: below

detection range. n=5-7. One-way ANOVA, followed by Dunnett's multiple comparison test versus WD group was used for statistical analysis. Error bars in barplots represent the standard deviation; whiskers in boxplots represent min to max range. *P* values are indicated as follows: ** $P<0.01$; **** $P<0.0001$. (C) Gene set enrichment analysis of disease (WD) and treatment (CHP) effects on gene expression, analyzed across three tissues (liver, kidney, gastrocnemius). Gene sets are grouped in five categories: Inflammation, Fibrosis, Oxidative damage, Lipid metabolism, ERKs. *Q* values are indicated as follows: * $Q<0.05$; ** $Q<0.01$; *** $Q<0.001$.

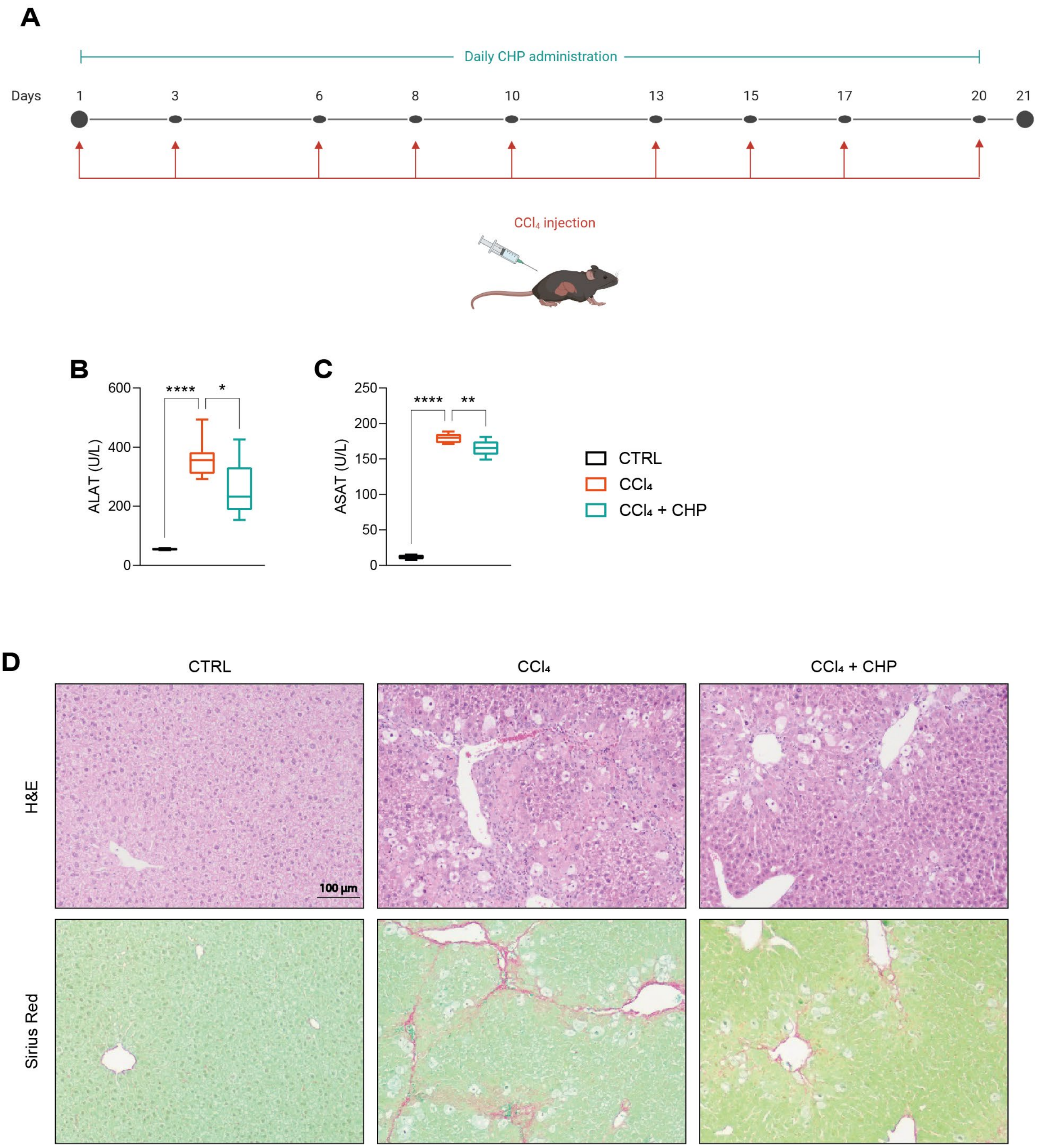

Fig. S5. CHP attenuated fibrosis and inflammation in a CCl_4-induced liver injury model. (A) Animal study outline. Mice received 9 injections of CCl_4 over 20 days, and were

treated with CHP daily. Liver and plasma were collected at day 21. (B-C) ALAT (B) and ASAT (C) plasma levels. Whiskers in boxplots represent the min to max range. (D) Representative images of liver sections stained with H&E or Sirius Red. n=4-7. One-way ANOVA, followed by Dunnett's multiple comparison test versus CCl_4 group was used for statistical analysis (B, C). *P* values are indicated as follows: * *P*<0.05; ** *P*<0.01; **** *P*<0.0001.

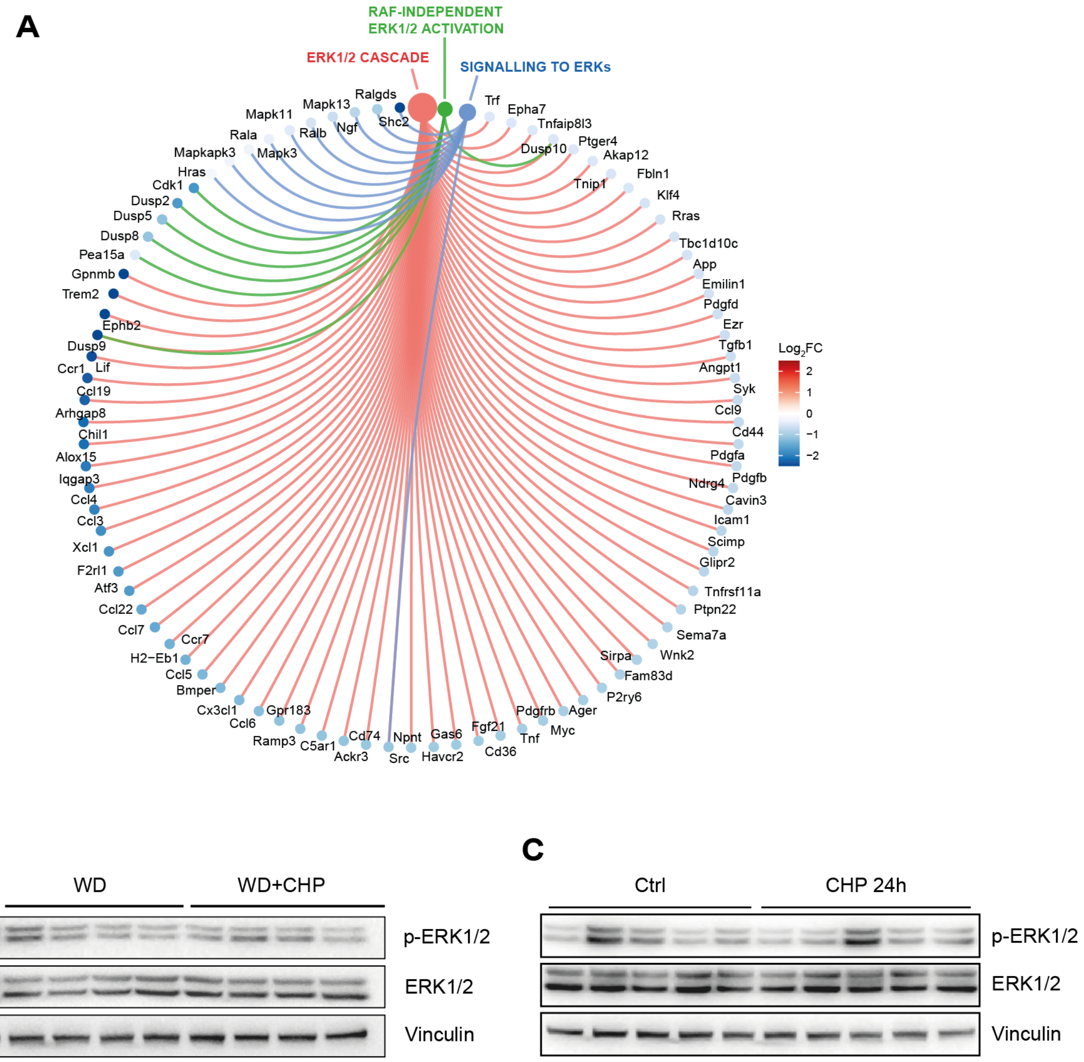

Fig. S6. ERK signaling in response to CHP treatment. (A) Gene-concept network (cnet) plot shows the core enriched genes in ERK signaling gene sets for the effect of CHP, in the WD/TN NAFLD model. (B) Western blot of phosphorylated and total ERK 1/2 in the liver of mice from the NASH study. Vinculin was used as loading control. (C) Western blot of phosphorylated and total ERK 1/2 in the liver of mice treated with 20 mg/kg CHP for 24 hours. Vinculin was used as loading control.

Supplementary tables

Table S1. Incidence and severity of remarkable histopathological findings in liver of mice subjected to CCl_4 injections.

		CTRL	CCl_4	CCl_4+CHP
**Number of animals**		2	7	7
**HE stain**				
Hyperplasia, oval cells	+	0	7	7
Hypertrophy, hepatocellular, centrilobular	++	0	7	7
Necrosis, hepatocellular, centrilobular	+<br>++<br>+++	0<br>0<br>0	0<br>0<br>7	4<br>3<br>0
**SR stain**				
Increased stain, centrilobular	+	0	7	0

Grade: + minimal, ++ mild, +++ moderate, ++++ marked

Table S2. 11 custom gene sets related to HSCs.

Gene set	Gene name	Ref.
HSC_activation_marker	Timp1	doi: 10.1038/s41598-019-39112-6
	Spp1	
	Mmp3	
	Gas6	
	Acta2	
	Col1a1	
	Col3a1	
	Col5a2	
	S100a6	doi: 10.3390/cells8050503
	saa3	
	Lox	doi: 10.1038/s41598-019-39112-6
	Lrat	
	Mfap4	doi: 10.1002/hep.31215
	Col1a2	
	Dpt	
HSC_proliferation	Egr1	doi: 10.1038/s41598-019-39112-6
	Ccnd1	
	Top2a	
	Cenpe	
	Rrm2	
Positive_regulation_of_HSC_activation_1	Lep	GO:2000491
	Dgat1	
	Acta2	

	Fgfr1	
	Pdgfrb	
	Pdgfb	
	Rps6ka1	
	Myocd	
Positive_regulation_of_HSC_activation_2	cdh11	doi: 10.1371/journal.pone.0233702
	cthrc1	
	fmod	
	prrx1	
	mfap4	
	pcdh15	
	ptprt	
	hpca	
Negative_regulation_of_HSC_activation	Gclc	GO:2000490
	Rian	
	Cygb	
	Gsk3b	
	Hhip	doi: 10.1371/journal.pone.0233702
Resting_HSC_marker	Fcna	doi: 10.3390/cells8050503
	Angptl6	
	Colec11	
	Tmem56	
	Plvap	
	Pth1a	
MFB1	Acta2	
	Tagln	
	Col1a1	
	Col6a3	
	Tpm1	
MFB2	Slpi	
	Saa3	
	c3	
	dmkn	
	cd74	
MFB3	Jund	
	Fosb	
	Egr1	
	Klf2	
MFB4	Mgp	
	Fbln	
	Meg3	
	Gas6	
	Hp	
MFB_1_TO_4	Acta2	
	Tagln	
	Col1a1	
	Col6a3	

	Tpm1	
	Slpi	
	Saa3	
	c3	
	dmkn	
	cd74	
	Jund	
	Fosb	
	Egr1	
	Klf2	
	Mgp	
	Fbln	
	Meg3	
	Gas6	
	Hp	

Table S3. Primer sets for Real-time PCR

	Forward (5'-3')	Reverse (5'-3')
Bcl-xL	TCTGAATGACCACCTAGAGCC	GCTGCATTGTTCCCGTAGAG
Puma	ACCTCAACGCGCAGTACG	GTAGGCACCTAGTTGGGCTC
HO-1	TATGCCCCACTCTACTTCCC	AGTGAGGCCCATACCAGAAG
Collagen I	GCCTCAGAAGAACTGGTACAT	ATCCATCGGTCATGCTCTCT
Collagen III	AGTCAAGGAGAAAGTGGTCG	CCAGGGAAACCCATGACAC
Collagen IV	CGGTACACAGTCAGACCATT	CATCACGAAGGAATAGCCGA
PAI-1	GTCTTTCCGACCAAGAGCAG	GCCGAACCACAAAGAGAAAG
TGF-β	TGATACGCCTGAGTGGCTGTCT	CACAAGAGCAGTGAGCGCTGAA
Gapdh	CAGTATGACTCCACCCACGG	ATGGGCTTCCCGTTGATGAC

Supplementary references

[1] C. Frezza, S. Cipolat, and L. Scorrano, “Organelle isolation: functional mitochondria from mouse liver, muscle and cultured filroblasts,” *Nat. Protoc. 2007 22*, vol. 2, no. 2, pp. 287–295, Feb. 2007, doi: 10.1038/nprot.2006.478.

[2] P. M. Quiros, A. Goyal, P. Jha, and J. Auwerx, “Analysis of mtDNA/nDNA ratio in mice,” *Curr. Protoc. Mouse Biol.*, vol. 7, no. 1, p. 47, Mar. 2017, doi: 10.1002/CPMO.21.

[3] P. Bankhead *et al.*, “QuPath: Open source software for digital pathology image analysis,” *Sci. Reports 2017 71*, vol. 7, no. 1, pp. 1–7, Dec. 2017, doi: 10.1038/s41598-017-17204-5.

[4] J. Schindelin *et al.*, “Fiji: an open-source platform for biological-image analysis,” *Nat. Methods 2012 97*, vol. 9, no. 7, pp. 676–682, Jun. 2012, doi: 10.1038/nmeth.2019.

[5] G. Yu, L. G. Wang, Y. Han, and Q. Y. He, “clusterProfiler: an R package for

comparing biological themes among gene clusters," *OMICS*, vol. 16, no. 5, pp. 284–287, May 2012, doi: 10.1089/OMI.2011.0118.

[6] A. Liberzon, A. Subramanian, R. Pinchback, H. Thorvaldsdóttir, P. Tamayo, and J. P. Mesirov, "Molecular signatures database (MSigDB) 3.0," *Bioinformatics*, vol. 27, no. 12, pp. 1739–1740, Jun. 2011, doi: 10.1093/BIOINFORMATICS/BTR260.

[7] A. Subramanian *et al.*, "Gene set enrichment analysis: A knowledge-based approach for interpreting genome-wide expression profiles," *Proc. Natl. Acad. Sci.*, vol. 102, no. 43, pp. 15545–15550, Oct. 2005, doi: 10.1073/PNAS.0506580102.

[8] A. Liberzon, C. Birger, H. Thorvaldsdóttir, M. Ghandi, J. P. Mesirov, and P. Tamayo, "The Molecular Signatures Database (MSigDB) hallmark gene set collection," *Cell Syst.*, vol. 1, no. 6, pp. 417–425, Dec. 2015, doi: 10.1016/J.CELS.2015.12.004.

[9] L. He, H. Yuan, J. Liang, J. Hong, and C. Qu, "Expression of hepatic stellate cell activation-related genes in HBV-, HCV-, and nonalcoholic fatty liver disease-associated fibrosis," *PLoS One*, vol. 15, no. 5, May 2020, doi: 10.1371/JOURNAL.PONE.0233702.

[10] A. B. Marcher *et al.*, "Transcriptional regulation of Hepatic Stellate Cell activation in NASH," *Sci. Reports 2019 91*, vol. 9, no. 1, pp. 1–13, Feb. 2019, doi: 10.1038/s41598-019-39112-6.

[11] M. K. Terkelsen *et al.*, "Transcriptional Dynamics of Hepatic Sinusoid-Associated Cells After Liver Injury," *Hepatology*, vol. 72, no. 6, pp. 2119–2133, Dec. 2020, doi: 10.1002/HEP.31215.

[12] O. Krenkel, J. Hundertmark, T. P. Ritz, R. Weiskirchen, and F. Tacke, "Single Cell RNA Sequencing Identifies Subsets of Hepatic Stellate Cells and Myofibroblasts in Liver Fibrosis," *Cells 2019, Vol. 8, Page 503*, vol. 8, no. 5, p. 503, May 2019, doi: 10.3390/CELLS8050503.

[13] I. Mederacke, D. H. Dapito, S. Affò, H. Uchinami, and R. F. Schwabe, "High-yield and high-purity isolation of hepatic stellate cells from normal and fibrotic mouse livers," *Nat. Protoc.*, vol. 10, no. 2, p. 305, Jan. 2015, doi: 10.1038/NPROT.2015.017.

[14] X. Han *et al.*, "Mapping the Mouse Cell Atlas by Microwell-Seq," *Cell*, vol. 172, no. 5, pp. 1091-1107.e17, Feb. 2018, doi: 10.1016/J.CELL.2018.02.001.

[15] X. Xiong *et al.*, "Landscape of Intercellular Crosstalk in Healthy and NASH Liver Revealed by Single-Cell Secretome Gene Analysis," *Mol. Cell*, vol. 75, no. 3, pp. 644-660.e5, Aug. 2019, doi: 10.1016/J.MOLCEL.2019.07.028.

[16] N. Schaum *et al.*, "Single-cell transcriptomics of 20 mouse organs creates a Tabula Muris: The Tabula Muris Consortium," *Nature*, vol. 562, no. 7727, p. 367, Oct. 2018, doi: 10.1038/S41586-018-0590-4.

[17] O. Govaere *et al.*, "Transcriptomic profiling across the nonalcoholic fatty liver disease spectrum reveals gene signatures for steatohepatitis and fibrosis," *Sci. Transl. Med.*, vol. 12, no. 572, Dec. 2020, doi: 10.1126/SCITRANSLMED.ABA4448.

[18] L. Pantano *et al.*, "Molecular characterization and cell type composition deconvolution of fibrosis in NAFLD," *Sci. Rep.*, vol. 11, no. 1, Dec. 2021, doi: 10.1038/s41598-021-96966-5.
