# Supplementary material for: Cyclo(His-Pro): A further step in the management of steatohepatitis
Source: JHEP Rep. 2023 Jun 10;5(9):100815. doi: 10.1016/j.jhepr.2023.100815 (PMC10432811; doi:10.1016/j.jhepr.2023.100815)
Supplement: Multimedia component 2 [file mmc2.docx]

**JHEP Reports**

**CTAT methods**

Tables for a “Complete, Transparent, Accurate and Timely account” (CTAT) are now mandatory for all revised submissions. The aim is to enhance the reproducibility of methods.

- Only include the parts relevant to your study
- Refer to the CTAT in the main text as ‘Supplementary CTAT Table’
- Do not add subheadings
- Add as many rows as needed to include all information
- Only include one item per row

**If the CTAT form is not relevant to your study, please outline the reasons why:**

|  |
| --- |

- 1. **Antibodies**

| **Name** | **Citation** | **Supplier** | **Cat no.** | **Clone no.** |
| --- | --- | --- | --- | --- |
| rat α-CD45 |  | Thermo Fisher | 30F-11 |  |
| rabbit α-phospho-p44/42 MAPK |  | Cell signaling | 4376 |  |
| rabbit α-p44/42 MAPK |  | Cell signaling | 4695 |  |
| rabbit recombinant α-vinculin |  | Abcam | ab129002 |  |
| rabbit α-αSMA |  | Cell signaling | 19245S |  |
| rabbit α-fibronectin |  | abcam | ab2413 |  |

- 1. **Cell lines**

| **Name** | **Citation** | **Supplier** | **Cat no.** | **Passage no.** | **Authentication test method** |
| --- | --- | --- | --- | --- | --- |
| AML12 |  | ATCC | CRL-2254 | 10-15 |  |

- 1. **Organisms**

| **Name** | **Citation** | **Supplier** | **Strain** | **Sex** | **Age** | **Overall n number** |
| --- | --- | --- | --- | --- | --- | --- |
| Mus musculus |  | Breeders from Charles River | C57BL/6J | male | 24 weeks | 24 |
| Mus musculus |  | Charles River | C57BL/6J | male | 7-17 weeks | 55 |
| Mus musculus |  | Koatech Co. Ltd | C57BL/6J | male | 11-12 weeks | 53 |

- 1. **Sequence based reagents**

| **Name** | **Sequence** | **Supplier** |
| --- | --- | --- |
| Bcl-xL (forward) | TCTGAATGACCACCTAGAGCC | Bioneer |
| Bcl-xL (reverse) | GCTGCATTGTTCCCGTAGAG | Bioneer |
| Puma (forward) | ACCTCAACGCGCAGTACG | Bioneer |
| Puma (reverse) | GTAGGCACCTAGTTGGGCTC | Bioneer |
| HO-1 (forward) | TATGCCCCACTCTACTTCCC | Bioneer |
| HO-1 (reverse) | AGTGAGGCCCATACCAGAAG | Bioneer |
| Collagen I (forward) | GCCTCAGAAGAACTGGTACAT | Bioneer |
| Collagen I (reverse) | ATCCATCGGTCATGCTCTCT | Bioneer |
| Collagen III (forward) | AGTCAAGGAGAAAGTGGTCG | Bioneer |
| Collagen III (reverse) | CCAGGGAAACCCATGACAC | Bioneer |
| Collagen IV (forward) | CGGTACACAGTCAGACCATT | Bioneer |
| Collagen IV (reverse) | CATCACGAAGGAATAGCCGA | Bioneer |
| PAI-1 (forward) | GTCTTTCCGACCAAGAGCAG | Bioneer |
| PAI-1 (reverse) | GCCGAACCACAAAGAGAAAG | Bioneer |
| TGF-β (forward) | TGATACGCCTGAGTGGCTGTCT | Bioneer |
| TGF-β (reverse) | CACAAGAGCAGTGAGCGCTGAA | Bioneer |
| Gapdh (forward) | CAGTATGACTCCACCCACGG | Bioneer |
| Gapdh (reverse) | ATGGGCTTCCCGTTGATGAC | Bioneer |

- 1. **Biological samples**

| **Description** | **Source** | **Identifier** |
| --- | --- | --- |
|  |  |  |

- 1. **Deposited data**

| **Name of repository** | **Identifier** | **Link** |
| --- | --- | --- |
| Gene Expression Omnibus (GEO) | GSE200750, GSE216366, GSE230745 | https://www.ncbi.nlm.nih.gov/geo/ |

- 1. **Software**

| **Software name** | **Manufacturer** | **Version** |
| --- | --- | --- |
| BioRender | Biorender.com |  |
| Graphpad PRISM | GraphPad Software, LLC | 9.5.1 |
| Adobe Illustrator 2022 | Adobe Inc. | 26.0.1 |
| ImageJ-Fiji | Schindelin, J., Arganda-Carreras, I., Frise, E., Kaynig, V., Longair, M., Pietzsch, T., … Cardona, A. (2012). Fiji: an open-source platform for biological-image analysis. Nature Methods, 9(7), 676–682. doi:10.1038/nmeth.2019 |  |
| QuPath | Bankhead, P. et al. QuPath: Open source software for digital pathology image analysis. Scientific Reports (2017).  https://doi.org/10.1038/s41598-017-17204-5 | 0.3.2 |

- 1. **Other (*e.g*. drugs, proteins, vectors etc.)**

| Cyclo (His-Pro) | NOV MetaPharma |  |
| --- | --- | --- |
| Pyruvate | Sigma-Aldrich |  |
| Malate | Sigma-Aldrich |  |
| Glutamate | Sigma-Aldrich |  |
| ADP+Mg2+ | Sigma-Aldrich |  |
| Succinate | Sigma-Aldrich |  |
| Rotenone | Sigma-Aldrich |  |
| Antimycin A | Sigma-Aldrich |  |
| Oligomycin | Sigma-Aldrich |  |
| carbonyl cyanide-p-trifluoromethoxyphenylhydrazone | Sigma-Aldrich |  |

- 1. **Please provide the details of the corresponding methods author for the manuscript:**

| Johan Auwerx, Laboratory of Integrative Systems Physiology, Institute of Bioengineering, École Polytechnique Fédérale de Lausanne, Lausanne 1015, Switzerland. Tel.: +41 216939522. E-mail address: admin.auwerx@epfl.ch |
| --- |

**2.0 Please confirm for randomised controlled trials all versions of the clinical protocol are included in the submission. These will be published online as supplementary information.**

|  |
| --- |
