# Supplementary material for: Cyclo(His-Pro): A further step in the management of steatohepatitis
Source: JHEP Rep. 2023 Jun 10;5(9):100815. doi: 10.1016/j.jhepr.2023.100815 (PMC10432811; doi:10.1016/j.jhepr.2023.100815)
Supplement: Multimedia component 4 [file mmc4.pdf]

Research article

JHEP|Reports

Cyclo(His-Pro): A further step in the management of steatohepatitis

Alessia De Masi,[1] Xiaoxu Li,[1] Dohyun Lee,[2] Jongsu Jeon,[2] Qi Wang,[1] Seoyeong Baek,[2] Onyu Park,[2] Adrienne Mottis,[1] Keno Strotjohann,[1] Alexis Rapin,[1] Hoe-Yune Jung,[2,3,*] Johan Auwerx[1,*]

[1]*Laboratory of Integrative Systems Physiology, Institute of Bioengineering, École Polytechnique Fédérale de Lausanne, Lausanne, Switzerland;* [2]*R&D Center, NovMetaPharma Co., Ltd., Pohang, South Korea;* [3]*School of Interdisciplinary Bioscience and Bioengineering, Pohang University of Science and Technology (POSTECH), Pohang, South Korea*

JHEP Reports **2023.** https://doi.org/10.1016/j.jhepr.2023.100815

Background & Aims: Non-alcoholic fatty liver disease (NAFLD) and steatohepatitis (NASH) have become the world's most common liver diseases, placing a growing strain on healthcare systems worldwide. Nonetheless, no effective pharmacological treatment has been approved. The naturally occurring compound cyclo histidine-proline (His-Pro) (CHP) is an interesting candidate for NAFLD management, given its safety profile and anti-inflammatory effects.
Methods: Two different mouse models of liver disease were used to evaluate protective effects of CHP on disease progression towards fibrosis: a model of dietary NAFLD/NASH, achieved by thermoneutral housing (TN) in combination with feeding a western diet (WD), and liver fibrosis caused by repeated injections with carbon tetrachloride (CCl_4).
Results: Treatment with CHP limited overall lipid accumulation, lowered systemic inflammation, and prevented hyperglycaemia. Histopathology and liver transcriptomics highlighted reduced steatosis and demonstrated remarkable protection from the development of inflammation and fibrosis, features which herald the progression of NAFLD. We identified the extracellular signal-regulated kinase (ERK) pathway as an early mediator of the cellular response to CHP.
Conclusions: CHP was active in both the preventive and therapeutic setting, reducing liver steatosis, fibrosis, and inflammation and improving several markers of liver disease.
Impact and implications: Considering the incidence and the lack of approved treatments, it is urgent to identify new strategies that prevent and manage NAFLD. CHP was effective in attenuating NAFLD progression in two animal models of the disease. Overall, our work points to CHP as a novel and effective strategy for the management of NAFLD, fuelling optimism for potential clinical studies.
© 2023 The Authors. Published by Elsevier B.V. on behalf of European Association for the Study of the Liver (EASL). This is an open access article under the CC BY license (http://creativecommons.org/licenses/by/4.0/).

Introduction

Non-alcoholic fatty liver disease (NAFLD) is the most common liver disease worldwide, currently affecting 25% of the adult population.[1–3] NAFLD development is closely intertwined with obesity, type 2 diabetes, impaired lipid metabolism, and insulin resistance, conditions whose incidence have also increased over the past decades.[4,5] NAFLD refers to a broad spectrum of liver histological alterations, typified by hepatocellular steatosis, that ranges from isolated steatosis to non-alcoholic steatohepatitis (NASH).[6] Statistically, 30% of patients with NAFLD will develop NASH, characterised by additional hepatocellular ballooning and inflammation.[7,8] If left untreated, NAFLD can eventually progress to liver fibrosis, cirrhosis, and even hepatocellular carcinoma.[9,10] Currently, there are no FDA-approved treatments, and the best therapeutic option available is based on lifestyle changes, which are often hindered by lack of compliance.[11] It is therefore urgent to find safe and effective strategies to manage hepatic lipid accumulation, chronic inflammation, and fibrosis in patients with NAFLD.

Cyclo histidine-proline (His-Pro) (CHP) is an endogenous cyclic dipeptide derived from the thyrotropin-releasing hormone (TRH).[12] Pyroglutamyl-peptidase degrades TRH and forms His-Pro dipeptides, which undergo spontaneous cyclisation at 37 °C.[13,14] This cyclisation confers higher stability and protection against peptidases. This is required for its active transport in the intestine[15] and through the blood–brain barrier,[16,17] allowing CHP to be distributed systemically.[18] CHP is present in several food sources[19] and can be safely administered *per os.*[20,21] CHP started to gain scientific interest as several studies outlined its cytoprotective role in neuronal models.[22–25] CHP has also been evaluated as a drug candidate in the context of diabetes, as it helps to control blood glucose levels and insulin response.[26–29] At the molecular level, CHP is known to trigger anti-oxidant[30] and anti-inflammatory responses;[31] however, the biological

Keywords: Cyclo histidine-proline (Cyclo(His-Pro)); Non-alcoholic fatty liver disease; Non-alcoholic steatohepatitis; Liver; Transcriptomics; Prevention; Inflammation; Liver fibrosis; Therapeutics; Drug development.
Received 28 October 2022; received in revised form 8 May 2023; accepted 25 May 2023; available online 10 June 2023
* Corresponding authors. Addresses: Laboratory of Integrative and Systems Physiology, École Polytechnique Fédérale de Lausanne, CH-1015 Lausanne, Switzerland. Tel.: +41-216-939-522 (J. Auwerx); and R&D Center, NovMetaPharma Co., Ltd., Pohang, 37668, South Korea. Tel.: +82 (0)54-223-2893 (H.-Y. Jung).
E-mail addresses: admin.auwerx@epfl.ch (J. Auwerx), elijah98@novmeta.com (H.-Y. Jung).

ELSEVIER

EASL The Home of Hepatology

role of CHP, as well as its mechanism of action, have remained elusive.

In this study, we evaluated whether CHP administration could prevent inflammation and fibrosis in liver disease. Using a murine model of NAFLD, we demonstrated that CHP counteracts weight gain and body fat accumulation. Administration of CHP improved the overall liver phenotype, including reduced liver steatosis, fibrosis, and inflammation. The efficacy of the treatment was confirmed in a second liver injury model, which was induced by repeated low-dose carbon tetrachloride (CCl_4) injections. In this model, widely used to study liver fibrosis, we provide a first indication that CHP not only has the potential to prevent liver disease, but it can also be used as a therapeutic treatment. Thus, our *in vivo* studies provide a rationale for both the preventive and the therapeutic effects of CHP in the context of NAFLD/NASH. Transcriptomic and molecular studies confirmed the robust anti-inflammatory effects of CHP, leading us to identify the extracellular signal-regulated kinase (ERK) cascade as a relevant signalling pathway involved in the response to CHP treatment and its impact on mitochondrial function.

Materials and methods

Animal studies

Diet-induced NAFLD

C57BL/6J male mice (Charles River) were transferred to thermoneutral housing (TN) cabinets (30–32 °C) at 6 weeks of age. At Week 7, the animals received the chow diet (CD) (Research Diet D16042904B) or the western diet (WD) (Research Diet D12079B). Treatment with CHP was started at Week 7, 20 mg/kg doses were administered 3 times a week *per os* (gavage) throughout the study. Water was used as a control treatment. Body weight was monitored weekly. Phenotyping started at Week 18 with a non-invasive monitoring of fat and lean mass using the Echo Medical Systems magnetic resonance imaging (MRI) device as previously described.[32] Food intake, caloric consumption, and respiratory quotient were measured using Promethion cages (Sable Systems). At Week 20, mice were subjected to an overnight fast. Tail vein glucose levels were measured using the CONTOUR®NEXT glucometer (Ascensia Diabetes Care). Insulin levels were measured with the Ultra-Sensitive Mouse Insulin ELISA Kit (Crystal Chem). Homeostatic model assessment (HOMA)[33] was used to calculate the insulin-resistance (IR) index as follows: HOMA-IR = [fasting blood glucose (mmol/L) × serum insulin (mIU/L)]/22.5. At Week 24, after a 3-h fast, mice were euthanised to collect tissues, of which the weight was measured. For biochemical analysis, tissues were collected and flash-frozen, and stored at −80 °C. In a follow-up study, mice were housed at TN, fed with either CD or WD and treated with CHP for 8 weeks. Faeces were collected at Week 7 of the study for a 24-h period and caloric content was quantified with a calorimeter bomb (IKA C5003, IKA-Werke GmbH & Co. KG, Staufen, Germany). To study the ERK response, 6-week-old C57BL/6J male mice were treated with 20 mg/kg of CHP *per os*. Water was used as a control treatment. Mice were euthanised to collect the liver at 4 or 24 h after treatment.

CCl_4-induced liver fibrosis

Eight-week-old male C57BL/6 mice (Koatech Co. Ltd.) were housed at 23 °C with free to access to water and chow diet *ad libitum*. After a week of adaptation, the mice were divided into 3 groups: 0.2 ml/kg of CCl_4 in olive oil (CCl_4:olive oil = 1:19 vol:vol) was injected intraperitoneally (*i.p.*) on Days 1, 3, 6, 8, 10, and 13 for the 2-week study and on Days 1, 3, 6, 8, 10, 13, 15, 17, and 20 for the 3-week study; the control group received *i.p.* injection of an equal volume of olive oil. Distilled water (vehicle) or 35 mg/kg of CHP were administered daily by oral gavage, after the first (preventive study) or third (therapeutic study) CCl_4 injection. Mice were euthanised 12 h after the last CCl_4 injection, to collect plasma and tissues.

RNA-seq

In the NAFLD study, RNA was extracted in Trizol (TriPure, Roche) from frozen liver, kidney, or gastrocnemius tissue, using the Direct-zol-96 RNA Kit (Zymo Research). Bulk RNA-seq of extracted RNA from each mouse was performed by the Beijing Genomics Institute with the BGISEQ-500 platform. In the CCl_4 study, RNA was extracted with NucleoZOL reagent (Macherey-Nagel) and paired-end sequencing was performed by Macrogen Incorporated with Illumina NovaSeq. Clean reads were obtained by removing adapter sequences or low-quality sequences (RIN <8) based on SOAPnuke software.[34] RNA-seq data and related analyses were performed using the R version 3.5.2.[35] All samples passed the quality check by FastQC.[36,37] Sequences were aligned against the mouse genome, using STAR (version 2.7.3a).[38] Two comparisons (WD *vs.* CD, WD + CHP *vs.* WD for the NAFLD study; CCl_4 *vs.* control [CTRL], CCl_4 + CHP *vs.* CCl_4 for the CCl_4-induced model) were analysed by differential expression analysis (DEA) using the limma R package (version 3.38.3).[39,40] Further detailed information for the RNA-seq analysis can be found in the Supplementary methods.

Cell experiments

AML-12 cell line was obtained from ATCC and grown at 37 °C in a humidified atmosphere of 5% CO_2 and 95% air in DMEM/F12 supplemented with FBS 10% (Gibco), 0.005 mg/ml insulin, 0.005 mg/ml transferrin, 5 ng/ml selenium, and 40 ng/ml dexamethasone. Cells were treated with CHP 100 nM for 4 or 24 h, then samples were recovered for protein extraction.

Statistics

GraphPad Prism v9.5.1 was used for all statistical analyses. Differences between groups were assessed using the unpaired *t*-test, one-way or two-way analysis of variance (ANOVA), followed by the Dunnett multiple comparison test, as specified in the figure legends. All p values < 0.05 were considered significant.

Study approval

The WD/TN study and the follow-up experiments were conducted following Swiss ethical guidelines and were authorised by the animal experimentation committee of the Canton de Vaud (VD3313). The CCl_4-induced liver disease study was approved by the Ethics Review Committee of the Pohang Advanced Bio Convergence Center, Pohang, Republic of Korea (ABCC2022102).

Results

CHP counteracted diet-induced metabolic syndrome

NAFLD was induced in 24-week-old male C57BL/6J mice by housing animals at TN (30°C–32 °C) and feeding with a WD, enriched in fat (21%) and sucrose (35%) (Fig. 1A). This combination of challenges enhances liver fat accumulation and reproduces most of the key pathological features of NAFLD.[41,42]

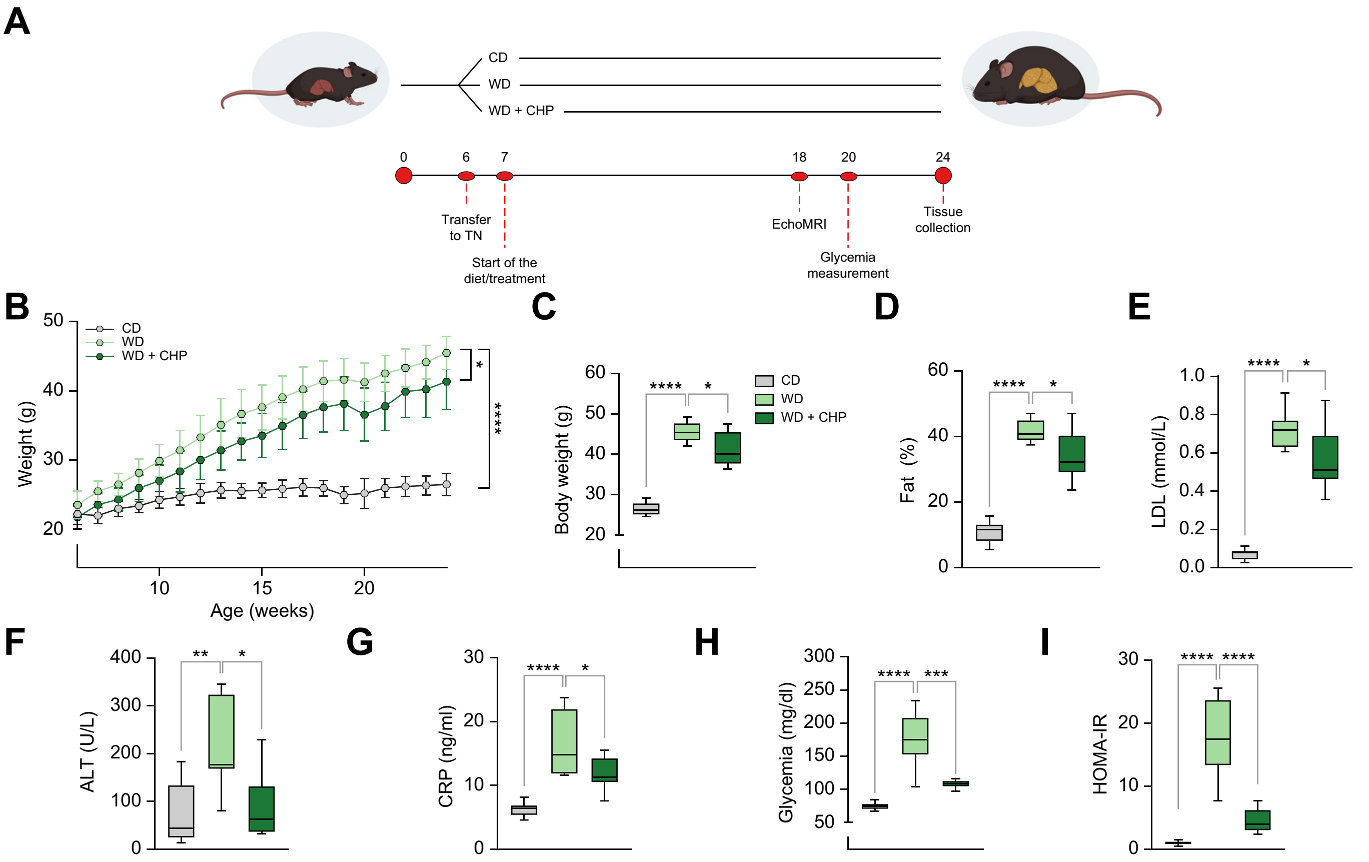

Fig. 1. CHP prevents weight gain and fat accumulation and improves plasma biomarkers in the NAFLD mouse model. (A) Our NAFLD model was established through a combination of WD and thermoneutrality housing. Mice were housed in a thermoneutrality cabinet from 6 weeks of age. A specific diet (CD/WD) was introduced at Week 7. Treatment with CHP (20 mg/kg) was started at Week 7 and was given by gavage 3 times per week, for the whole duration of the study. Evaluation of fat mass by EchoMRI™ was performed at Week 18. Fasting glycaemia was measured at Week 20. Mice were euthanised at Week 24, after a 3-h fast. (B) Body weight increase over time. Weight was recorded once a week. (C) Body weight at Week 24. (D) Body fat percentage measured at Week 18 by MRI. (E–G) LDL-cholesterol (E), ALT (F), and CRP (G) levels in plasma. Blood was collected at the end of the experiment. (H) Glycaemia as measured after an overnight (14 h) fast. (I) HOMA-IR index for each group, calculated following the homeostatic model assessment (n = 6–8). Results represent the mean ± standard deviation (B) and the whiskers in boxplots represent the minimum to maximum range (C–I). Two-way ANOVA (B) and one-way ANOVA (C–I), followed by Dunnett's multiple comparison test *vs.* WD group, were used for statistical analysis. $^{*}p$ <0.05; $^{**}p$ <0.01; $^{***}p$ <0.001; $^{****}p$ <0.0001. ALT, alanine transaminase; ANOVA, analysis of variance; CD, chow diet; CHP, cyclo(His-Pro); CRP, C-reactive peptide; HOMA-IR, homeostatic model assessment-insulin resistance; LDL, low-density lipoprotein; MRI, magnetic resonance imaging; NAFLD, non-alcoholic fatty liver disease; TN, thermoneutral housing; WD, western diet.

Mice fed with a matched control CD were used as an additional control group. CHP administration (20 mg/kg) slightly limited the weight gain caused by WD feeding (Fig. 1B and C) despite equal food intake and caloric consumption in the 2 groups (Fig. S1A, B). Notably, this was attributed to CHP effects on nutrient absorption (Fig. S1C). The reduced weight gain was reflected in a lower body fat mass, with an average reduction of 8% in fat mass after CHP administration, as quantified by MRI using the EchoMRI™ device (Fig. 1D). CHP attenuated the increase in plasma low-density lipoprotein (LDL)-cholesterol levels induced by the WD (Fig. 1E). Plasma alanine transaminase (ALT) was evaluated as a marker of liver dysfunction. Mice receiving WD had higher ALT levels, a phenomenon that was not observed in CHP-treated animals (Fig. 1F). WD increased C-reactive peptide (CRP) levels, suggesting a higher general inflammatory state elicited by the diet, inflammation that was attenuated by exposure to CHP (Fig. 1G). Insulin resistance plays a major role in NAFLD progression.[43,44] Compared with CD feeding, WD-fed mice displayed hyperglycaemia, an effect that was remarkably blunted in CHP-treated animals that maintained glycaemia levels closer to physiological ranges (Fig. 1H). The homeostatic model assessment (HOMA)-insulin resistance (IR) index was calculated from fasting glucose and insulin levels (Fig. 1I), supporting the remarkable prevention of insulin resistance by CHP administration. In this diet-induced NAFLD model, CHP was able to counteract the harmful effects of WD, preventing the onset of metabolic syndrome.

CHP prevented NAFLD progression, reducing hepatic steatosis, fibrosis, and inflammation

The size and colour of the livers in the WD group indicated extensive fat accumulation (Fig. 2A). In contrast, mice treated with CHP had smaller and darker livers, reflecting reduced steatosis (Fig. 2A, B). In blinded histopathological evaluation of tissue sections, CHP reduced the NAFLD activity score (NAS) (Fig. 2C, D). Haematoxylin and eosin (H&E), and Oil Red O (ORO) staining revealed the extensive macro- and micro-vesicular steatosis caused by WD feeding (Fig. 2E). Inflammation and fibrosis, important factors in the progression of NAFLD-NASH, were induced by long-term challenge with WD and TN, as

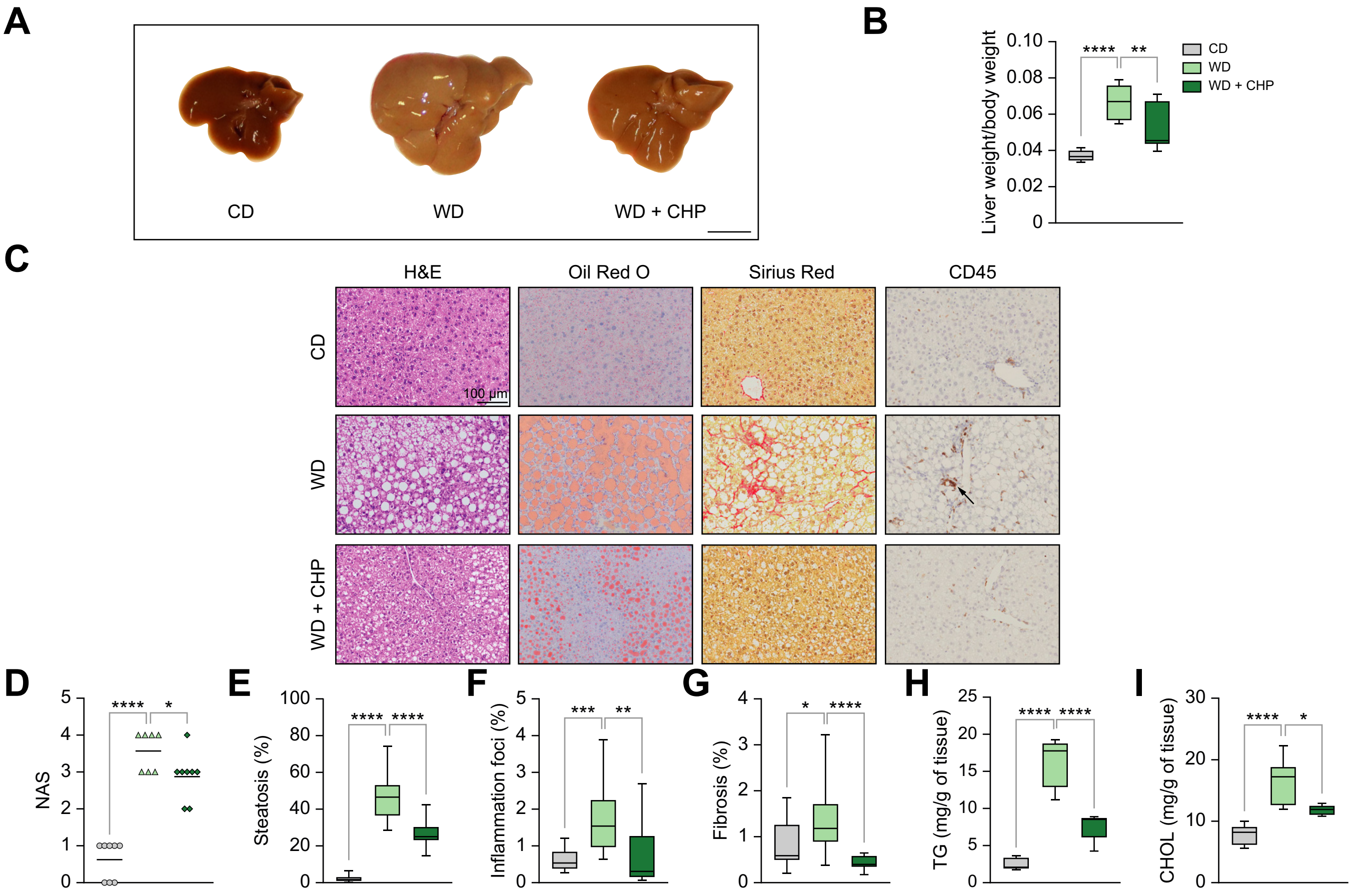

Fig. 2. Liver steatosis, fibrosis and inflammation are prevented by CHP treatment. (A) Comparison of gross liver morphology in representative mice fed with CD/WD or WD supplemented with CHP. Scale bar = 1 cm. (B) Liver weight normalised on body weight, recorded at the end of the experiment (n = 7–8). (C) Representative images of liver sections stained with H&E, ORO, SR, and immunostained with CD45. (D) NAS scoring system, evaluating steatosis, lobular inflammation, and ballooning (n = 7). (E–G) Quantification of ORO (E), CD45$^+$ (F), and SR (G) staining in histological images (n = 16). (H, I) Liver TG (H) and CHOL (I) content normalised by tissue weight (n = 5–6). Individual points are plotted and bar represents mean (D); whiskers in boxplots represent the minimum to maximum range (B, E–I). One-way ANOVA, followed by Dunnett's multiple comparison test *vs.* WD group was used for statistical analysis (B, D–I). *p <0.05; **p <0.01; ***p <0.001; ****p <0.0001. ANOVA, analysis of variance; CD/WD, chow diet/western diet; CHOL, cholesterol; CHP, Cyclo(His-Pro); H&E, haematoxylin and eosin; NAFLD, non-alcoholic fatty liver disease; NAS, NAFLD activity score; ORO, Oil Red O; SR, Sirius Red; TG, triglycerides.

indicated respectively, by increased CD45$^+$ immune cell infiltration (Fig. 2F), and spread of fibrotic strands (Fig. 2G, Fig. S2). CHP administration attenuated hepatic steatosis while reducing fibrosis and inflammation (Fig. 2E–G). Protection from steatosis by CHP administration was further confirmed by assessing liver triglycerides (TG) and cholesterol (CHOL) levels (Fig. 2H, I). Taken together, our findings show that CHP hampered NAFLD progression in our model of WD/TN-challenged mice.

Liver transcriptome analysis revealed the deleterious effects of WD on gene expression and their reversal by CHP

Five livers from each treatment condition were randomly selected for RNA sequencing. In the principal component analysis (PCA), these samples were clustered according to diet and treatment (Fig. 3A). Gene expression was analysed to delineate the molecular signature of WD and CHP treatment, highlighting significantly up- and downregulated genes, particularly genes showing an opposite response upon exposure to WD and CHP treatment (Fig. S3A, B). The increased expression of transcripts involved in collagen synthesis (*e.g. Col1a1*, *Col3a1*, *Tipm1*) and lipid metabolism (*e.g. Fabp4*, *Cidea*) was reversed upon CHP treatment (Fig. 3B). CHP had a major influence on the transcriptome with 338 and 699 transcripts that were respectively significantly up- and downregulated compared to the WD condition (Fig. S3C). Gene set-enrichment analysis (GSEA) confirmed that lipid metabolism-related gene sets were upregulated upon the WD/TN challenge, as well as gene sets involved in inflammation (*e.g.* cytokine production and the nuclear factor kappa B [NF-κB] axis) and fibrosis (*e.g.* the extracellular matrix, hepatic stellate cells [HSC] activation, and collagen production) (Fig. 3C, Fig. S3D). According to histological observations, CHP downregulated the expression of many key genes involved in inflammation and fibrosis (Fig. S3D, E). Oxidative stress, apoptosis, and stress responses followed the same trend (Fig. 3C, Fig. S3D). A cell type enrichment analysis for 29 cell types showed that pro-fibrotic cells (including HSCs, stromal cells, and cholangiocytes) and immune cells (macrophages, B cells, and T cells) were enriched by WD and reduced by treatment with CHP (Fig. 3D). A single-cell deconvolution analysis, which quantified the proportion of different cell populations in the liver bulk RNA-seq data, highlighted the reduced infiltration of immune cells in liver tissue after CHP treatment (Fig. 3E, F). CHP did not appear to

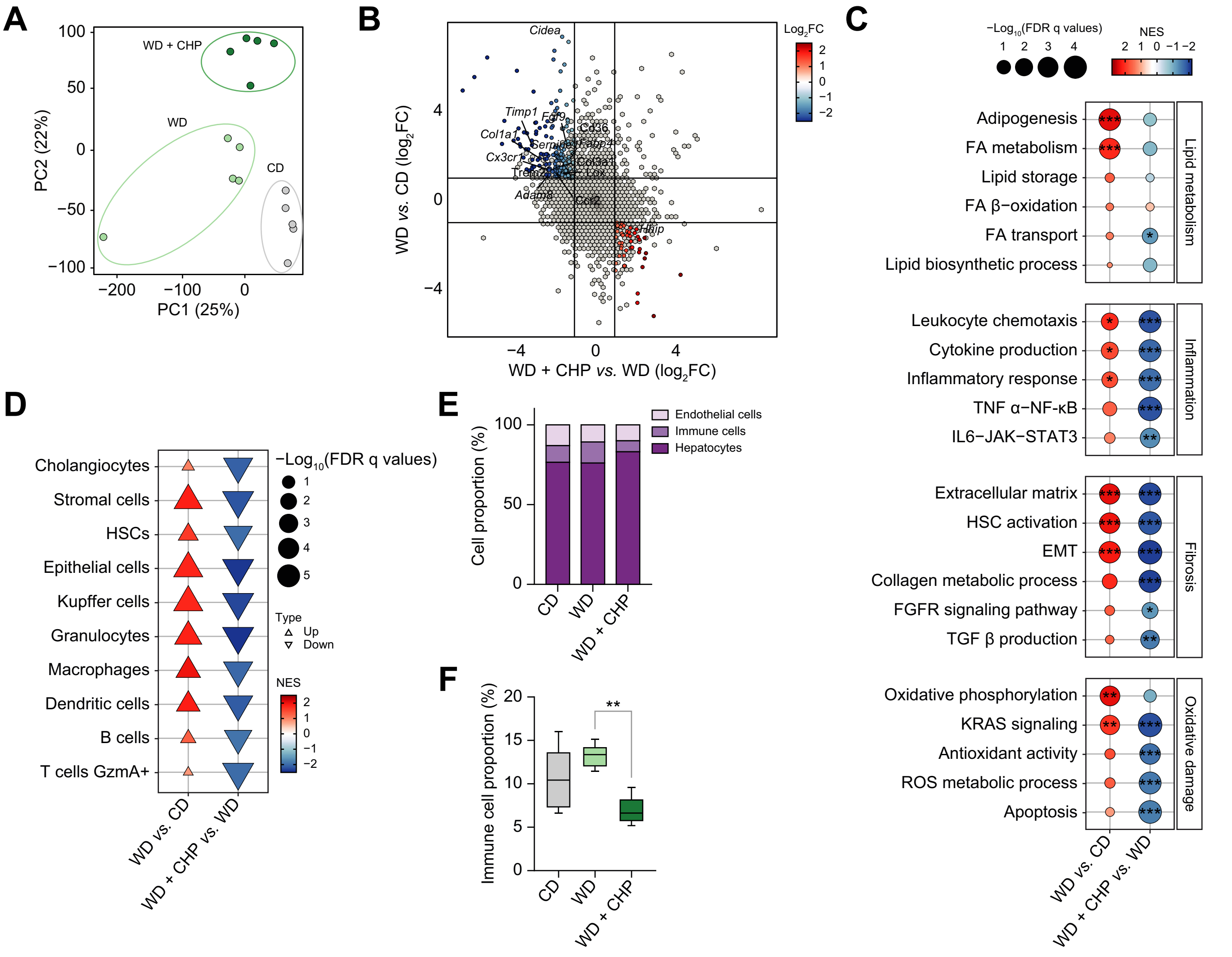

Fig. 3. Transcriptomic signature of CHP in liver. (A) PCA plot of the RNA-seq profiles of the 3 groups. (B) Scatter plot shows the effects of WD compared to CD and the therapeutic effects of CHP on gene expression. The significant genes that show a reversal of the effect of WD compared with CHP treatment are highlighted in blue (upregulated upon WD) and red (upregulated upon treatment). Representative genes are annotated. (C) GSEA of disease (WD) and treatment (CHP) effects on gene expression. Gene sets are grouped into 4 categories: lipid metabolism, inflammation, fibrosis, and oxidative damage. *Q <0.05; **Q <0.01; ***Q <0.001. (D) Cell type enrichment analysis of disease (WD) and treatment (CHP) effects on gene expression. (E) Single cell deconvolution results obtained from integrating the FACS scRNA data available under the GSE109774 accession and our bulk RNA-seq dataset, showing the relative depletion of immune cells upon CHP treatment. (F) Single-cell deconvolution estimated grouped cell proportions of immune cells in different conditions. Whiskers in boxplots represent the minimum to maximum range. One-way ANOVA, followed by Dunnett's multiple comparison test *vs.* WD group was used for statistical analysis. **p <0.01. ANOVA, analysis of variance; CD, chow diet; CHP; cyclo(His-Pro); EMT, epithelial–mesenchymal transition; FA, fatty acids; FC, fold change; FDR, false discovery rate; FGFR, fibroblast growth factor receptor; GSEA, gene set enrichment analysis; HSC, hepatic stellate cells; NES, normalized enrichment score; NF-κB, nuclear factor kappa B; PCA, principal component analysis; ROS, reactive oxygen species; TGF-β, transforming growth factor beta; TNF-α, tumour necrosis factor alpha; WD, western diet.

attenuate the macroscopic effect induced by the WD on the weight of extrahepatic organs (Fig. S4A). To verify the effects of CHP on extrahepatic organs, the kidneys and gastrocnemius muscles were randomly selected for RNA sequencing, as previously described for liver tissue samples. Approximately 20–55% of patients with NAFLD develop chronic kidney disease.[45] An indication for kidney injury—indicated here by the urine albumin-to-creatinine ratio (ACR)—was observed in mice receiving WD and improved following CHP treatment (Fig. S4B). Furthermore, studies have also shown that NAFLD/NASH can also impact on muscle tissue.[46,47] The effect of CHP in the transcriptome of kidney and muscle aligned with its gene profile in the liver, with both inflammation and fibrosis decreasing upon treatment (Fig. S4C), pointing in the direction of a conserved mechanism of action across tissues.

CHP alleviates CCl_4-induced liver damage in mice

As a second model of early liver fibrosis, C57BL/6 mice received repeated low-dose (0.2 ml/kg) CCl_4 injections over 2 weeks, together with daily CHP treatment (35 mg/kg by oral gavage) (Fig. 4A). CCl_4 injection led to a significant increase in plasma ALT levels that was decreased by CHP treatment, suggesting prevention of liver damage (Fig. 4B). The observed reduction in plasma interleukin 6 (IL-6) and tumour necrosis factor alpha (TNF-α) levels confirmed the anti-inflammatory activity of CHP treatment (Fig. 4C, D). Analysis of liver gene expression showed

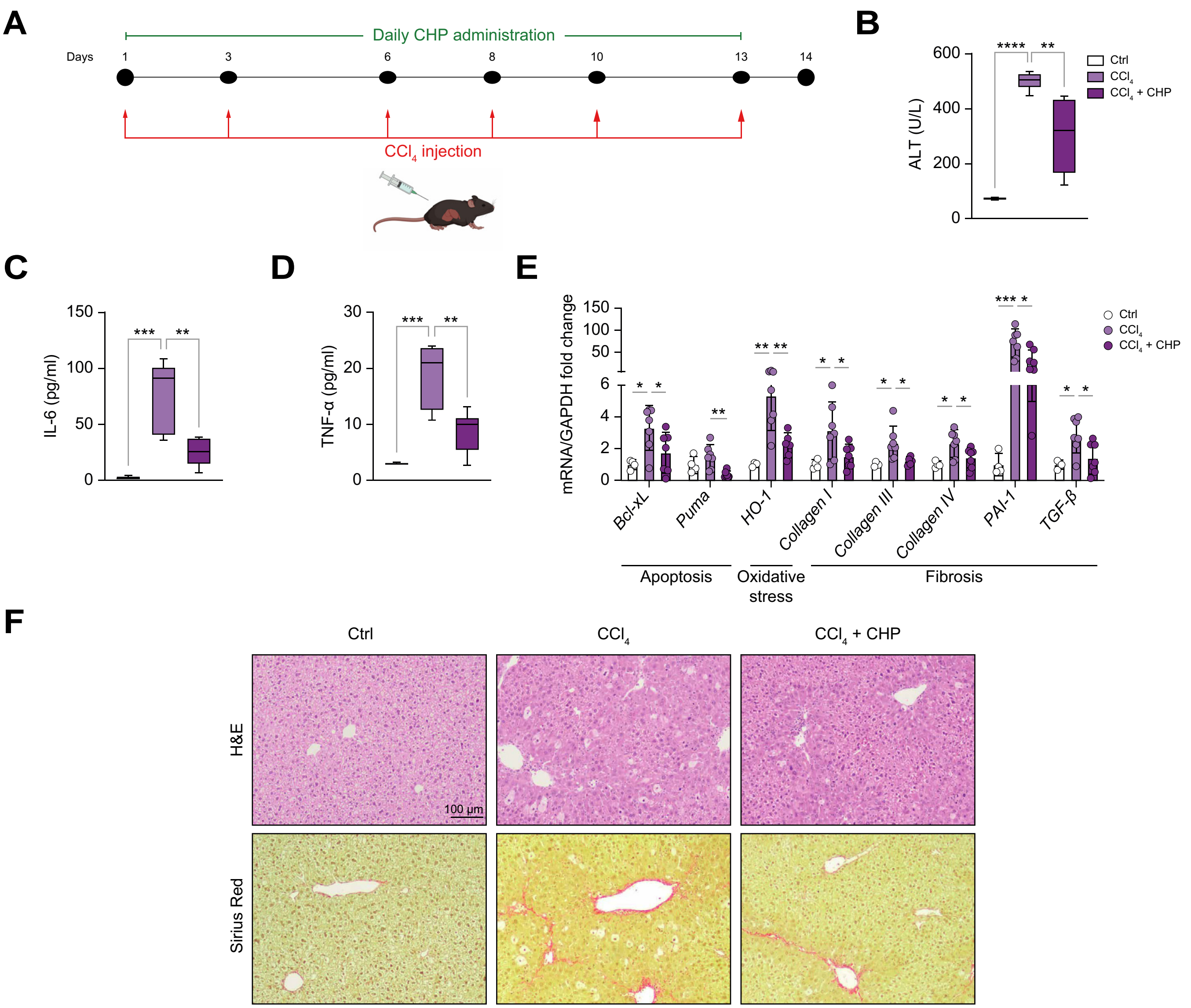

Fig. 4. CHP prevents liver injury caused by 2 weeks of CCl_4 injections. (A) Animal study flow. Mice received 6 injections of CCl_4 over 13 days and were treated with CHP daily. Liver and plasma samples were collected at Day 14. (B–D) ALT (B), IL-6 (C), and TNF-α (D) plasma levels. Whiskers in boxplots represent the minimum to maximum range. (E) Liver expression of genes involved in apoptosis, oxidative stress, and inflammation. Error bars in bar plots represent the standard deviations. (F) Representative images of liver sections stained with H&E or SR. (n = 4 for CTRL, n = 6–7 for treatments). One-way ANOVA, followed by Dunnett's multiple comparison test *vs.* CCl_4 group was used for statistical analysis (B–E). **p* <0.05; ***p* <0.01; ****p* <0.001; *****p* <0.0001. ALT, alanine transaminase; ANOVA, analysis of variance; Bcl-xL, B-cell lymphoma-extra large; CCl_4, carbon tetrachloride; CHP, cyclo(His-Pro); CTRL, control; GAPDH, glyceraldehyde 3-phosphate dehydrogenase; H&E, haematoxylin and eosin; HO-1, heme oxygenase-1; IL-6, interleukin 6; PAI-1, plasminogen activator inhibitor 1; SR, Sirius Red; TGF-β, transforming growth factor beta; TNF-α, tumour necrosis factor alpha.

how CHP downregulated apoptosis, oxidative stress, and fibrosis pathways (Fig. 4E). Hepatic H&E histological sections revealed the extensive cellular hypertrophy caused by CCl_4 (Fig. 4F). Sirius Red (SR) staining uncovered early signs of fibrosis induced by chronic CCl_4 injections (Fig. 4F). Treatment with CHP ameliorated the phenotype by reducing the presence of hypertrophic cells.

The protective effect of CHP against advanced CCl_4-induced liver fibrosis was also evaluated in mice that underwent a longer protocol of CCl_4 injections (Fig. S5A). Liver enzymes (ALT and aspartate transaminase [AST] levels) suggested reduced liver damage after CHP administration (Fig. S5B, C). This was further confirmed by histological analysis, which indicated that cellular hypertrophy and fibrosis, induced by prolonged CCl_4 administration, were dampened by CHP (Fig. S5D).

Liver samples from the 2-week protocol (Fig. 4A) were further analysed by RNA sequencing to characterise the transcriptional effect of CHP. In the PCA, the samples were separated according to the experimental group (Fig. 5A). GSEA revealed that CCl_4 strongly upregulated pathways involved in lipid metabolism and inflammation (Fig. 5B). According to the histological findings, profibrotic processes (*e.g.* HSC activation, epithelial–mesenchymal transition (EMT), extracellular matrix deposition) were upregulated by CCl_4. Transcriptional evidence of CCl_4 toxicity included the upregulation of apoptosis and reactive oxygen species (ROS)-mediated stress response genes (Fig. 5B). CHP potently attenuated pathways associated with inflammation and fibrosis, as well as apoptosis and oxidative damage, in this model of liver injury. Cell-type enrichment analysis confirmed a

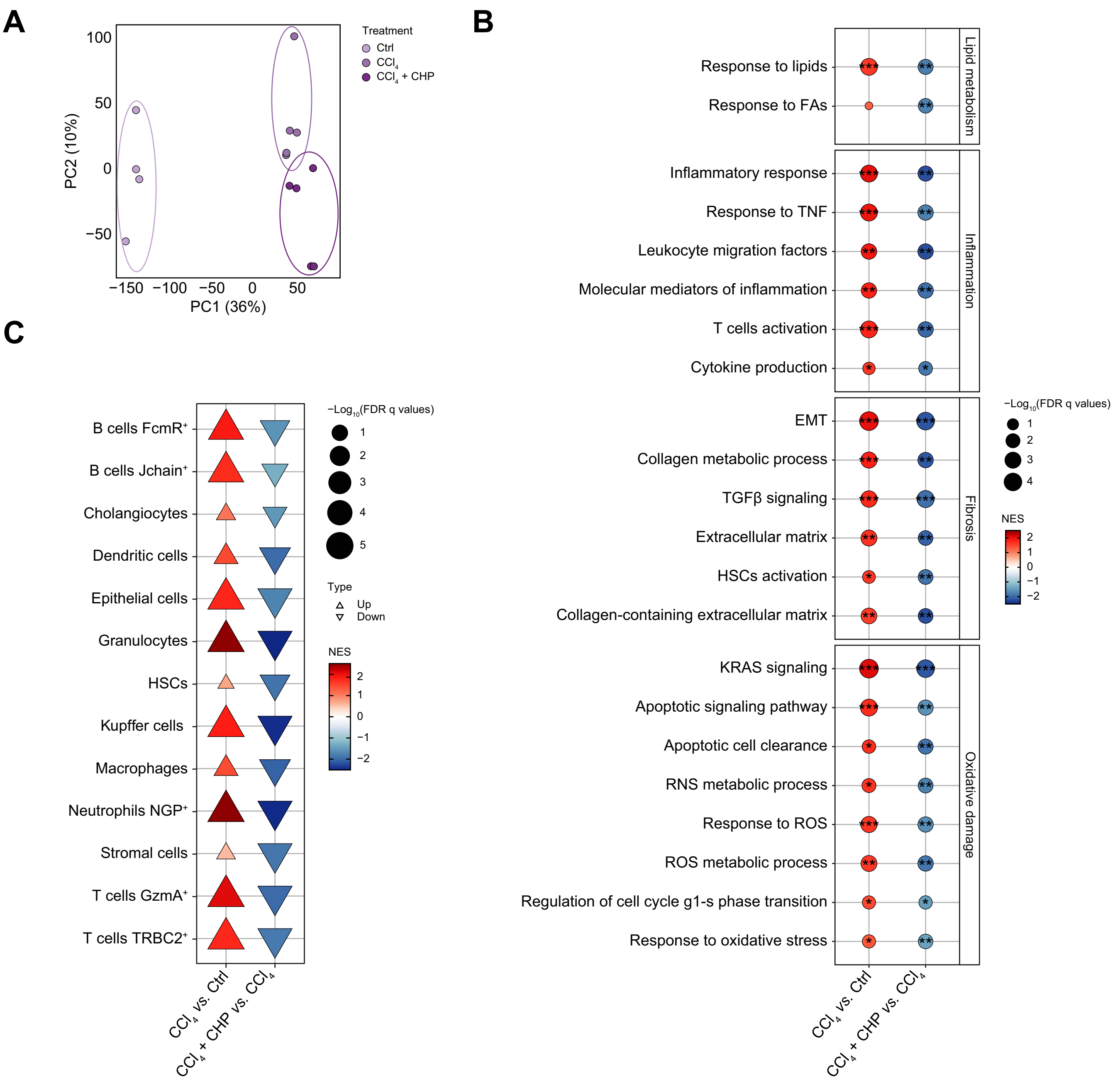

Fig. 5. CHP prevents inflammation and fibrosis caused by CCl_4 in liver. (A) PCA plot of the RNA-seq profiles. (B) GSEA of disease model (CCl_4) and treatment (CHP) effects on gene expression. Gene sets are grouped in 4 categories: lipid metabolism, inflammation, fibrosis, and oxidative damage. *Q <0.05; **Q <0.01; ***Q <0.001. (C) Cell type enrichment analysis of disease model (CCl_4) and treatment (CHP) effects on gene expression. CCl_4, carbon tetrachloride; CHP, cyclo(His-Pro); CTRL, control; FDR, false discovery rate; EMT, epithelial–mesenchymal transition; FA, fatty acids; GSEA, gene set enrichment analysis; HSC, hepatic stellate cells; NES, normalized enrichment score; NGP, neutrophilic granule protein; PCA, principal component analysis; RNS, reactive nitrogen species; ROS, reactive oxygen species; TGF-β, transforming growth factor beta; TNF, tumour necrosis factor.

decrease in immune cells (*e.g.* B and T cells, macrophages, granulocytes, Kuppfer cells) and pro-fibrotic cells (*e.g.* HSCs, cholangiocytes) upon CHP treatment (Fig. 5C).

A therapeutic intervention attenuated liver fibrosis and inflammation

Our study demonstrated that preventive treatment with CHP can effectively reduce liver fibrosis and inflammation. To investigate whether CHP not only exerted a preventive effect, but could also have an impact upon therapeutic administration, a follow-up experiment was performed using a different model of CCl_4-induced liver disease. C57BL/6 mice received a total of 6 CCl_4 injections over a 2-week period, and daily treatment with CHP was started after the third injection (Fig. 6A). Plasma levels of ALT and AST were reduced by CHP treatment (Fig. 6B, C) and liver transcript levels showed a general reduction in fibrosis and inflammation (Fig. 6D). Alpha smooth muscle actin (αSMA) and fibronectin protein levels also confirmed the reduced deposition

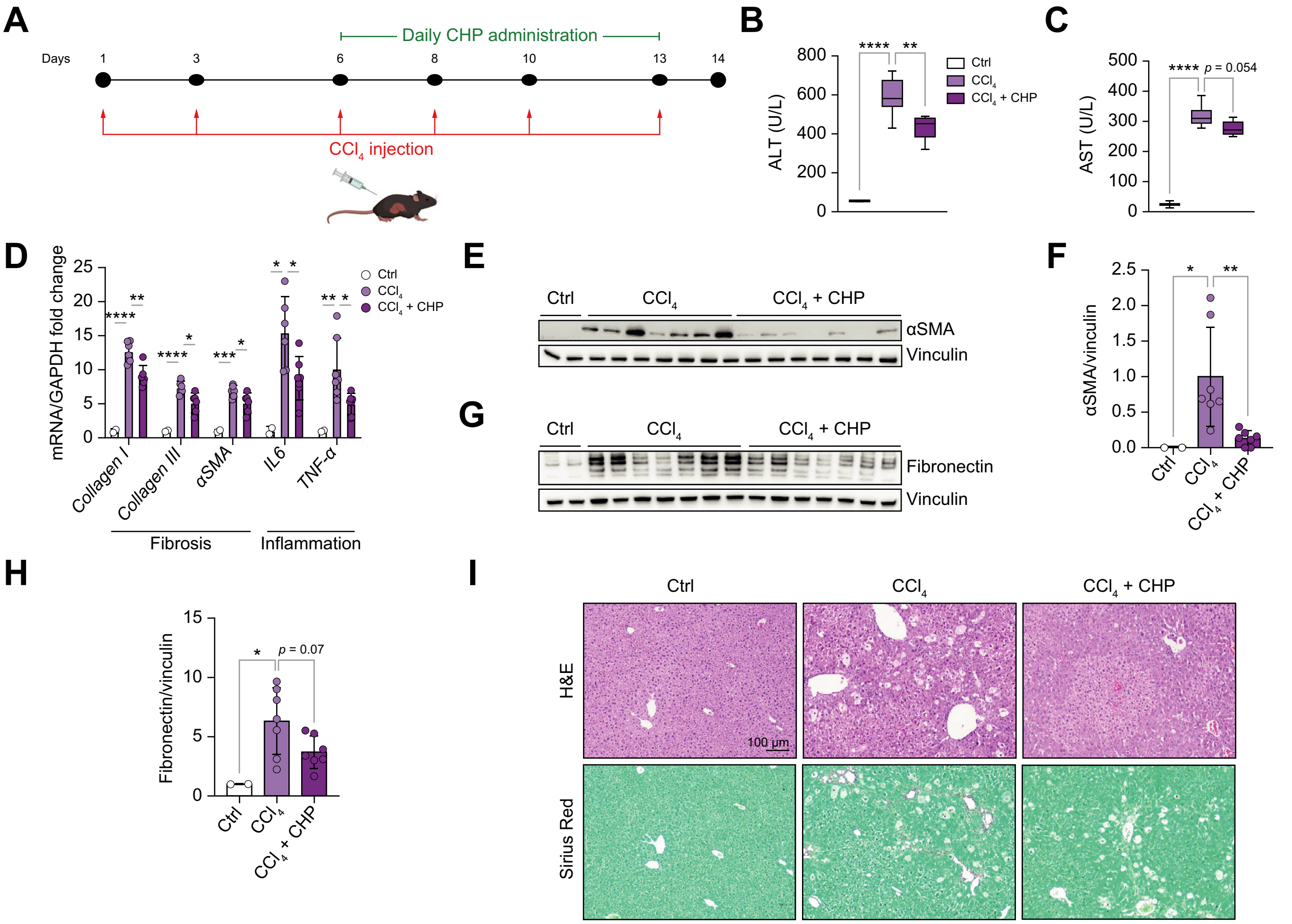

Fig. 6. CHP recovers liver fibrosis and inflammation in a therapeutic protocol. (A) Animal study flow. Mice received 6 injections of CCl_4 over 13 days and were treated with CHP daily starting after the third CCl_4 injection. Liver and plasma were collected at Day 14. (B, C) ALT (B), and AST (C) plasma levels. Whiskers in boxplots represent the minimum to maximum range. (D) Liver expression of genes involved in fibrosis and inflammation. Error bars in bar plots represent the standard deviations. (E) Western blotting of αSMA expression; vinculin was used as loading control. (F) Quantification of αSMA signals from the western blotting in (E), normalised on vinculin. Error bars in bar plots represent the standard deviations. (G) Western blotting of fibronectin expression; vinculin was used as the loading control. (H) Quantification of fibronectin signal from the western blotting in (G), normalised on vinculin. Error bars in bar plots represent the standard deviations. (I) Representative images of liver sections stained with H&E or Sirius Red (0.1% Direct Red and 0.1% Fast Green FCF) (n = 2 for CTRL, n = 6–7 for other treatments). One-way ANOVA, followed by Dunnett's multiple comparison test *vs.* CCl_4 group was used for statistical analysis (B-D, F, H). **p* <0.05; ***p* <0.01; ****p*, <0.001; *****p* <0.0001. ALT, alanine transaminase; AST, aspartate transaminase; CCl_4, carbon tetrachloride; CHP, cyclo(His-Pro); CTRL, control; GAPDH, glyceraldehyde 3-phosphate dehydrogenase; IL-6, interleukin 6; αSMA, alpha smooth muscle actin; TNF-α, tumour necrosis factor alpha.

of extracellular matrix components (Fig. 6E-H). Liver histology supported the ability of CHP to reduce cellular hypertrophy and fibrosis (Fig. 6I, Table S1). Taken together, these results provide strong evidence that CHP can effectively counteract liver inflammation and fibrosis even in a therapeutic setting.

CHP improved mitochondrial function in liver cells

Correct mitochondrial function in hepatocytes is fundamental to maintain energy homeostasis and hepatic metabolic functions. The respiratory quotient of mice, as measured in metabolic cages, gives an indication of the ratio of carbohydrate to fat oxidation and has been previously correlated with cirrhosis and NAFLD progression.[48,49] The respiratory quotient showed a marked decrease upon WD feeding, which trended towards recovery following exposure to CHP (Fig. 7A). CHP positively acted on mitochondria, by increasing complex I activity in isolated mitochondria from liver tissue (Fig. 7B) and by increasing mitochondrial content in the livers of mice challenged with TN/WD (Fig. 7C). To study the effects of CHP on mitochondrial function, alpha mouse liver 12 cells (AML-12) were treated with CHP for 4 h and then cellular respiration was analysed (Fig. 7D). Both basal and maximal respiration were increased by CHP (Fig. 7E), and the increase in basal respiration was specifically due to an increase in complex I activity (Fig. 7F), as observed in liver mitochondria (Fig. 7B). Overall, these data suggest that CHP enhanced mitochondrial number and activity in the liver of our NAFLD model, a finding supported by *in vitro* data on mouse hepatocytes.

Inhibition of ERK signalling was an early effect of CHP treatment

Among the enriched pathways that emerged from the GSEA, the ERK signalling cascade was significantly downregulated by CHP, both in the WD/TN NAFLD and in the CCl_4-induced liver disease

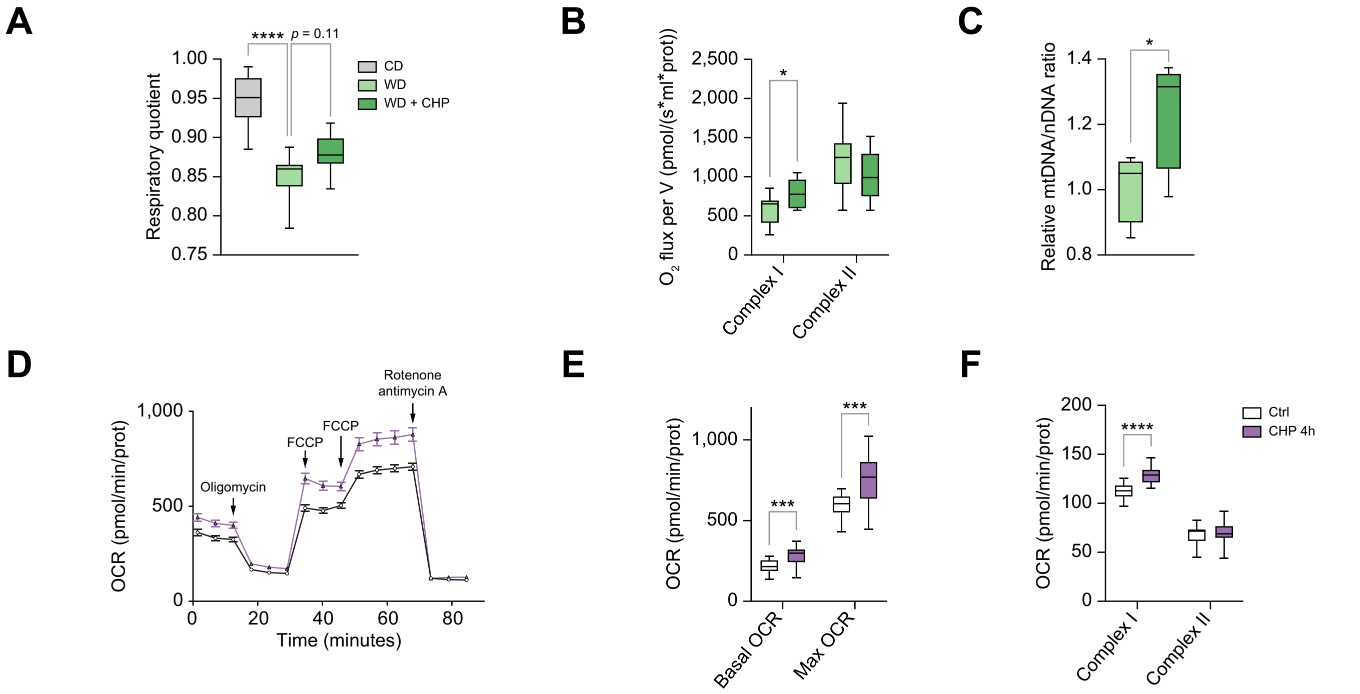

Fig. 7. CHP improves mitochondrial function in hepatocytes. (A) Respiratory quotient during the night, as measured at age 18 weeks (n = 7–8). (B) Complex I and II activity, normalised on protein content, in mitochondria isolated from fresh liver tissue (n = 7–8). (C) Relative mtDNA levels in mice receiving a WD, calculated as 16S mitochondrial gene expression normalised against hexokinase gene (n = 5). (D) Oxygen consumption of AML12 cells treated with 50 nM CHP for 4 h, when measuring respiration in basal, maximal, or leak state. OCR is normalised by protein content (n = 22–24). (E) Basal and maximal respiration as extracted from panel D. (F) Complex I and II activity in AML12 cells treated with 50 nM CHP (n = 23). One-way ANOVA, followed by Dunnett's multiple comparison test *vs.* WD group (A) or unpaired *t*-test (B, C, E, F) were used for statistical analysis. **p* <0.05; ****p* <0.001; *****p* <0.0001. AML12, alpha mouse liver 12; CHP, cyclo(His-Pro); FCCP, carbonyl cyanide-p-trifluoromethoxyphenylhydrazone; mtDNA, mitochondrial DNA; OCR, oxygen consumption rate; WD, western diet.

models (Fig. 8A left panels; S6A). Of note, this pathway was also over-represented in 2 RNAseq data sets collected from patients with NASH, when samples from patients with advanced NASH—with high NAS and fibrosis—are compared to earlier stages (Fig. 8A, right panel). Several upstream and downstream genes of the ERK pathway were differentially regulated in RNAseq data from the NAFLD study (Fig. 8B). Downregulation of the ERK signalling cascade was not limited to the liver tissue (Fig. S4C).

Protein expression of the phosphorylated (active) form of ERK1/2 (p-ERK1/2) in the livers at the end of the chronic feeding period of WD did not show any differences after CHP treatment (Fig. S6B). Therefore, a short-term experiment was performed in healthy male C57BL/6J mice, to monitor ERK1/2 phosphorylation at different time points after CHP treatment. Liver levels of p-ERK1/2 were already reduced 4 h after CHP treatment (Fig. 8C–E). Baseline p-ERK1/2 levels were restored after 24 h (Fig. S6C). To confirm this observation *in vitro*, AML-12 cells were treated with CHP for 4 or 24 h and protein levels were analysed by western blotting (Fig. 8F-H). The p-ERK1/2 levels were robustly reduced 4 h after CHP treatment, and at 24 h returned closer to baseline. Overall, these data indicated that CHP inhibits ERK1/2 in the short term, by reducing its phosphorylation and down-regulating its downstream pathways.

Discussion

In this study, we assessed the effects of CHP on the pathogenesis of NAFLD in a mouse model in which animals were challenged with WD feeding and TN housing.[41,42] Under WD, mice constantly gained weight over time, and several plasma parameters associated with increasing fat accumulation, including levels of LDL-cholesterol, glycaemia, and liver enzymes. Mice fed with WD developed enlarged livers, marked by broad steatosis, inflammation, and fibrosis, thus reproducing the main features of the NAFLD/NASH transition. CHP administration restored glucose homeostasis and prevented hepatic fat accumulation as seen by histological evaluation. Steatosis, inflammation, and fibrosis were all partially prevented by treatment, and the pathological evaluation indicated that CHP attenuated the progression of NASH.

As highlighted by our transcriptome analysis, CHP inhibited genes related to lipid metabolism, explaining the overall reduced fat accumulation observed in our murine models. Furthermore, CHP robustly downregulated pro-inflammatory pathways and reduced immune cell infiltration in the liver, supporting the histological findings. Reducing inflammation in NAFLD is crucial in breaking the vicious circle towards liver fibrosis[50,51] and eventually toward hepatocellular carcinoma.[52]

CHP also proved to be an effective preventive strategy in a second mouse model of liver disease, induced by repeated low-dose CCl_4 injections. The hepatotoxicity of CCl_4 was manifested in histology by extensive cellular hypertrophy and fibrosis. At the gene expression level, the effects of CHP were similar to those observed in the WD/TN model and also confirmed the attenuation of apoptosis and oxidative stress pathways. These 2 animal models of liver disease—induced by WD/TN or CCl_4—have different mechanisms of action leading to inflammation and fibrosis, nonetheless CHP proved to exert preventive effects in

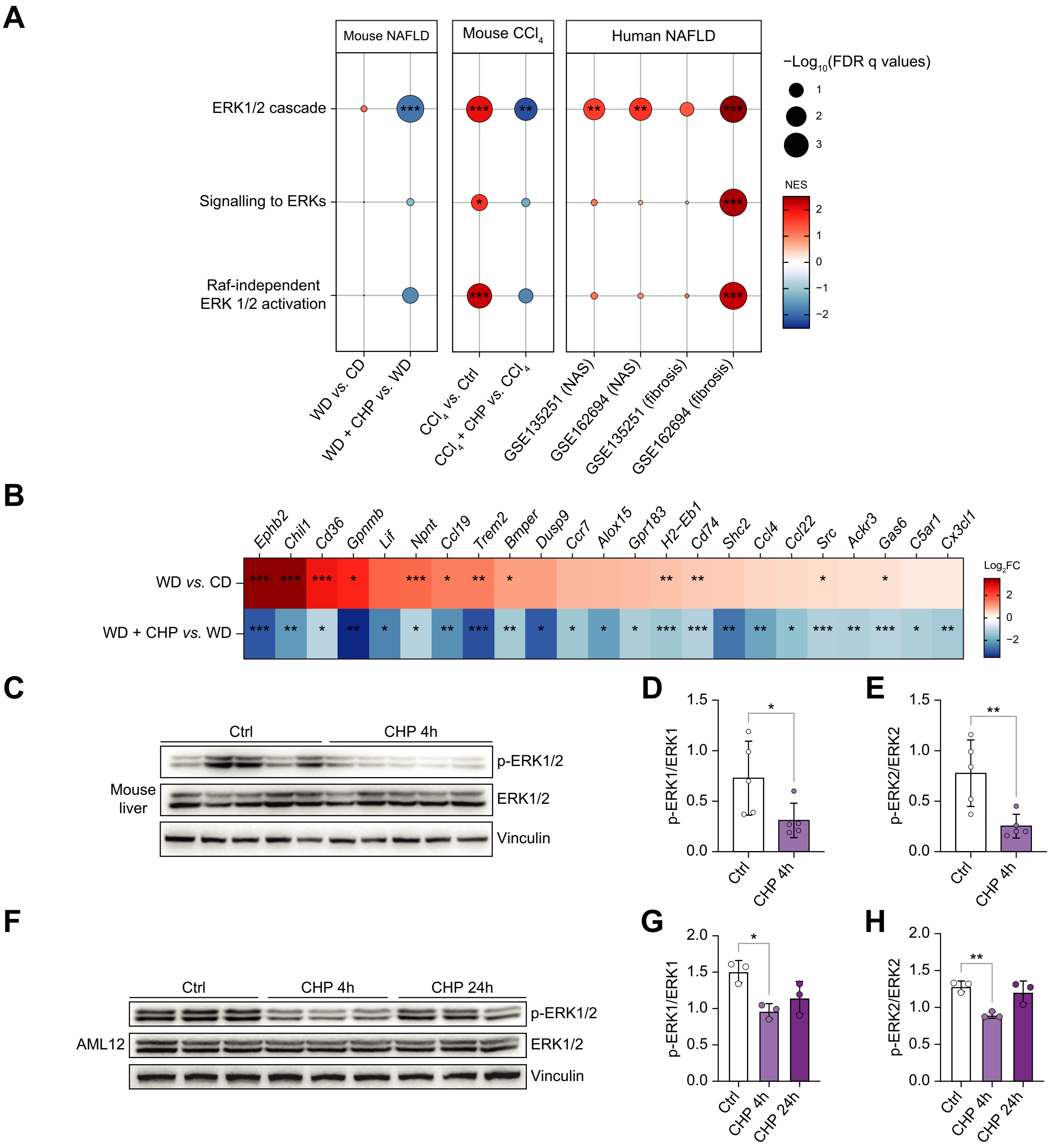

Fig. 8. CHP triggers an early downregulation of ERK signalling. (A) GSEA of the effects of disease (WD/CCl_4) and treatment (CHP) on gene expression in the mouse liver in this study (left panels), as well as that of the effects of disease on humans (NAS ≥4 *vs.* NAS <4 and fibrosis ≥3 *vs.* fibrosis <3) for ERK-related gene sets. (B) Heatmap shows the effects of WD and CHP on the expression of genes involved in ERK1/2 signalling. **p* <0.05; ***p* <0.01; ****p* <0.001 (adjusted *p* values). (C) Western blotting of phosphorylated and total ERK1/2 in the liver of mice treated with 20 mg/kg CHP for 4 h. Vinculin was used as the loading control. (D, E) Quantification of phosphorylated ERK1 (D) and ERK2 (E) signals from the western blotting in (C), normalised by total ERK1/2. Unpaired *t*-test was used for statistical analysis. (F) Western blotting of phosphorylated and total ERK1/2 in the AML12 cell line, treated with 100 nM CHP for 4 or 24 h. Vinculin was used as the loading control. (G, H) Quantification of phosphorylated ERK1 (G) and ERK2 (H) signals from the western blotting in (F), normalised by total ERK1/2. One-way ANOVA, followed by Dunnett's multiple comparison test *vs.* CTRL group was used for statistical analysis. Error bars in bar plots represent the standard deviation (D, E, G, H). **p* <0.05; ***p* <0.01. AML12, alpha mouse liver 12; ANOVA, analysis of variance; CCl_4, carbon tetrachloride; CHP, cyclo(His-Pro); CTRL, control; ERK, extracellular signal-regulated kinase; FC, fold change; FDR, false discovery rate; GSEA, gene set-enrichment analysis; NAFLD, non-alcoholic fatty liver disease; NAS, NAFLD activity score; NES, normalized enrichment score; WD, western diet.

both settings (Figs. 1A and 4A, Fig. S5A). To provide support for a therapeutic effect of CHP, a follow-up study tested the effects of CHP using a CCl_4 mouse model in which treatments were started at a later time point (Fig. 6A). Also in this setting, when CHP was administered as a treatment after symptoms of CCl_4-induced liver damage were evident, it was able to attenuate and blunt disease progression, providing a strong indication that CHP could be a candidate drug to manage NAFLD/NASH in the clinical setting.

The liver is a highly metabolic organ involved in the metabolism of all macronutrients. Several studies have underscored the link between mitochondrial function and NAFLD, and mitochondria have become an interesting therapeutic target.[53–56] Indeed, also in our studies, we observed that mitochondrial function was impaired by WD/TN challenge. Mice treated with CHP showed an improved mitochondrial phenotype *in vivo*, stimulating us to further study the effects of CHP on mitochondrial respiration *in vitro*. In mouse hepatocytes, CHP also increased cellular respiration, particularly the activity of complex I of the mitochondrial oxidative phosphorylation system. CHP effects on mitochondria were previously unknown, and thus, warrant further characterisation, not only in hepatocytes but also in other liver cell types. We speculate that the discrete changes in mitochondrial metabolism that we observed might not have a visible impact on overall energy expenditure, but may still play a role in maintaining hepatocyte function and glucose and lipid metabolism, contributing to the final beneficial phenotypic changes after CHP treatment.

Two human datasets collected from patients with NASH showed that ERK signalling is activated in patients with increasing severity of NAS and fibrosis stage. The ERK pathway has been associated with the development of NASH, as its activation plays a role in driving inflammation and fibrosis.[57,58] Inhibition of ERK1 has been associated with decreased proliferation of hepatic stellate cells, attenuated fibrosis, and improved lipid homeostasis.[59,60] ERK2 activity affects hepatocyte proliferation, playing also a role in inflammation and fibrosis upon liver injury.[61,62] Consistent with these studies, ERK signalling pathways were found to be upregulated in our liver transcriptome data, and CHP treatment downregulated ERK downstream genes. Our study showed that the direct effect of CHP on p-ERK1/2 levels in the liver was transient and could be detected after only 4 h, returning to baseline levels 24 h after CHP treatment. However, the effects on the downstream genes persisted longer, as shown in the transcriptome analysis. The same transient reduction of p-ERK1/2 levels with a nadir at 4 h was also observed *in vitro* in cultured AML12 hepatocytes. The inhibition of ERK activation after CHP was consistent in both *in vivo* and *in vitro* models, demonstrating for the first time a direct action of CHP on the ERK1/2 signalling pathway.

CHP was well tolerated in our long-term 17-week study in mice subjected to WD, as well as in a 39-week formal toxicology study performed in Beagle dogs (unpublished data). Furthermore, initial clinical studies have reported no safety concerns with CHP (NCT00878605; NCT02784275; NCT03560271). Its impeccable safety profile, together with the remarkable anti-inflammatory and antifibrotic effects of CHP in our study, point to CHP as a potential new strategy to prevent and manage NAFLD/NASH. Furthermore, our data implicate the downregulation of the ERK pathway as an early event after CHP treatment, although further work will be required to determine whether a direct or indirect inhibition by CHP is responsible for ERK regulation.

Overall, CHP appears to be promising candidate drug for the management of the transition from NAFLD to NASH. Furthermore, our data strongly suggest exploring the impact of CHP on additional diseases characterised by inflammation and fibrosis, such as chronic kidney disease or idiopathic pulmonary fibrosis.

Abbreviations

ACR, albumin-to-creatinine ratio; ALT, alanine transaminase; AML12, alpha mouse liver 12; ANOVA, analysis of variance; AST, aspartate transaminase; CCl_4, carbon tetrachloride; CD, chow diet; CHOL, cholesterol; CHP, cyclo(His-Pro); CRP, C-reactive peptide; DEA, differential expression analysis; EchoMRI, magnetic resonance imaging; EMT, epithelial–mesenchymal transition; ERK, extracellular signal-regulated kinase; FA, fatty acids; GAPDH, glyceraldehyde 3-phosphate dehydrogenase; GSEA, gene set-enrichment analysis; H&E, haematoxylin and eosin; HOMA, homeostatic model assessment; HSC, hepatic stellate cells; IL-6, interleukin 6; IR, insulin resistance; LDL, low-density lipoprotein; MRI, Magnetic resonance imaging; NAFLD, non-alcoholic fatty liver disease; NAS, NAFLD activity score; NASH, non-alcoholic steatohepatitis; NF-κB, nuclear factor kappa B; ORO, Oil Red O; PCA, principal component analysis; ROS, reactive oxygen species; SR, Sirius Red; α-SMA, alpha smooth muscle actin; TG, triglycerides; TGF-β, transforming growth factor beta; TN, thermoneutral housing; TNF-α, tumour necrosis factor alpha; TRH, thyrotropin-releasing hormone; WD, western diet.

Financial support

The work in the JA laboratory was supported by grants from the École Polytechnique Fédérale de Lausanne (EPFL), the European Research Council (ERC-AdG-787702), the Swiss National Science Foundation (SNSF 31003A_179435), the Global Research Laboratory (GRL) National Research Foundation of Korea (NRF 2017K1A1A2013124), and NovMetaPharma. ADM was funded by a European Innovative Training Networks H2020-MSCA-ITN-2018 (Healthage - 812830); QW was supported by the European Molecular Biology Organization (EMBO) postdoctoral fellowship (ALTF 111-2021); XL was supported by the China Scholarship Council (201906050019).

Conflicts of interest

This work was funded in part by a grant from NovMetaPharma. DL, JJ, SB, and OP are employees of NovMetaPharma. HYJ is a board member of NovMetaPharma. JA is a founder and/or consultant to MitoBridge/Astellas, Metro Biotech, Amazentis, Vandria, Orso Bio, and NovMetaPharma. All other authors declare that they have no competing interests.

Please refer to the accompanying ICMJE disclosure forms for further details.

Authors' contributions

The study was conceived by ADM, AM, HYJ, and JA. ADM, SB, and QW performed the *in vivo* experiments and the analysis of the experimental results. RNA sequencing analysis was carried out by XL and KS. In vitro experiments were performed by ADM, JJ, DL, OP, and QW. AM supported the organisation of the animal study. ADM and JA wrote the manuscript with contributions from all co-authors. JA and HYJ supervised the study and secured funding.

Data availability statement

The data supporting the findings are available upon request to the corresponding author (JA). RNA-seq data are available under the GEO numbers GSE200750, GSE216366 and GSE230745. Methods, materials, and resources are included in the Materials and methods or Supplementary Methods.

Acknowledgements

We thank L.E. Vogeleisen-Delpech, C.S. Mendes Ferreira, F. Fraga, S. Bichet, M.M. Janod, T. Clerc, and P. Stefanelli for their help with animal studies; M. Jasoria for helping with the analysis; and M. Bou Sleiman for the animal study figures. We also thank the EPFL histology facility and the mouse phenotyping unit (UDP) for their technical assistance. Finally, we are particularly grateful to all members of J. Auwerx's laboratory for helpful discussions.

Supplementary data

Supplementary data to this article can be found online at https://doi.org/10.1016/j.jhepr.2023.100815.

References

Author names in bold designate shared co-first authorship

[1] Bellentani S. The epidemiology of non-alcoholic fatty liver disease. Liver Int 2017;37:81–84.
[2] Chalasani N, Younossi Z, Lavine JE, Diehl AM, Brunt EM, Cusi K, et al. The diagnosis and management of non-alcoholic fatty liver disease: practice guideline by the American association for the study of liver diseases, American college of gastroenterology, and the American gastroenterological association. Hepatology 2012;55:2005–2023.
[3] Koenig AB, Abdelatif D, Fazel Y, Henry L, Wymer M. Global epidemiology of nonalcoholic fatty liver disease-Meta-analytic assessment of prevalence, incidence, and outcomes. Hepatology 2016;64:73–84.
[4] Byrne CD, Targher G. NAFLD: a multisystem disease. J Hepatol 2015;62(1 Suppl):S47–S64.
[5] Dufour JF, Scherer R, Balp MM, McKenna SJ, Janssens N, Lopez P, et al. The global epidemiology of nonalcoholic steatohepatitis (NASH) and associated risk factors–A targeted literature review. Endocr Metab Sci 2021;3:100089.
[6] Liu Q, Bengmark S, Qu S. The role of hepatic fat accumulation in pathogenesis of non-alcoholic fatty liver disease (NAFLD). Lipids Health Dis 2010;9:1–9.
[7] Ekstedt M, Franzén LE, Mathiesen UL, Thorelius L, Holmqvist M, Bodemar G, et al. Long-term follow-up of patients with NAFLD and elevated liver enzymes. Hepatology 2006;44:865–873.
[8] McPherson S, Hardy T, Henderson E, Burt AD, Day CP, Anstee QM. Evidence of NAFLD progression from steatosis to fibrosing-steatohepatitis using paired biopsies: implications for prognosis and clinical management. J Hepatol 2015;62:1148–1155.
[9] **Ekstedt M**, **Hagström H**, Nasr P, Fredrikson M, Stål P, Kechagias S, et al. Fibrosis stage is the strongest predictor for disease-specific mortality in NAFLD after up to 33 years of follow-up. Hepatology 2015;61:1547–1554.
[10] Anstee QM, Targher G, Day CP. Progression of NAFLD to diabetes mellitus, cardiovascular disease or cirrhosis. Nat Rev Gastroenterol Hepatol 2013;10:330–344.
[11] **Leoni S**, **Tovoli F**, **Napoli L**, **Serio I**, **Ferri S**, **Bolondi L**. Current guidelines for the management of non-alcoholic fatty liver disease: a systematic review with comparative analysis. World J Gastroenterol 2018;24:3361.
[12] Minelli A, Bellezza I, Grottelli S, Galli F. Focus on cyclo(His-Pro): history and perspectives as antioxidant peptide. Amino Acids 2008;35:283–289.
[13] Prasad C, Peterkofsky A. Demonstration of pyroglutamylpeptidase and amidase activities toward thyrotropin-releasing hormone in hamster hypothalamus extracts. J Biol Chem 1976;251:3229–3234.
[14] Prasad C. Bioactive cyclic dipeptides. Peptides 1995;16:151–164.
[15] Mizuma T, Masubuchi S, Awazu S. Intestinal absorption of stable cyclic dipeptides by the oligopeptide transporter in rat. J Pharm Pharmacol 1998;50:167–172.
[16] Banks WA. Characteristics of compounds that cross the blood-brain barrier. BMC Neurol 2009;9(Suppl 1):5–9.
[17] Jaspan JB, Banks WA, Kastin AJ. Study of passage of peptides across the blood-brain barrier: biological effects of cyclo(His-Pro) after intravenous and oral administration. Ann N Y Acad Sci 1994;739:101–107.
[18] Prasad C. Cyclo(His-Pro): its distribution, origin and function in the human. Neurosci Biobehav Rev 1988;12:19–22.
[19] Hilton CW, Prasad C, Vo P, Mouton C. Food contains the bioactive peptide, cyclo(His-Pro). J Clin Endocrinol Metab 1992;375–8.
[20] Uyemura K, Dhanani S, Yamaguchi DT, Song MK. Metabolism and toxicity of high doses of cyclo (His-Pro) plus zinc in healthy human subjects. J Drug Metab Toxicol 2010;1:1–9.
[21] Mizuma H, Legardeur BY, Prasad C, Hilton CW. The bioactive peptide cyclo(His-Pro) may be absorbed following ingestion of nutritional supplements that contain it. J Am Coll Nutr 1996;15:175–179.
[22] Puniak MA, Freeman GM, Agresta CA, Van Newkirk L, Barone CA, Salzman SK. Comparison of a serotonin antagonist, opioid antagonist, and TRH analog for the acute treatment of experimental spinal trauma. J Neurotrauma 1991;8:193–203.
[23] Faden AI, Knoblach SM, Movsesyan VA, Cernak I. Novel small peptides with neuroprotective and nootropic properties. J Alzheimers Dis 2004;6(Suppl):S93–S97.
[24] Faden AI, Movsesyan VA, Knoblach SM, Ahmed F, Cernak I. Neuroprotective effects of novel small peptides in vitro and after brain injury. Neuropharmacology 2005;49:410–424.
[25] Grottelli S, Ferrari I, Pietrini G, Peirce MJ, Minelli A, Bellezza I. The role of cyclo(His-Pro) in neurodegeneration. Int J Mol Sci 2016;17:1–14.
[26] Hwang IK, Go VL, Harris DM, Yip I, Kang KW, Song MK. Effects of cyclo (his-pro) plus zinc on glucose metabolism in genetically diabetic obese mice. Diabetes Obes Metab 2003;5:317–324.
[27] Song MK, Rosenthal MJ, Song AM, Yang H, Ao Y, Yamaguchi DT. Raw vegetable food containing high cyclo (his-pro) improved insulin sensitivity and body weight control. Metabolism 2005;54:1480–1489.
[28] Park Y, Lee HJ, Choi JW, Bae SH, Suh HJ. Anti-diabetic effect of Cyclo-His-Pro (CHP)-enriched yeast hydrolysate in streptozotocin-induced diabetic mice. Afr J Biotechnol 2013;12:5473–5479.
[29] Song MK, Hwang IK, Rosenthal MJ, Harris DM, Yamaguchi DT, Yip I, et al. Anti-hyperglycemic activity of zinc plus cyclo (his-pro) in genetically diabetic Goto-Kakizaki and aged rats. Exp Biol Med (Maywood) 2003;228:1338–1345.
[30] Minelli A, Conte C, Grottelli S, Bellezza I, Cacciatore I, Bolaños JP. Cyclo(-His-Pro) promotes cytoprotection by activating Nrf2-mediated upregulation of antioxidant defence. J Cell Mol Med 2009;13:1149–1161.
[31] Minelli A, Grottelli S, Mierla A, Pinnen F, Cacciatore I, Bellezza I. Cyclo(His-Pro) exerts anti-inflammatory effects by modulating NF-κB and Nrf2 signalling. Int J Biochem Cell Biol 2012;44:525–535.
[32] Argmann CA, Champy MF, Auwerx J. Evaluation of energy homeostasis. Curr Protoc Mol Biol 2006;73:29B.1.1–29B.1.17.
[33] Matthews DR, Hosker JP, Rudenski AS, Naylor BA, Treacher DF, Turner RC. Homeostasis model assessment: insulin resistance and beta-cell function from fasting plasma glucose and insulin concentrations in man. Diabetologia 1985;28:412–419. Diabetol 1985 287 ;28:412–419.
[34] **Chen Y**, **Chen Y**, **Shi C**, Huang Z, Zhang Y, Li S, et al. SOAPnuke: a MapReduce acceleration-supported software for integrated quality control and preprocessing of high-throughput sequencing data. Gigascience 2018;7:1–6.
[35] R: the R project for statistical computing. https://www.r-project.org/ (accessed 2001 Dec 17).
[36] LaMar D. 2015. FastQC, https://qubeshub.org/resources/fastqc.
[37] Andrews S. FastQC: A Quality Control Tool for High Throughput Sequence Data [Online]. http://www.bioinformatics.babraham.ac.uk/projects/fastqc/.
[38] Dobin A, Davis CA, Schlesinger F, Drenkow J, Zaleski C, Jha S, et al. STAR: ultrafast universal RNA-seq aligner. Bioinformatics 2013;29:15–21.
[39] Law CW, Chen Y, Shi W, Smyth GK. Voom: precision weights unlock linear model analysis tools for RNA-seq read counts. Genome Biol 2014;3(15):R29.
[40] Ritchie ME, Phipson B, Wu D, Hu Y, Law CW, Shi W, et al. Limma powers differential expression analyses for RNA-sequencing and microarray studies. Nucleic Acids Res 2015;43(7):e47.
[41] Giles DA, Moreno-Fernandez ME, Stankiewicz TE, Graspeuntner S, Cappelletti M, Wu D, et al. Thermoneutral housing exacerbates nonalcoholic fatty liver disease in mice and allows for sex-independent disease modeling. Nat Med 2017;23:829–838.
[42] Benegiamo G, von Alvensleben GVG, Rodríguez-López S, Goeminne LJE, Bachmann AM, et al. The genetic background shapes the susceptibility to mitochondrial dysfunction and NASH progression. J Exp Med 2023;220(4):e20221738.
[43] Utzschneider KM, Kahn SE. Review: the role of insulin resistance in nonalcoholic fatty liver disease. J Clin Endocrinol Metab 2006;91:4753–4761.
[44] Santoleri D, Titchenell PM. Resolving the paradox of hepatic insulin resistance. Cell Mol Gastroenterol Hepatol 2019;7:447–456.
[45] Byrne CD, Targher G. NAFLD as a driver of chronic kidney disease. J Hepatol 2020;72:785–801.
[46] Linge J, Nasr P, Sanyal AJ, Dahlqvist Leinhard O, Ekstedt M. Adverse muscle composition is a significant risk factor for all-cause mortality in NAFLD. JHEP Rep 2022;5:100663.
[47] **Nachit M**, **Kwanten WJ**, Thissen JP, Op De Beeck B, Van Gaal L, et al. Muscle fat content is strongly associated with NASH: a longitudinal study in patients with morbid obesity. J Hepatol 2021;75:292–301.

[48] Korenaga K, Korenaga M, Teramoto F, Suzuki T, Nishina S, Sasaki K, et al. Clinical usefulness of non-protein respiratory quotient measurement in non-alcoholic fatty liver disease. Hepatol Res 2013;43:1284–1294.

[49] Tajika M, Kato M, Mohri H, Miwa Y, Kato T, Ohnishi H, et al. Prognostic value of energy metabolism in patients with viral liver cirrhosis. Nutrition 2002;18:229–234.

[50] Sutti S, Albano E. Adaptive immunity: an emerging player in the progression of NAFLD. Nat Rev Gastroenterol Hepatol 2019;72(17):81–92.

[51] Albhaisi S, Noureddin M. Current and potential therapies targeting inflammation in NASH. Front Endocrinol (Lausanne) 2021;12:767314.

[52] **Anstee QM**, **Reeves HL**, **Kotsiliti E**, Govaere O, Heikenwalder M. From NASH to HCC: current concepts and future challenges. Nat Rev Gastroenterol Hepatol 2019;167:411–428.

[53] **Gariani K**, **Menzies KJ**, Ryu D, Wegner CJ, Wang X, Ropelle ER, et al. Eliciting the mitochondrial unfolded protein response by nicotinamide adenine dinucleotide repletion reverses fatty liver disease in mice. Hepatology 2016;63:1190–1204.

[54] Katsyuba E, Mottis A, Zietak M, De Franco F, van der Velpen V, Gariani K, et al. De novo NAD+ synthesis enhances mitochondrial function and improves health. Nature 2018;563:354–359.

[55] **Bresciani N**, **Demagny H**, Lemos V, Pontanari F, Li X, Sun Y, et al. The Slc25a47 locus is a novel determinant of hepatic mitochondrial function implicated in liver fibrosis. J Hepatol 2022;77:1071–1082.

[56] Di Ciaula A, Passarella S, Shanmugam H, Noviello M, Bonfrate L, Wang DQ, et al. Nonalcoholic fatty liver disease (NAFLD). Mitochondria as players and targets of therapies? Int J Mol Sci 2021;22:5375.

[57] Foglia B, Cannito S, Bocca C, Parola M, Novo E. ERK pathway in activated, myofibroblast-like, hepatic stellate cells: a critical signaling crossroad sustaining liver fibrosis. Int J Mol Sci 2019;20:2700.

[58] Alshehade S, Alshawsh MA, Murugaiyah V, Asif M, Alshehade O, Almoustafa H, Al Zarzour RH. The role of protein kinases as key drivers of metabolic dysfunction-associated fatty liver disease progression: new insights and future directions. Life Sci 2022;305:120732.

[59] Zhong W, Shen WF, Ning BF, Hu PF, Lin Y, Yue HY, et al. Inhibition of extracellular signal-regulated kinase 1 by adenovirus mediated small interfering RNA attenuates hepatic fibrosis in rats. Hepatology 2009;50:1524–1536.

[60] Bi L, Chiang JY, Ding WX, Dunn W, Roberts B, Li T. Saturated fatty acids activate ERK signaling to downregulate hepatic sortilin 1 in obese and diabetic mice. J Lipid Res 2013;54:2754–2762.

[61] Jeng KS, Lu SJ, Wang CH, Chang CF. Liver fibrosis and inflammation under the control of ERK2. Int J Mol Sci 2020;21:3796.

[62] **Frémin C**, **Ezan F**, Boisselier P, Bessard A, Pagès G, Pouysségur J, et al. ERK2 but not ERK1 plays a key role in hepatocyte replication: an RNAi-mediated ERK2 knockdown approach in wild-type and ERK1 null hepatocytes. Hepatology 2007;45:1035–1045.

Journal of Hepatology, Volume *5*

Supplemental information

Cyclo(His-Pro): A further step in the management of steatohepatitis

Alessia De Masi, Xiaoxu Li, Dohyun Lee, Jongsu Jeon, Qi Wang, Seoyeong Baek, Onyu Park, Adrienne Mottis, Keno Strotjohann, Alexis Rapin, Hoe-Yune Jung, and Johan Auwerx

Cyclo (His-Pro): a further step in the management of steatohepatitis

Alessia De Masi[1], Xiaoxu Li[1], Dohyun Lee[2], Jongsu Jeon[2], Qi Wang[1], Seoyeong Baek[2], Onyu Park[2], Adrienne Mottis[1], Keno Strotjohann[1], Alexis Rapin[1], Hoe-Yune Jung[2,3,]*, Johan Auwerx[1,]*.

[1] Laboratory of Integrative Systems Physiology, Institute of Bioengineering, École Polytechnique Fédérale de Lausanne, Lausanne 1015, Switzerland

[2] R&D Center, NovMetaPharma Co., Ltd., Pohang, 37668, South Korea

[3] School of Interdisciplinary Bioscience and Bioengineering, Pohang University of Science and Technology (POSTECH), Pohang, 37673, South Korea

* Corresponding authors:

Johan Auwerx - Address: Laboratory of Integrative and Systems Physiology, Ecole Polytechnique Fédérale de Lausanne, CH-1015 Lausanne, Switzerland. Tel.: +41 216939522. E-mail address: admin.auwerx@epfl.ch

Hoe-Yune Jung – Address: R&D Center, NovMetaPharma Co., Ltd., Pohang, 37668, South Korea. Tel.: +82 (0)54 223 2893. E-mail address: elijah98@novmeta.com

Table of contents

Supplementary methods 3
Supplementary figures 7
Supplementary tables 12
Supplementary references 14

Supplementary methods

Plasma biochemistry. For plasmatic biochemistry, blood samples were collected by intracardiac puncture under anesthesia, plasma was separated and stored at -80°C. Plasma parameters were measured using Dimension®Xpand Plus (Siemens Healthcare Diagnostics AG). Liver enzymes were assayed to assess a possible liver damage state. LDL-cholesterol levels were measured. The biochemical tests were performed according to the manufacturer kit for each parameter: ALT, AST, LDL (Siemens Healthcare). CRP concentration was measured using Mouse CRP ELISA Kit (Crystal Chem). Plasma IL-6 and TNFα were measured with ELISA Kits (Invitrogen).

Urine biochemistry. Urine was collected the day before euthanasia and stored at -80°C. Creatinine and micro-albumin were measured using AU480 Clinical Chemistry System (Beckman Coulter) following manufacturer's protocols.

Liver characterization. Macroscopic pictures of livers were taken after PBS (Gibco) perfusion. Liver TG levels were measured with TRIGL kit (c111, Cobas, Roche). CHOL levels were measured with CHOL2 kit (c111, Cobas, Roche). To measure the activity of mitochondrial complexes in mouse liver, mitochondria were isolated from fresh whole liver tissue as previously described [1]. Pellets of mitochondrial were quantified for proteins, equalized and resuspended in MiR05 (Oroboros Instruments). Oxygen consumption rate (OCR) was assessed by high-resolution respirometry (Oxygraph 2k, Oroboros Instruments) according to the manufacturer's protocol. Compounds were added directly into the 2 ml chamber: pyruvate (5 mM), malate (2 mM), glutamate (10 mM), ADP+Mg^{2+} (1.25 mM) for complex I; succinate (10 mM) for complex II; inhibitors for complex I (rotenone 0.5 μM) and complex II (antimycin A, 2.5 μM); all compounds are from Sigma. Mitochondrial content was measured by the relative mtDNA/nDNA ratio as previously described [2].

Histology. For histological analysis, liver samples were taken from the same lobe of each animal. 4 μm paraffin sections were processed with standard H&E staining to assess the general morphology, and Sirius red F3B (SR) or Direct Red + Fast Green FCF as counterstaining (Sigma) to highlight collagen fibers. Detection of CD45 positive immune cells was performed with ChromoMap DAB kit (Roche Diagnostics). 8 μm cryosections sections were processed with standard ORO protocol to detect lipids. Images were taken with an Olympus Slide Scanner VS120 L100 at 40x magnification. Digital slides were analyzed using QuPath software [3]. Stained liver tissue was quantified taking 4 random 8x fields on each slide, using 4 slides per experimental group; signal was quantified using ImageJ-Fiji software [4]. The histopathological assessment was performed in a blinded fashion by a board-certified veterinary pathologist (DECVP).

RNA-seq. The differentially expressed genes (DEGs) are defined by a Benjamini–Hochberg adjusted *P* value lower than 0.05 and an absolute $\log_2$[fold change] value higher than 1. GSEA was performed from fold-change sorted genes using clusterProfiler R package (version 3.10.1) [5], using gene sets retrieved with the msigdbr R package (version 7.2.1) [6]–[8]. 11 additional custom gene sets related to HSC were obtained from GO terms (2000490, 2000491) and literature [9]–[13] (Table S2). The gene sets with absolute normalized enrichment score (NES) higher than 1 and false discovery rate (qValue) lower than 0.05 are identified as significantly enriched gene sets.

The gene markers for 29 cell types in liver were retrieved from the supplementary material of two single cell RNA sequencing (scRNA-seq) studies and calculated using ClusterProfiler [5], [14], [15].

Cell type deconvolution analysis was performed with the using MuSiC R package (version 0.2.0), with the liver scRNA-seq dataset [16] as a reference. Cell types were summarized

into three categories: endothelial cells (endothelial cell of hepatic sinusoid), hepatocytes and immune cells (B cell, Kupffer cell, Natural Killer cell).

The effect of increasing severity of NAS and fibrosis stages was measured in two human NAFLD datasets (GSE135251 [17] and GSE162694 [18]). We compared NAS 4-8 versus NAS 0-3 and Fibrosis 3-4 versus Fibrosis 0-3 using the limma R package (version 3.38.3) with sex as a covariate, then performed GSEA using the clusterProfiler package.

RNA extraction, cDNA synthesis, and Real-time PCR. Total RNA was extracted from the liver using NucleoZOL reagent (Macherey-Nagel). 1µg of total RNA was used for cDNA synthesis using iScript cDNA synthesis kit (Bio-rad). Real-time qPCR (RT-qPCR) was performed using IQ SYBR® Green Supermix (Bio-Rad). The gene expression level was normalized to *Gapdh* gene. Specific primer pairs are listed in table S3.

Cellular respiration. Cellular respiration was assessed in AML12 cells treated with 50 nM CHP for 4 hours. Oxygen consumption rate was measured with the Seahorse XF96 instrument (Agilent), according to the manufacturer's protocol. Compounds were injected in the wells during measurement to assess basal and maximal respiration: oligomycin (0.1 µM), carbonyl cyanide-p-trifluoromethoxyphenylhydrazone (FCCP, 1 µM), rotenone (1 µM), antimycin A (1 µM). All compounds are from Sigma. To measure the specific activity of mitochondrial complexes, cells were permeabilized with 7.5 µg/ml digitonin and standard protocol from manufacturer (Agilent) was followed.

Western blot. Proteins were extracted in RIPA buffer, and Laemmi buffer was added for loading. Samples were loaded on 8% acrylamide sodium dodecyl sulfate-polyacrylamide gel (SDS-PAGE), then proteins were transferred onto polyvinylidene fluoride (PVDF) membranes (Immobilon-P PVDF Membrane, Millipore). Membranes were blocked with 5% skim milk-TBST, and incubated with primary antibodies overnight. Secondary antibody detection reactions were developed by enhanced chemiluminescence (SuperSignal West

Pico PLUS Chemiluminescent Substrate, Thermo Scientific) and imaged using the Fusion FX imaging system (Vilber). Quantification was performed using ImageJ software.

Antibodies. For histology, CD45 antibody (rat α-CD45, Thermo Fisher) was used. For western blotting, the following primary antibodies were used: phospho-ERK1/2 (rabbit α-phospho-p44/42 MAPK, Cell signaling), ERK1/2 (rabbit α-p44/42 MAPK, Cell signaling), Vinculin (rabbit recombinant α-vinculin, Abcam), αSMA (rabbit α-αSMA, Cell signaling), fibronectin (rabbit α-fibronectin, abcam).

Figures. BioRender was used to draw the animal studies outline (Fig. 1A, 4A, 6A, S5A) and the graphical abstract. Time-course, boxplots and barplots were created with GraphPad Prism 9.5.1. ImageJ software was used to prepare the western blot images. Adobe Illustrator 26.0.1 was used to assemble figure panels.

Supplementary figures

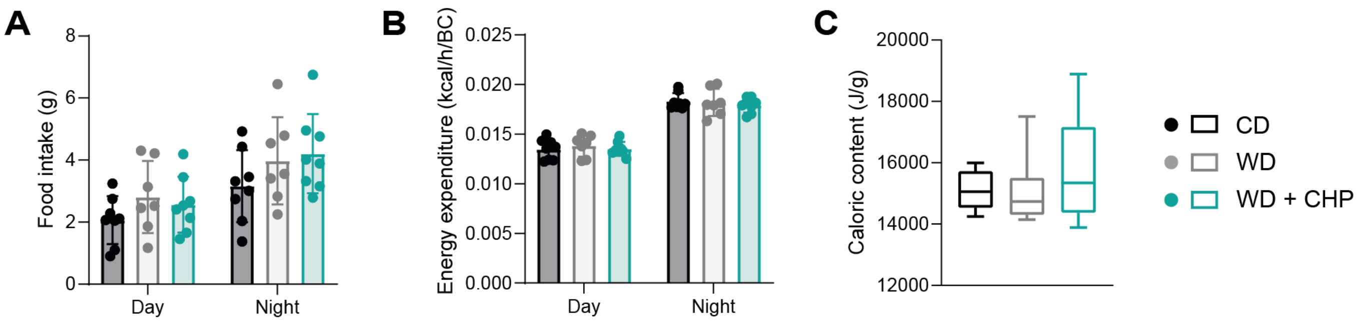

Fig. S1. CHP effect on metabolism. (A) Total food intake during day and night time. (B) Average hourly energy expenditure normalized on body composition (BC), considering both lean and fat mass. n=7-8. Results represent the mean ± standard deviation. (C) Fecal calorie content measured on feces collected over a 24-hour period, after 8 weeks of CD or WD and treatment with CHP. Whiskers in boxplots represent the min to max range. n=9-10.

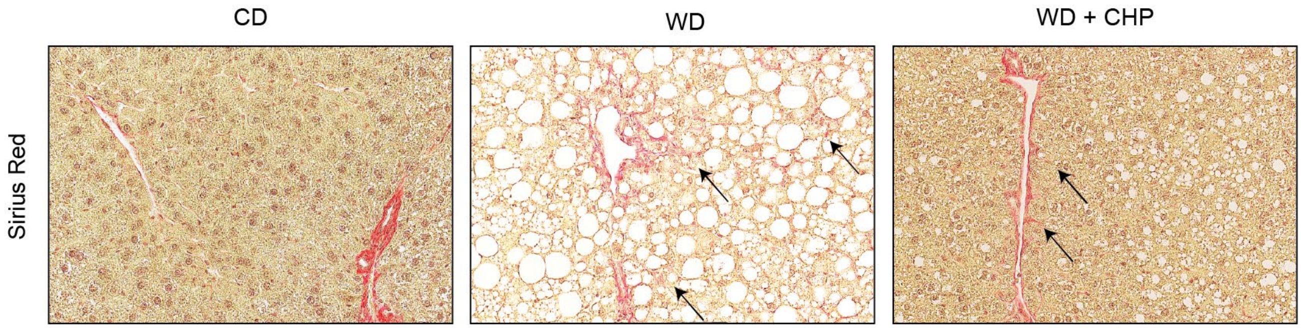

Fig. S2. CHP reduced the extension of fibrosis in mice fed with WD. Representative images of liver sections stained Sirius Red. Arrows indicate the extension of the collagen strands from the vessel to the periportal and midzonal area.

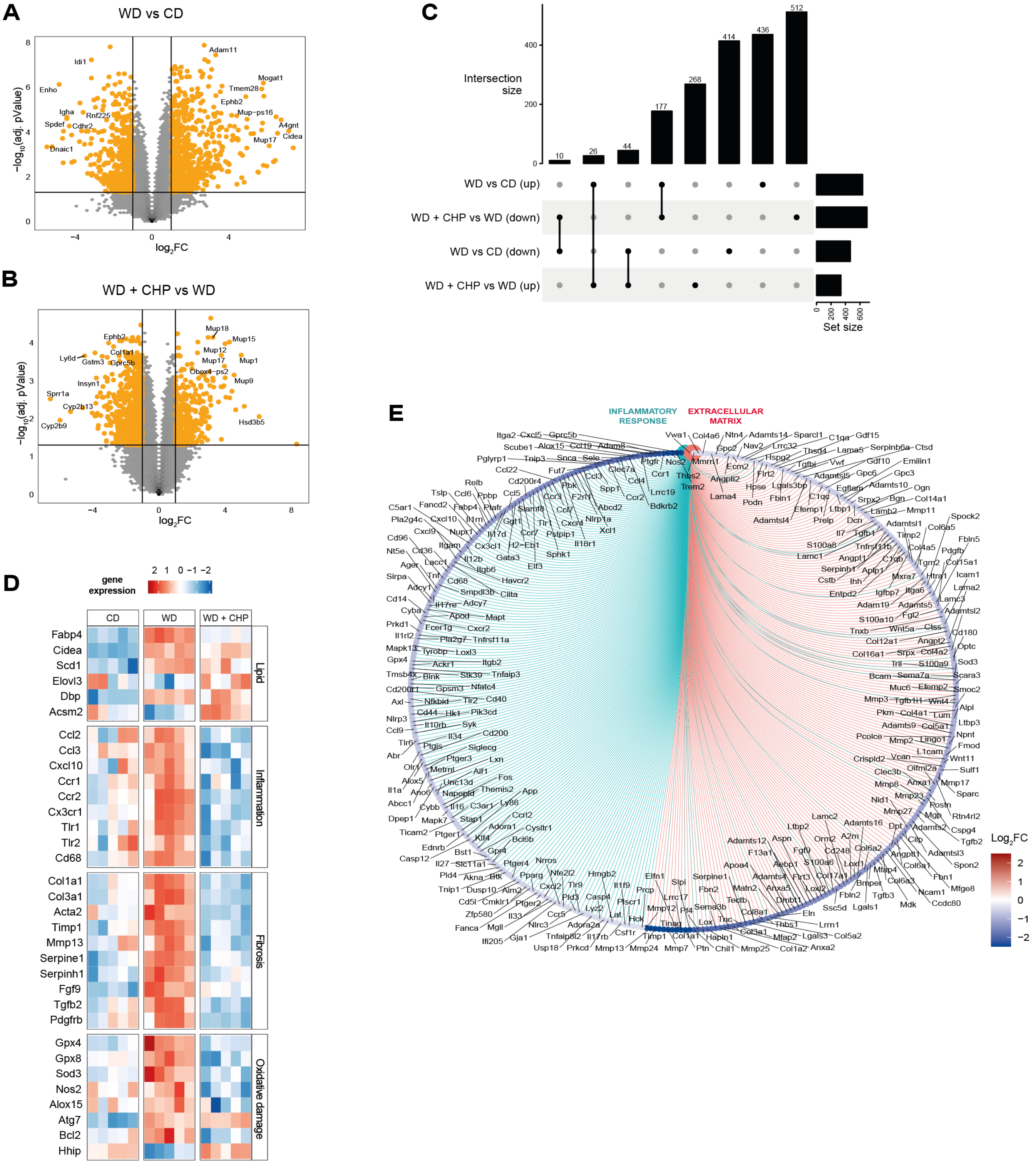

Fig. S3. Transcriptomic signatures of WD/TN and CHP. (A) Volcano plot showing the effect of WD on gene expression compared to the baseline condition (CD). (B) Volcano plot showing the effect of WD + CHP on gene expression compared to WD. The differentially expressed genes (|log2FC| >1 and adjusted *P*-value < 0.05) are highlighted in orange (A, B). (C) Upset Plot showing the exclusive intersections for the significantly differential expressed genes between comparisons. (D) Heatmap showing the effects of WD and CHP for given genes, grouped following the same four categories as in Fig.3C. (E) Gene-concept network

(cnet) plot showing the core enriched genes in inflammation and extracellular matrix gene sets for the effect of CHP.

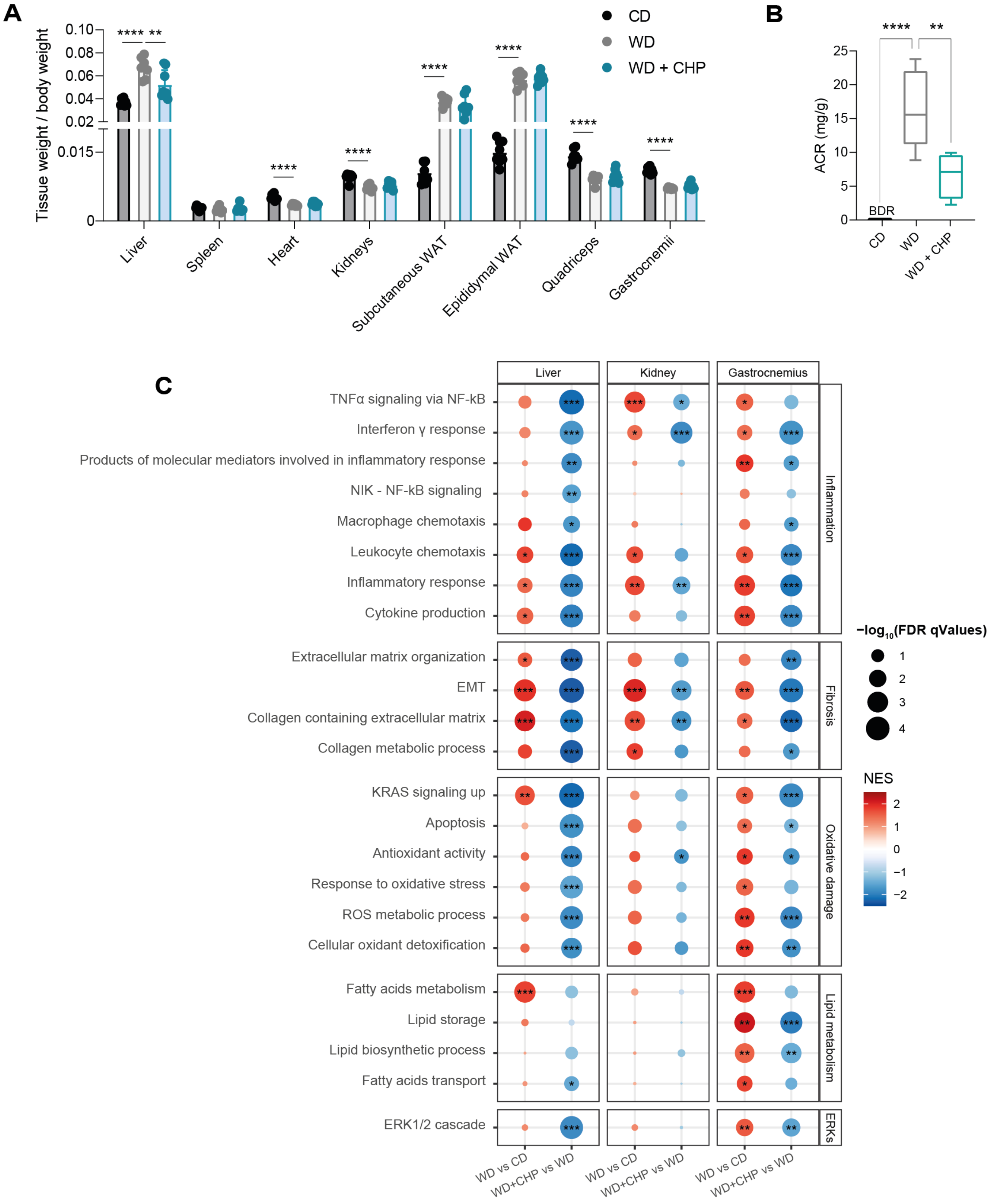

Fig. S4. Effects of WD feeding and CHP treatment on extrahepatic organs. (A) Comparison of organ size between the three experimental groups. n=7-8. (B) Albumin to creatinine ratio (ACR) in urine, expressed as mg of albumin to g of creatinine. BDR: below

detection range. n=5-7. One-way ANOVA, followed by Dunnett's multiple comparison test versus WD group was used for statistical analysis. Error bars in barplots represent the standard deviation; whiskers in boxplots represent min to max range. *P* values are indicated as follows: ** $P<0.01$; **** $P<0.0001$. (C) Gene set enrichment analysis of disease (WD) and treatment (CHP) effects on gene expression, analyzed across three tissues (liver, kidney, gastrocnemius). Gene sets are grouped in five categories: Inflammation, Fibrosis, Oxidative damage, Lipid metabolism, ERKs. *Q* values are indicated as follows: * $Q<0.05$; ** $Q<0.01$; *** $Q<0.001$.

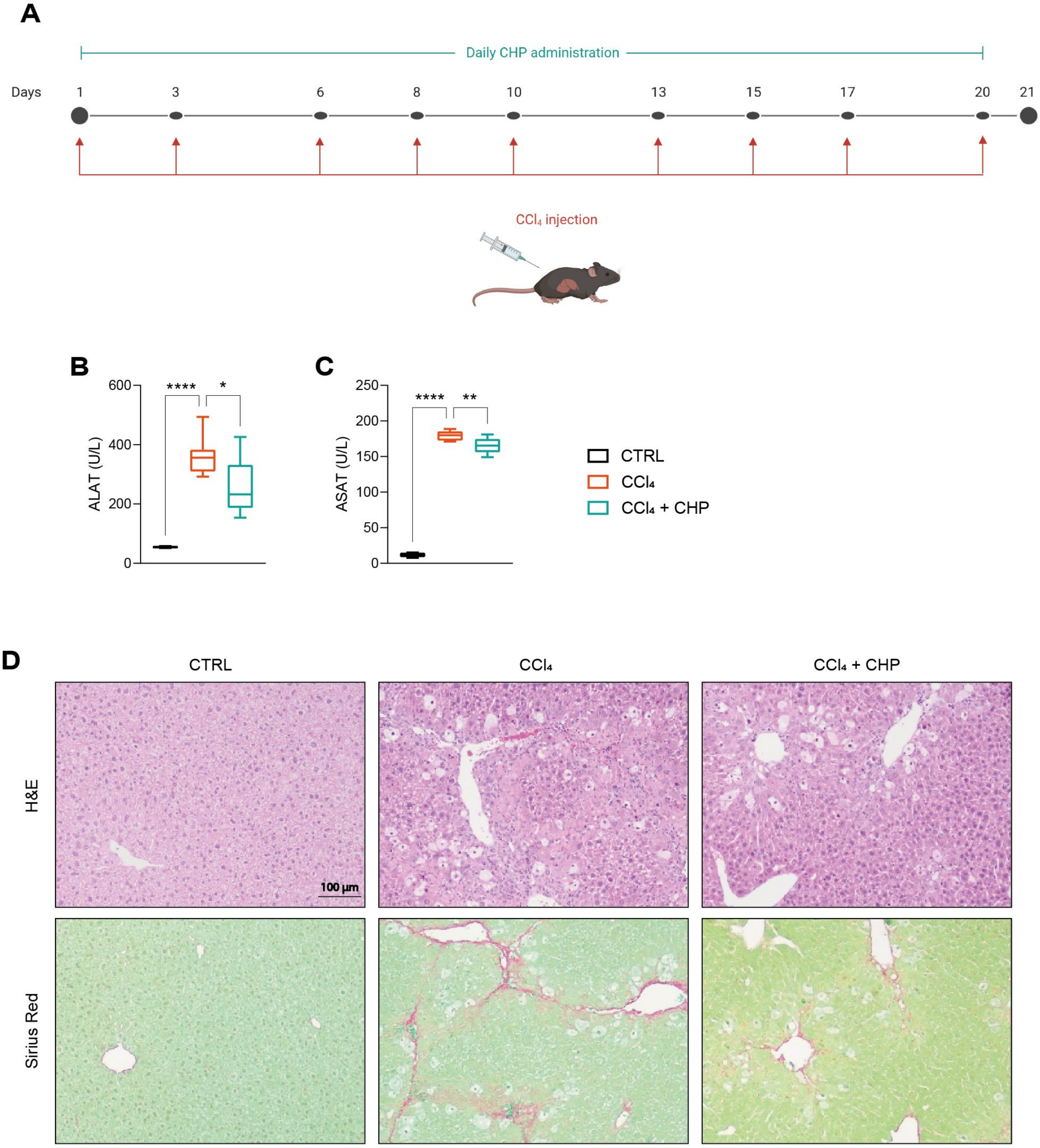

Fig. S5. CHP attenuated fibrosis and inflammation in a CCl_4-induced liver injury model. (A) Animal study outline. Mice received 9 injections of CCl_4 over 20 days, and were

treated with CHP daily. Liver and plasma were collected at day 21. (B-C) ALAT (B) and ASAT (C) plasma levels. Whiskers in boxplots represent the min to max range. (D) Representative images of liver sections stained with H&E or Sirius Red. n=4-7. One-way ANOVA, followed by Dunnett's multiple comparison test versus CCl_4 group was used for statistical analysis (B, C). *P* values are indicated as follows: * *P*<0.05; ** *P*<0.01; **** *P*<0.0001.

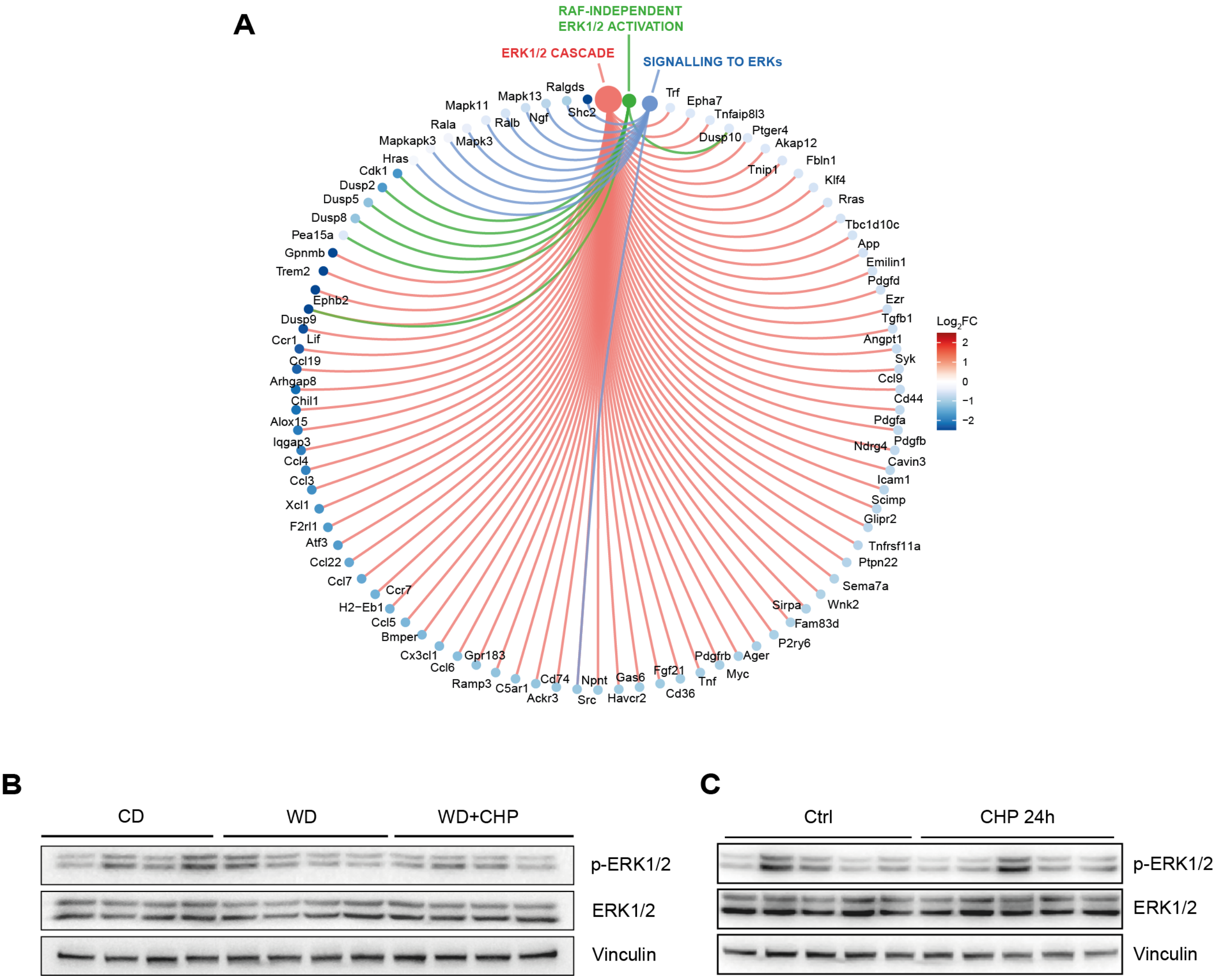

Fig. S6. ERK signaling in response to CHP treatment. (A) Gene-concept network (cnet) plot shows the core enriched genes in ERK signaling gene sets for the effect of CHP, in the WD/TN NAFLD model. (B) Western blot of phosphorylated and total ERK 1/2 in the liver of mice from the NASH study. Vinculin was used as loading control. (C) Western blot of phosphorylated and total ERK 1/2 in the liver of mice treated with 20 mg/kg CHP for 24 hours. Vinculin was used as loading control.

Supplementary tables

Table S1. Incidence and severity of remarkable histopathological findings in liver of mice subjected to CCl_4 injections.

		CTRL	CCl_4	CCl_4+CHP
**Number of animals**		2	7	7
**HE stain**				
Hyperplasia, oval cells	+	0	7	7
Hypertrophy, hepatocellular, centrilobular	++	0	7	7
Necrosis, hepatocellular, centrilobular	+<br>++<br>+++	0<br>0<br>0	0<br>0<br>7	4<br>3<br>0
**SR stain**				
Increased stain, centrilobular	+	0	7	0

Grade: + minimal, ++ mild, +++ moderate, ++++ marked

Table S2. 11 custom gene sets related to HSCs.

Gene set	Gene name	Ref.
HSC_activation_marker	Timp1	doi: 10.1038/s41598-019-39112-6
	Spp1	
	Mmp3	
	Gas6	
	Acta2	
	Col1a1	
	Col3a1	
	Col5a2	
	S100a6	doi: 10.3390/cells8050503
	saa3	
	Lox	doi: 10.1038/s41598-019-39112-6
	Lrat	
	Mfap4	doi: 10.1002/hep.31215
	Col1a2	
	Dpt	
HSC_proliferation	Egr1	doi: 10.1038/s41598-019-39112-6
	Ccnd1	
	Top2a	
	Cenpe	
	Rrm2	
Positive_regulation_of_HSC_activation_1	Lep	GO:2000491
	Dgat1	
	Acta2	

	Fgfr1	
	Pdgfrb	
	Pdgfb	
	Rps6ka1	
	Myocd	
Positive_regulation_of_HSC_activation_2	cdh11	doi: 10.1371/journal.pone.0233702
	cthrc1	
	fmod	
	prrx1	
	mfap4	
	pcdh15	
	ptprt	
	hpca	
Negative_regulation_of_HSC_activation	Gclc	GO:2000490
	Rian	
	Cygb	
	Gsk3b	
	Hhip	doi: 10.1371/journal.pone.0233702
Resting_HSC_marker	Fcna	doi: 10.3390/cells8050503
	Angptl6	
	Colec11	
	Tmem56	
	Plvap	
	Pth1a	
MFB1	Acta2	
	Tagln	
	Col1a1	
	Col6a3	
	Tpm1	
MFB2	Slpi	
	Saa3	
	c3	
	dmkn	
	cd74	
MFB3	Jund	
	Fosb	
	Egr1	
	Klf2	
MFB4	Mgp	
	Fbln	
	Meg3	
	Gas6	
	Hp	
MFB_1_TO_4	Acta2	
	Tagln	
	Col1a1	
	Col6a3	

	Tpm1	
	Slpi	
	Saa3	
	c3	
	dmkn	
	cd74	
	Jund	
	Fosb	
	Egr1	
	Klf2	
	Mgp	
	Fbln	
	Meg3	
	Gas6	
	Hp	

Table S3. Primer sets for Real-time PCR

	Forward (5'-3')	Reverse (5'-3')
Bcl-xL	TCTGAATGACCACCTAGAGCC	GCTGCATTGTTCCCGTAGAG
Puma	ACCTCAACGCGCAGTACG	GTAGGCACCTAGTTGGGCTC
HO-1	TATGCCCCACTCTACTTCCC	AGTGAGGCCCATACCAGAAG
Collagen I	GCCTCAGAAGAACTGGTACAT	ATCCATCGGTCATGCTCTCT
Collagen III	AGTCAAGGAGAAAGTGGTCG	CCAGGGAAACCCATGACAC
Collagen IV	CGGTACACAGTCAGACCATT	CATCACGAAGGAATAGCCGA
PAI-1	GTCTTTCCGACCAAGAGCAG	GCCGAACCACAAAGAGAAAG
TGF-β	TGATACGCCTGAGTGGCTGTCT	CACAAGAGCAGTGAGCGCTGAA
Gapdh	CAGTATGACTCCACCCACGG	ATGGGCTTCCCGTTGATGAC

Supplementary references

[1] C. Frezza, S. Cipolat, and L. Scorrano, “Organelle isolation: functional mitochondria from mouse liver, muscle and cultured filroblasts,” *Nat. Protoc. 2007 22*, vol. 2, no. 2, pp. 287–295, Feb. 2007, doi: 10.1038/nprot.2006.478.

[2] P. M. Quiros, A. Goyal, P. Jha, and J. Auwerx, “Analysis of mtDNA/nDNA ratio in mice,” *Curr. Protoc. Mouse Biol.*, vol. 7, no. 1, p. 47, Mar. 2017, doi: 10.1002/CPMO.21.

[3] P. Bankhead *et al.*, “QuPath: Open source software for digital pathology image analysis,” *Sci. Reports 2017 71*, vol. 7, no. 1, pp. 1–7, Dec. 2017, doi: 10.1038/s41598-017-17204-5.

[4] J. Schindelin *et al.*, “Fiji: an open-source platform for biological-image analysis,” *Nat. Methods 2012 97*, vol. 9, no. 7, pp. 676–682, Jun. 2012, doi: 10.1038/nmeth.2019.

[5] G. Yu, L. G. Wang, Y. Han, and Q. Y. He, “clusterProfiler: an R package for

comparing biological themes among gene clusters," *OMICS*, vol. 16, no. 5, pp. 284–287, May 2012, doi: 10.1089/OMI.2011.0118.

[6] A. Liberzon, A. Subramanian, R. Pinchback, H. Thorvaldsdóttir, P. Tamayo, and J. P. Mesirov, "Molecular signatures database (MSigDB) 3.0," *Bioinformatics*, vol. 27, no. 12, pp. 1739–1740, Jun. 2011, doi: 10.1093/BIOINFORMATICS/BTR260.

[7] A. Subramanian *et al.*, "Gene set enrichment analysis: A knowledge-based approach for interpreting genome-wide expression profiles," *Proc. Natl. Acad. Sci.*, vol. 102, no. 43, pp. 15545–15550, Oct. 2005, doi: 10.1073/PNAS.0506580102.

[8] A. Liberzon, C. Birger, H. Thorvaldsdóttir, M. Ghandi, J. P. Mesirov, and P. Tamayo, "The Molecular Signatures Database (MSigDB) hallmark gene set collection," *Cell Syst.*, vol. 1, no. 6, pp. 417–425, Dec. 2015, doi: 10.1016/J.CELS.2015.12.004.

[9] L. He, H. Yuan, J. Liang, J. Hong, and C. Qu, "Expression of hepatic stellate cell activation-related genes in HBV-, HCV-, and nonalcoholic fatty liver disease-associated fibrosis," *PLoS One*, vol. 15, no. 5, May 2020, doi: 10.1371/JOURNAL.PONE.0233702.

[10] A. B. Marcher *et al.*, "Transcriptional regulation of Hepatic Stellate Cell activation in NASH," *Sci. Reports 2019 91*, vol. 9, no. 1, pp. 1–13, Feb. 2019, doi: 10.1038/s41598-019-39112-6.

[11] M. K. Terkelsen *et al.*, "Transcriptional Dynamics of Hepatic Sinusoid-Associated Cells After Liver Injury," *Hepatology*, vol. 72, no. 6, pp. 2119–2133, Dec. 2020, doi: 10.1002/HEP.31215.

[12] O. Krenkel, J. Hundertmark, T. P. Ritz, R. Weiskirchen, and F. Tacke, "Single Cell RNA Sequencing Identifies Subsets of Hepatic Stellate Cells and Myofibroblasts in Liver Fibrosis," *Cells 2019, Vol. 8, Page 503*, vol. 8, no. 5, p. 503, May 2019, doi: 10.3390/CELLS8050503.

[13] I. Mederacke, D. H. Dapito, S. Affò, H. Uchinami, and R. F. Schwabe, "High-yield and high-purity isolation of hepatic stellate cells from normal and fibrotic mouse livers," *Nat. Protoc.*, vol. 10, no. 2, p. 305, Jan. 2015, doi: 10.1038/NPROT.2015.017.

[14] X. Han *et al.*, "Mapping the Mouse Cell Atlas by Microwell-Seq," *Cell*, vol. 172, no. 5, pp. 1091-1107.e17, Feb. 2018, doi: 10.1016/J.CELL.2018.02.001.

[15] X. Xiong *et al.*, "Landscape of Intercellular Crosstalk in Healthy and NASH Liver Revealed by Single-Cell Secretome Gene Analysis," *Mol. Cell*, vol. 75, no. 3, pp. 644-660.e5, Aug. 2019, doi: 10.1016/J.MOLCEL.2019.07.028.

[16] N. Schaum *et al.*, "Single-cell transcriptomics of 20 mouse organs creates a Tabula Muris: The Tabula Muris Consortium," *Nature*, vol. 562, no. 7727, p. 367, Oct. 2018, doi: 10.1038/S41586-018-0590-4.

[17] O. Govaere *et al.*, "Transcriptomic profiling across the nonalcoholic fatty liver disease spectrum reveals gene signatures for steatohepatitis and fibrosis," *Sci. Transl. Med.*, vol. 12, no. 572, Dec. 2020, doi: 10.1126/SCITRANSLMED.ABA4448.

[18] L. Pantano *et al.*, "Molecular characterization and cell type composition deconvolution of fibrosis in NAFLD," *Sci. Rep.*, vol. 11, no. 1, Dec. 2021, doi: 10.1038/s41598-021-96966-5.
